# Aquaculture facility-specific microbiota shape the zebrafish gut microbiome

**DOI:** 10.1186/s42523-026-00573-6

**Published:** 2026-05-07

**Authors:** Kayla C. Evens, Ingrid Bakke, Brendan J. M. Bohannan

**Affiliations:** 1 Department of Biology, Institute of Ecology and Evolution, University of Oregon, Eugene, OR USA; 2 Department of Biotechnology and Food Science, Norwegian University of Science and Technology, Trondheim, Norway; 3 Department of Natural Sciences and Environmental Health, University of South-Eastern Norway, Bø i Telemark, Norway

**Keywords:** Zebrafish, Host-microbiome, Environmental microbiome, Aquaculture, 16S rRNA gene sequencing

## Abstract

**Background:**

Environmental microbiomes, such as those in recirculating aquaculture systems (RAS), can play a key role in shaping host-associated microbial communities. In zebrafish (*Danio rerio*) research, these interactions can introduce uncontrolled sources of variation, potentially confounding experimental outcomes across multiple facilities. Despite widespread zebrafish use in microbiome studies, few have characterized the microbial composition of both tank water and fish across multiple independent facilities to evaluate the consequences of environmental microbiome variation on the host microbiome.

**Results:**

We compared water and zebrafish gut microbiomes across five aquaculture facilities two in the United States and three in Norway— using a nested sampling design and 16S rRNA gene sequencing. Alpha diversity was consistently higher in tank water than in fish guts, and beta diversity analyses revealed significant clustering by sample type, facility, and geographic location, with facility identity explaining the largest proportion of compositional variance. Multivariate dispersion also differed significantly across facilities, indicating that observed compositional differences reflect both shifts in community composition and differences in within-facility variability. Each facility harbored a distinct microbial community in both water and fish gut samples, with geographic location further structuring community composition between Oregon and Norwegian facilities. Similarity Percentage analysis identified key taxa driving facility differences, including *Cetobacterium*, *Vibrio*, and *Aeromonas* in fish gut microbiomes and *Pseudomonas* and *Rheinheimera* in tank water. Microbial source tracking using FEAST revealed that facility-level tank water contributed measurably to fish gut microbiome composition in most facilities, though unknown sources dominated estimates across all facilities (71–99%) and the strength of fish-water microbiome association varied substantially across facilities.

**Conclusions:**

This study demonstrates that zebrafish aquaculture facilities harbor unique microbial communities shaped by both environmental and geographic factors. While tank water microbiomes show associations with zebrafish gut microbiome composition, the dominant contribution of unknown sources to gut microbiome composition suggests that factors beyond the immediate tank water environment- including diet, host physiology, and other facility-specific conditions- are primary drivers of gut microbiome variation. The strength of this association varied considerably across facilities and appeared related to fish domestication history, a pattern that warrants direct experimental investigation. These findings underscore the importance of incorporating environmental microbiome assessments into zebrafish experimental design, particularly for studies focused on host-microbe interactions. Without such consideration, unaccounted variation in environmental microbiota may affect microbiome composition and reduce cross-study reproducibility. Moving forward, standardized reporting of environmental conditions and microbial composition across facilities will be critical for strengthening reproducibility and interpretation in zebrafish microbiome research.

**Supplementary Information:**

The online version contains supplementary material available at 10.1186/s42523-026-00573-6.

## Background

Research over the past several decades has highlighted the substantial influence of the microbiome on vertebrate host development and fitness, through mediation of nutrition, metabolism, physiology, and immunology, among others [1, 2]. However, significant variation in the microbiome can be observed both between and within hosts [3, 4]. To better understand why this variability may occur and how it impacts animal host health and function, it is critical to first determine how the microbiome is acquired and maintained.

The structure and composition of the host-microbiome are partially influenced by factors outside of the individual host, which include, but are not limited to, food, interactions with other hosts, and the environmental microbiome [3]. While factors such as diet have been well-studied, the contribution of the environmental microbiome to host-microbiome variation remains unclear. Previous studies have identified correlations between environmental factors and host microbiome variability, but few have directly addressed the potential for the environmental acquisition of microbes by simultaneously assessing both host and environmental microbiomes [5–7].

Zebrafish (*Danio rerio*) are widely used as laboratory animal models for investigating questions related to host-environment interactions due, in part, to their rapid development, high reproductive rate, and ability to be derived germ-free (reared without microbes) [8, 9]. However, the generalizability of conclusions made from zebrafish experiments depends on the reproducibility of results. Studies in laboratory animals, including mice [10, 11] and non-human primates [7, 12], have revealed significant microbiome variation associated with housing condition and vendor sources, even among genotype-matched individuals. If unaccounted for, such variation can confound cross-study comparisons, especially in microbiome-sensitive research.

Similar intra-facility [13] and inter-facility host-microbiome variation [14, 15] has been reported in zebrafish. However, to the best of our knowledge, no studies have directly examined the contribution of the environmental microbiome to zebrafish gut microbiome variability across multiple modern research aquaculture facilities.

Modern zebrafish aquaculture facilities typically use recirculating aquaculture systems (RAS), which, as the name implies, treat and reuse water within a closed-loop system. Over recent decades, interest in these systems has grown in both research and commercial settings, in part due to their efficiency and reduced environmental impact. Studies have demonstrated that these systems may maintain high populations of beneficial bacteria or, at the very least, reduce the abundance of rapid-growing heterotrophic bacteria potentially harmful to fish. This is related to long water retention times, the presence of a biofilter that consumes substrates for bacterial growth, and similar carrying capacities and bacterial densities throughout the system— resulting in selection for slow-growing but competitively superior non-pathogens [16–18]. However, technical specifications for water treatment can vary, with some facilities instead employing flow-through systems, in which all outflow water is discharged rather than recirculated.

Despite a general shared goal of maintaining clean, safe rearing conditions for zebrafish, differences in external inputs and management practices are likely to contribute to variation in water microbial community composition across aquaculture facilities. As such, if zebrafish microbiomes are largely shaped by environmental acquisition, facility-level differences in water microbial communities could drive significant variation in the zebrafish gut microbiome. However, research suggests that the zebrafish intestine is at least somewhat selective, with a “core” intestinal microbiome reported across different facilities [14, 15]. Therefore, selection by the zebrafish gut could mitigate the effect of environmental microbiome variability on the composition of the gut microbiome.

**Fig. 1:**
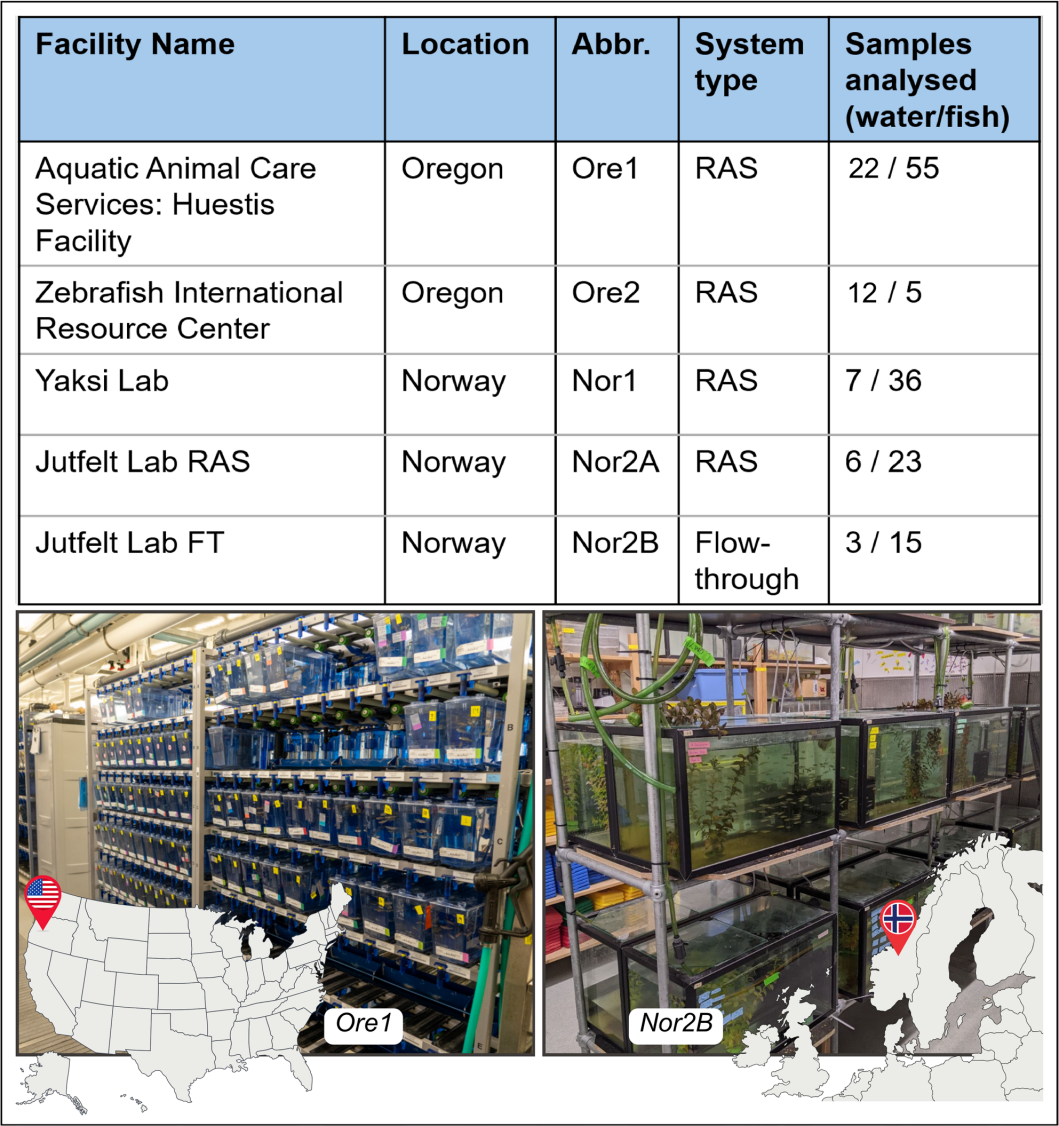
Aquaculture facilities (region, name, abbreviation, and water system type) and samples collected from tank water and zebrafish. The two photos of zebrafish tanks (Ore1- left; Nor2B- right) provide an example of the variety and organization of tanks common in modern aquaculture facilities. Image of Ore1 facility courtesy of Kelley Christensen, 2024, University of Oregon. Image of Nor2B facility, Kayla Evens, 2022

This study addresses two main questions: (1) How do the environmental and fish gut microbiomes vary across aquaculture facilities? and (2) Can we identify patterns of covariation between the environmental microbiome and the zebrafish gut microbiome? To investigate these questions, we selected five facilities in two geographic locations— Trondheim, Norway and Eugene, Oregon—that are representative of typical zebrafish research environments. Within each location, facilities are located within 300 m of each other on the University of Oregon (UO) campus and within 700 m on the Norwegian University of Science and Technology (NTNU) campus (Fig. 1). These facilities predominantly utilize recirculating aquaculture systems (RAS), though one facility (Nor2B) employs a flow-through system with a different water treatment approach. We specifically focused on facilities that supply zebrafish for basic research, as these facilities tend to share operational similarities due to the sensitive nature of rearing laboratory animals, allowing us to better isolate the interaction between host and environmental microbiomes while providing insight into the reproducibility of results from zebrafish sourced from different locations.

To address these questions, we used 16S rRNA gene-sequencing to characterize microbial communities in paired samples of fish and tank water across facilities and applied source tracking approaches to evaluate potential links between environmental and fish-associated microbiomes. 

## Results

### Tank water microbiota

This study aimed to assess how variation in the tank water microbiome influences the zebrafish gut microbiome across aquaculture facilities. Before addressing this, we first determined whether observable variation existed in the tank water microbiome across the five facilities. We analyzed metrics of both community composition and diversity within and across 50 tank water samples, identifying a total of 1,632 unique amplicon sequence variants (ASVs) after normalizing the data to 1,291 reads per sample (*n* = Ore1 (22); Ore2 (12); Nor1 (7); Nor2A (6); Nor2B (3)).

#### Variation in tank water microbiota is driven by shifts in dominant bacterial taxa

**Fig. 2:**
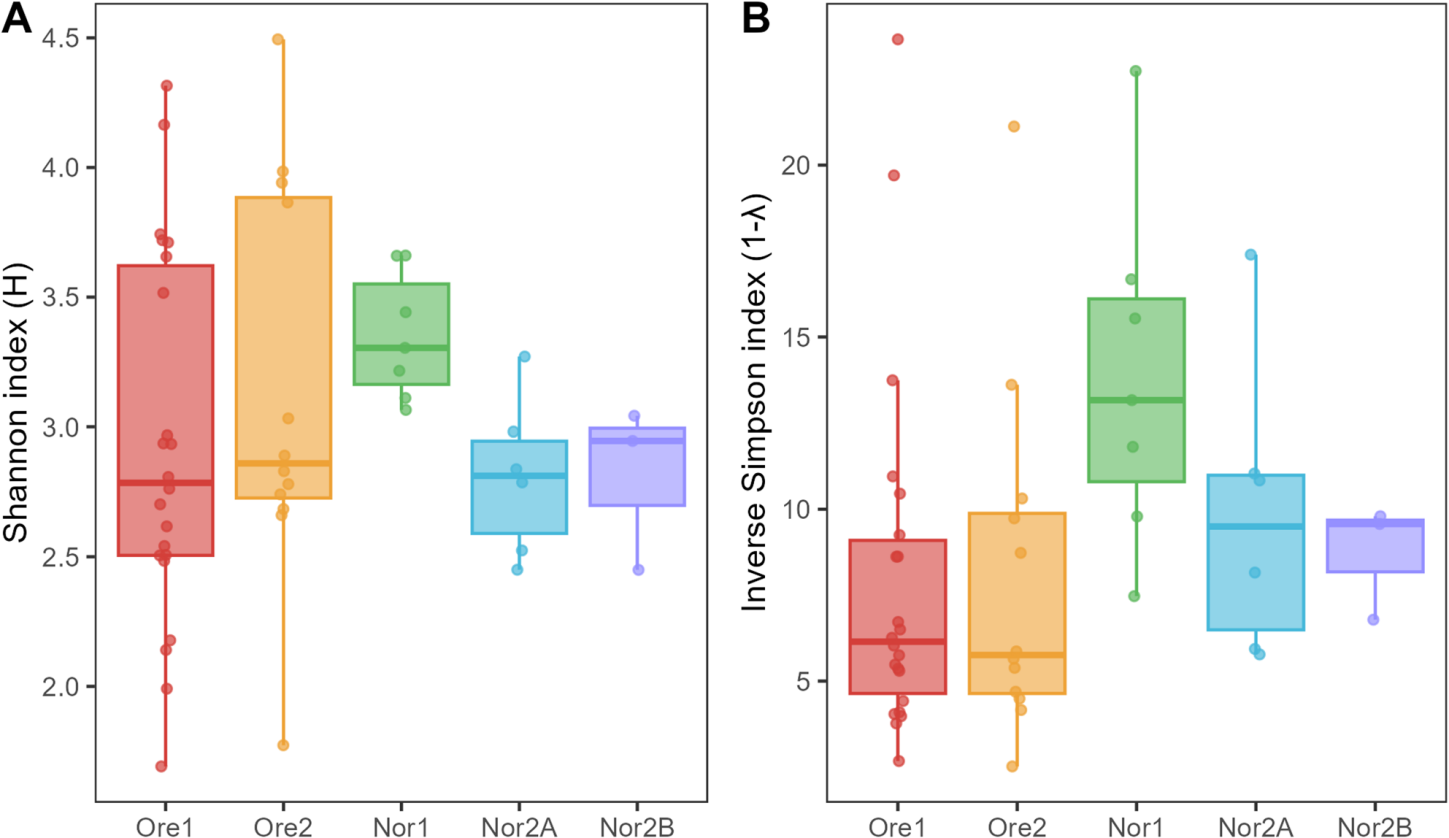
Alpha diversity metrics of tank water microbiota with (**A**) Shannon diversity and (**B**) Inverse Simpson diversity. Tukey-style box and whisker plots display the median (center horizontal line) and interquartile range (upper and lower bounds of the box), while whiskers extend ± 1.5 times the interquartile range; individual points represent single water samples. Neither Shannon nor Inverse Simpson diversity differed significantly across facilities following Benjamini-Hochberg correction of pairwise Wilcoxon rank-sum tests (Shannon: Kruskal-Wallis *p* = 0.233; Inverse Simpson: Kruskal-Wallis *p* = 0.035; no pairwise comparisons reached significance following correction)

To compare water microbial diversity across facilities, we first examined α-diversity metrics. Neither Shannon-Wiener nor Inverse Simpson index values differed significantly across facilities after applying Benjamini-Hochberg correction (BH) to pairwise comparisons (Shannon: Kruskal-Wallis: *p* = 0.233; Inverse Simpson: Kruskal-Wallis: *p* = 0.035). Although the omnibus test for Inverse Simpson diversity was significant, no pairwise comparisons reached significance following correction, with the closest comparisons being Ore1 vs. Nor1 (*p-adj.* = 0.053) and Ore2 vs. Nor1 (*p-adj.* = 0.085) (Fig. 2). Overall, α-diversity did not differ significantly across facilities in water samples.

We further examined tank water microbial community composition across facilities. Twenty-two genera exceeded a mean relative abundance threshold of 1% across all facilities in water samples. Proteobacteria (including Gammaproteobacteria, Betaproteobacteria, and Alphaproteobacteria) was the dominant phylum across all facilities, comprising the majority of abundant genera. Tank water harbored 14 genera exceeding 1% mean relative abundance that did not reach this threshold in fish gut samples, including environmental Proteobacteria and Bacteroidetes such as *Rheinheimera*,* Acinetobacter*,* Acidovorax*,* Flavobacterium*, and *Fluviicola*, though all were detectable at lower abundances in fish gut samples.

**Fig. 3:**
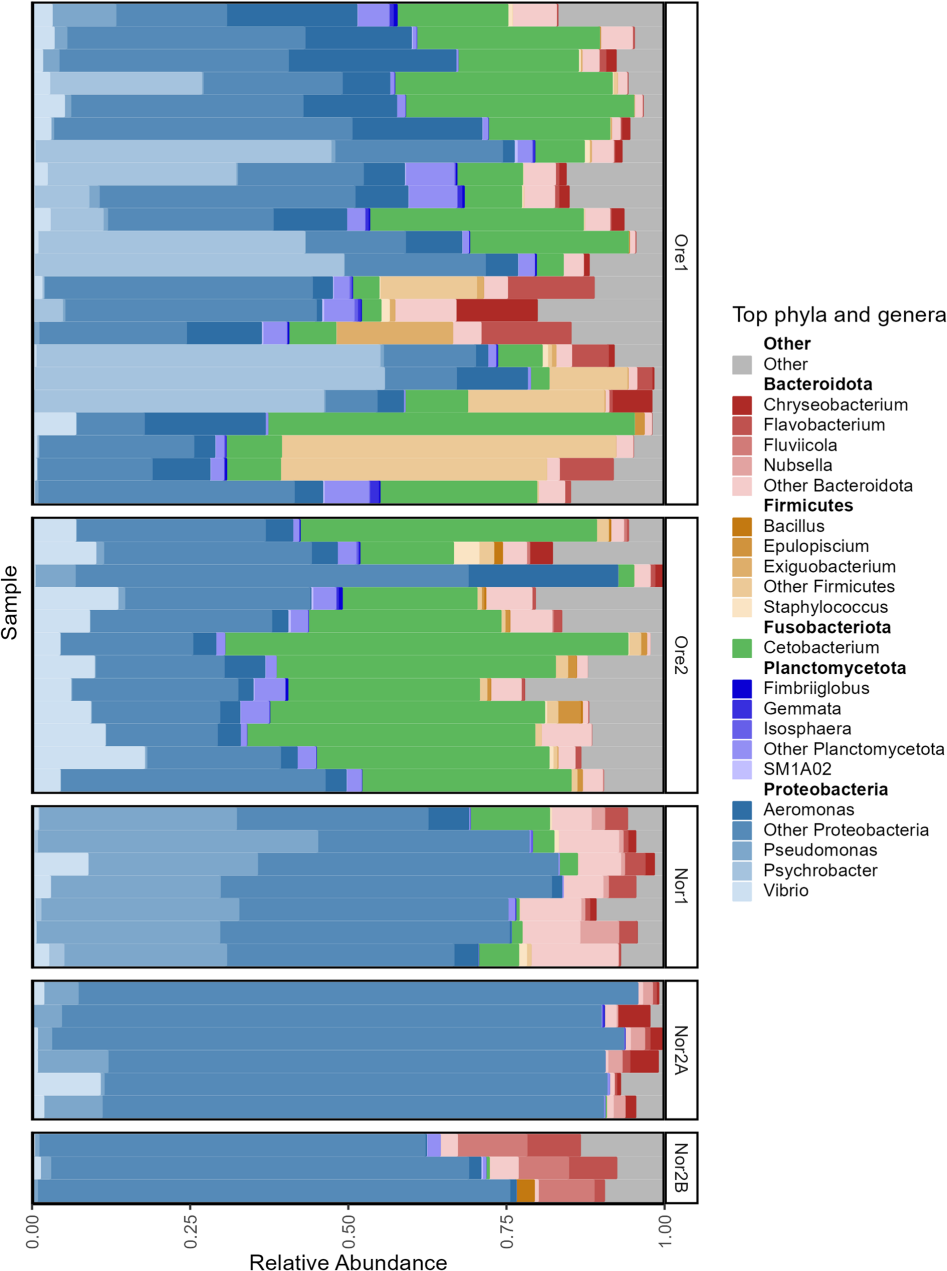
Phylum and genus-level taxonomic composition of tank water microbiome samples across aquaculture facilities. Each bar represents one tank water sample. The mean relative abundance is grouped by aquaculture facility and is displayed for only the top five most abundant phyla and top four genera within each phylum as averaged across samples

The Ore1 and Ore2 facility water microbiomes were both dominated by *Cetobacterium* (Fusobacteria; Ore1: 20.2% and Ore2: 43.1%), with Ore1 further characterized by high relative abundances of *Psychrobacter* (Gammaproteobacteria; 19.3%), *Aeromonas* (Gammaproteobacteria; 11.8%), and *Paracoccus* (Alphaproteobacteria; 7.0%). In contrast, *Pseudomonas* (Gammaproteobacteria) was dominant in Nor1 (35.6%) while Nor2A was characterized by *Acidovorax* (Betaproteobacteria; 11.7%), *Nevskia* (Gammaproteobacteria; 10.6%), and *Limnobacter* (Betaproteobacteria; 9.2%), among others. Nor2B was distinguished by *Rheinheimera* (Gammaproteobacteria; 33.1%), *Acinetobacter* (Gammaproteobacteria; 16.6%), and *Fluviicola* (Bacteroidetes; 10.8%) (Fig. 3).

#### Aquaculture facilities are a major driver of tank water microbial variation

Tank water microbiome composition varied significantly across facilities. Given the nested structure of our study design (facilities nested within geographic locations), we conducted permutational analysis of variance (PERMANOVA) using a hierarchical approach testing the combined effects of location (Norway vs. Oregon), facility, and fish genotype status. Significant effects were detected for all factors (Bray-Curtis and unweighted UniFrac: *p* < 0.001), with location explaining 20.4% and 13.3% of variation in Bray-Curtis and unweighted UniFrac distances, respectively, while individual facility identity accounted for an additional 22.9% and 18.1% of the variation.

To assess whether fish genotype independently influenced water microbiome composition after accounting for facility-level differences, we performed a constrained PERMANOVA with permutations restricted within each facility. This approach was necessary because genotype and facility are inherently confounded in this study design, with specific genotypes housed within specific facilities (see Table S6). Genotype status was non-significant when permutations were constrained within facilities (Bray-Curtis: *p* = 0.429; unweighted UniFrac: *p* = 0.288), indicating that the apparent genotype effect in the unconstrained hierarchical model likely reflects facility-level differences rather than a direct biological effect of fish genetics on the water microbiome composition (Table S2).

Pairwise PERMANOVA comparisons revealed significant compositional differences between all facility pairs for both Bray-Curtis and unweighted UniFrac distances (all *p-adj.* ≤ 0.012), with stronger differentiation observed between geographic locations than within locations. The weakest differentiation was observed between Nor2A and Nor2B (Bray-Curtis: R^2^ = 0.374, *p*-adj. = 0.010; unweighted UniFrac: R^2^ = 0.417, *p-adj.* = 0.012), consistent with their shared geographic location, though these communities remained statistically distinguishable (Table 1).

**Table 1:** Pairwise PERMANOVA on Bray-Curtis and unweighted UniFrac distances of aquaculture facility water microbiome data. p-value adjusted with Benjamini-Hochberg correction

Pairs	Df	SumsOfSqs	F.Model	R2	*p*.value	*p*.adjusted	sig
**Bray-Curtis**							
Ore1 vs. Ore2	1	1.897	9.169	0.223	0.0001	0.0002	**
Ore1 vs. Nor2A	1	2.502	10.476	0.287	0.0001	0.0002	**
Ore1 vs. Nor2B	1	1.618	6.949	0.232	0.0003	0.0005	**
Ore1 vs. Nor1	1	2.440	10.693	0.284	0.0001	0.0002	**
Ore2 vs. Nor2A	2	2.521	6.819	0.476	0.0001	0.0002	**
Ore2 vs. Nor2B	1	1.699	11.520	0.470	0.0029	0.0036	*
Ore2 vs. Nor1	1	2.331	14.549	0.461	0.0001	0.0002	**
Nor2A vs. Nor2B	1	0.901	4.185	0.374	0.0104	0.0104	.
Nor2A vs. Nor1	1	1.537	7.305	0.399	0.0005	0.0007	**
Nor2B vs. Nor1	1	1.389	7.617	0.488	0.0076	0.0084	*
**Unweighted UniFrac**							
Ore1 vs. Ore2	1	1.088	5.663	0.150	0.0001	0.0002	**
Ore1 vs. Nor2A	1	1.673	9.641	0.271	0.0001	0.0002	**
Ore1 vs. Nor2B	1	0.855	4.584	0.166	0.0004	0.0007	**
Ore1 vs. Nor1	1	1.055	5.676	0.174	0.0001	0.0002	**
Ore2 vs. Nor2A	2	1.411	3.709	0.331	0.0001	0.0002	**
Ore2 vs. Nor2B	1	0.731	3.611	0.217	0.0022	0.0028	*
Ore2 vs. Nor1	1	0.871	4.404	0.206	0.0001	0.0002	**
Nor2A vs. Nor2B	1	0.712	4.999	0.417	0.0123	0.0123	.
Nor2A vs. Nor1	1	0.867	5.524	0.334	0.0006	0.0009	**
Nor2B vs. Nor1	1	0.649	3.451	0.301	0.0077	0.0086	*

Multivariate dispersion differed significantly across facilities for both Bray-Curtis (*p* = 0.011) and unweighted UniFrac distances (*p* = 0.013), indicating that PERMANOVA differences reflect both shifts in community composition and differences in within-facility variability. Pairwise dispersion comparisons revealed that Ore1 exhibited significantly greater Bray-Curtis dispersion than Ore2, Nor1, and Nor2B (all *p-adj.* ≤ 0.018), while Nor2A exhibited significantly lower unweighted UniFrac dispersion than Ore1, Ore2, and Nor1 (all *p-adj.* ≤ 0.034), suggesting Nor2A may harbor a phylogenetically constrained water microbiome relative to other facilities.

**Fig. 4:**
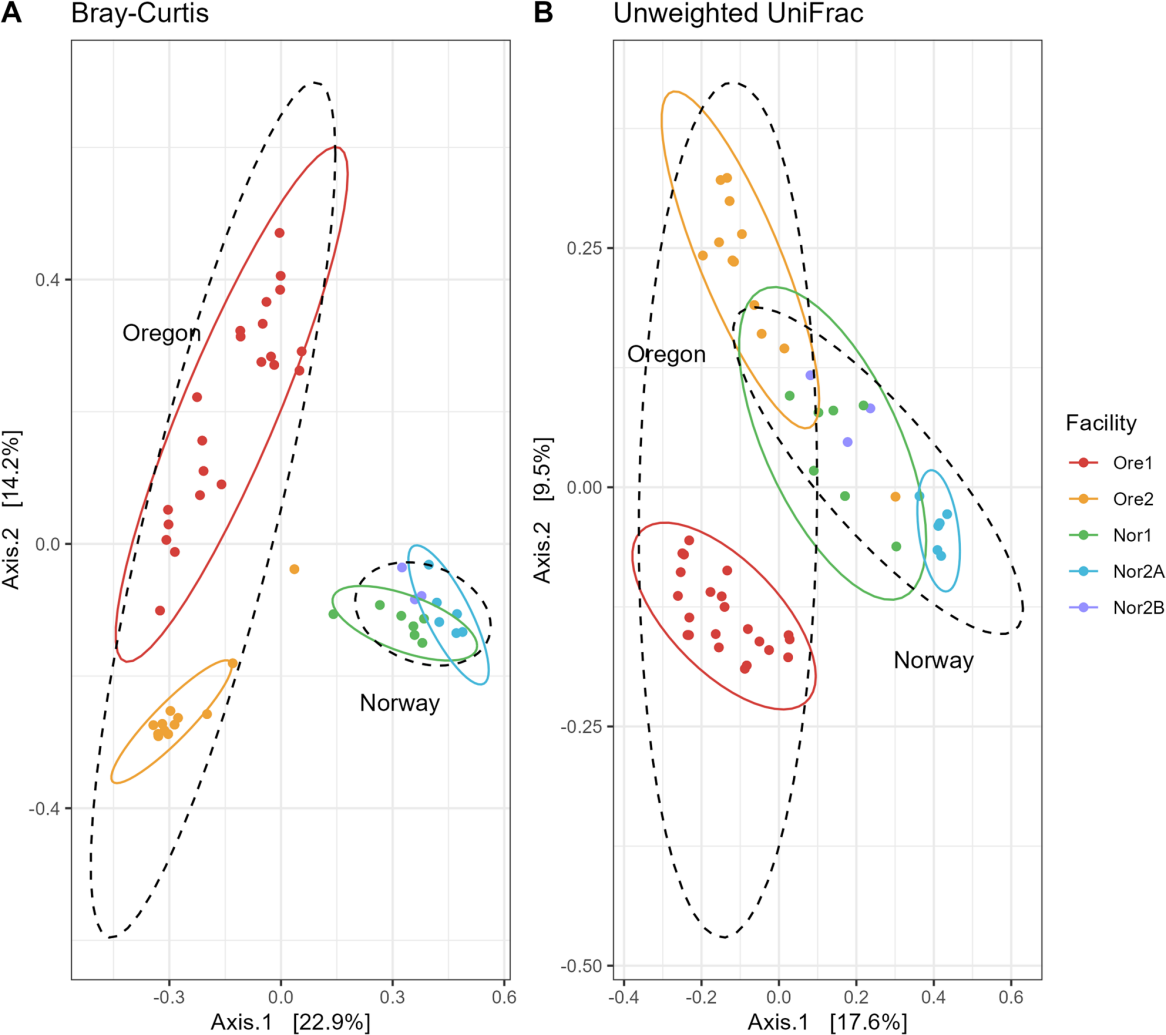
Beta diversity of tank water microbiota across aquaculture facilities. Principal coordinate analysis (PCoA) of (**A**) Bray-Curtis and (**B**) unweighted UniFrac dissimilarity. Colors and solid ellipses represent individual facilities (95% confidence ellipses). Dashed ellipses indicate geographic location groupings (Oregon and Norway). Individual points represent single water samples, with one sample collected per tank

PCoA plots of Bray-Curtis and unweighted UniFrac distances further corroborated these results, with tank water microbiomes clustering strongly by facility, though some overlap was observed among Norwegian facilities (Fig. 4).

To further investigate which genus-level taxa most contributed to differences observed between aquaculture facilities, we performed a Similarity Percentage (SIMPER) analysis [19].

The SIMPER analysis revealed that differences in tank water microbiota among facilities were primarily characterized by shifts in members of the Fusobacteria, Proteobacteria, and Bacteroidetes phyla. As SIMPER analysis is weighted towards numerically dominant taxa, these results reflect differences in the most abundant community members rather than comprehensive drivers of compositional variation. *Cetobacterium* (Fusobacteria) was a major contributor to dissimilarity in comparisons involving Ore2, accounting for 14.9–20.9% of dissimilarity across Ore2 facility pairs but was absent from the Ore1 vs. Norwegian facility comparisons after BH correction, suggesting *Cetobacterium* is particularly associated with Ore2 water microbiota. *Pseudomonas* (Gammaproteobacteria) was the dominant contributor to all comparisons involving Nor1 (14.9–18.1%), while *Rheinheimera* (Gammaproteobacteria) was the primary driver of dissimilarity in all comparisons involving Nor2B (13.4–17.2%), consistent with the high relative abundance of *Rheinheimera* observed in Nor2B water samples (33.1%). Comparisons involving Nor2A were characterized by multiple Betaproteobacteria taxa, particularly *Acidovorax* and *Limnobacter*. These results suggest that facility-specific taxa are key drivers of within-region water microbiome differences, with *Pseudomonas*, *Rheinheimera*, and *Cetobacterium* each characterizing distinct facilities (Table S3).

### Zebrafish gut microbiota

To determine whether zebrafish gut microbiomes varied across tanks and facilities, we applied similar analyses of community composition and diversity. Across 134 fish samples, we identified 1,267 unique ASVs after data were normalized to 1,171 reads per sample (*n* = Ore1 (55); Ore2 (5); Nor1 (36); Nor2A (23); Nor2B (15)).

#### Zebrafish gut microbiota exhibit facility-specific diversity and compositional patterns

**Fig. 5:**
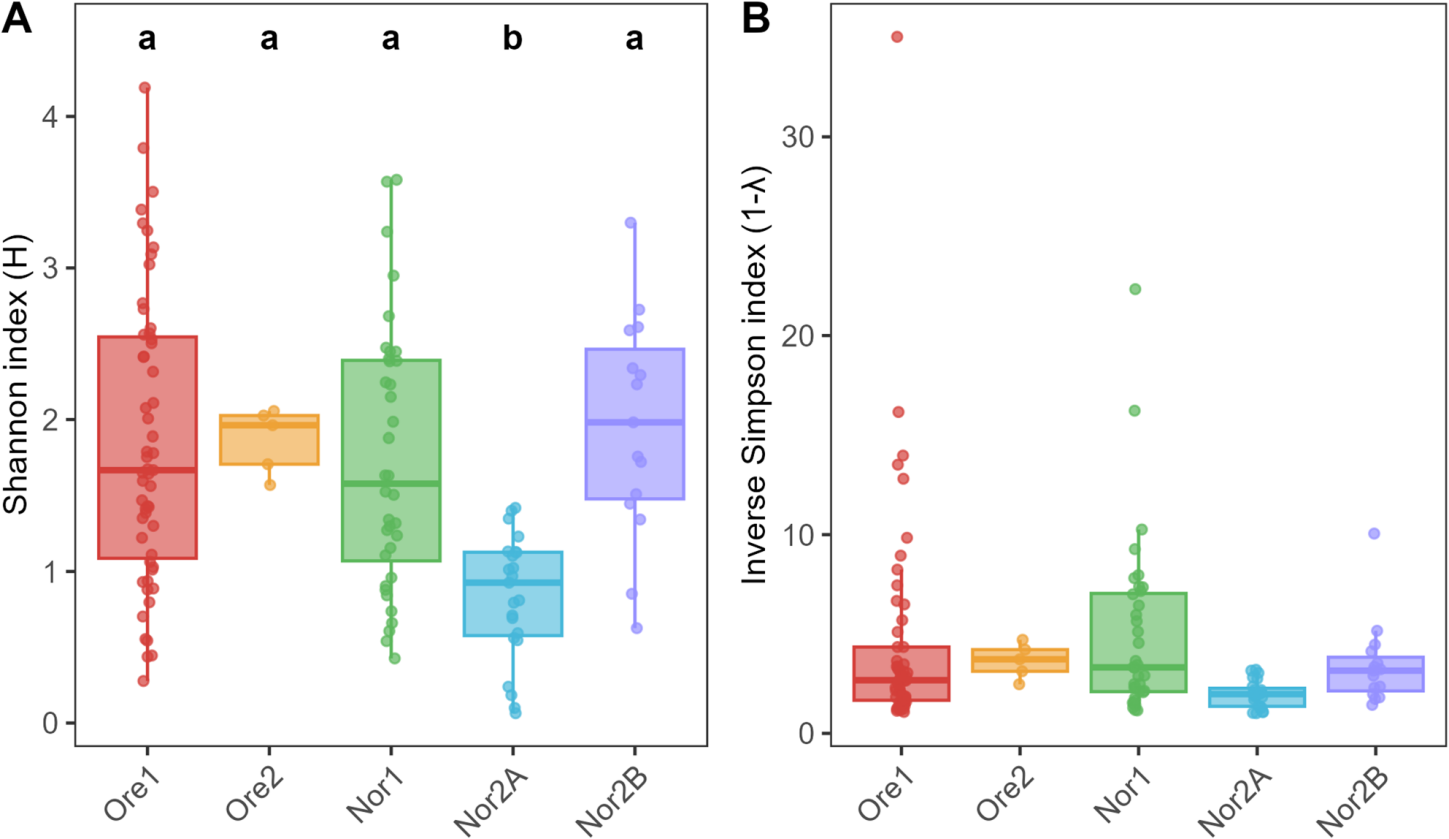
Alpha diversity metrics of zebrafish gut microbiota with (**A**) Shannon diversity and (**B**) Inverse Simpson diversity. Tukey-style box and whisker plots display the median (center horizontal line) and interquartile range (upper and lower bounds of the box), while whiskers extend +/- 1.5 times the interquartile range. Significance was assessed using linear mixed models with tank included as a random effect to account for non-independence of fish within tanks. Post-hoc pairwise comparisons were performed using estimated marginal means with Benjamini-Hochberg p-value correction; facilities sharing a letter do not differ significantly (*p-adj.* > 0.05). Nor2A (b) had significantly lower Shannon diversity than Nor1, Nor2B, and Ore1 (all *p-adj.* ≤ 0.006). No significant pairwise differences were detected for Inverse Simpson diversity. One Ore1 sample with elevated Inverse Simpson diversity was retained in all analyses following quality control review

Zebrafish gut microbiomes varied significantly in Shannon α-diversity across facilities (linear mixed model with tank as random effect: *p* = 0.003), while Inverse Simpson diversity did not differ significantly (*p* = 0.219). Post-hoc pairwise comparisons with BH correction revealed that Shannon diversity was significantly lower in Nor2A compared to Nor1 (*p-adj.* = 0.006), Nor2B (*p-adj.* = 0.006), and Ore1 (*p-adj.* = 0.001), while all other facility pairs were statistically indistinguishable (see Table S1 for full pairwise comparisons). Alpha diversity was consistently lowest in the Nor2A facility (Fig. 5). Sensitivity analyses excluding the underrepresented Ore2 facility (*n* = 5 fish, single tank) yielded qualitatively identical results (see Supplementary Material 5).

Thirteen genera exceeded a mean relative abundance threshold of 1% across all facilities in fish gut microbiome samples, four of which (*Cetobacterium*, *Aeromonas*, *Vibrio*, and *Plesiomonas*) exceeded this threshold in all five facilities. Five genera exceeded 1% mean relative abundance exclusively in fish gut samples- *Streptococcus*, *Enterococcus*, *Lactococcus*, *Bacillus*, and *Staphylococcus*- three of which (*Lactococcus*,* Streptococcus*, and *Enterococcus*) belong to the order Lactobacillales (phylum Firmicutes), suggesting enrichment of Firmicutes in the fish gut environment relative to tank water.

**Fig. 6:**
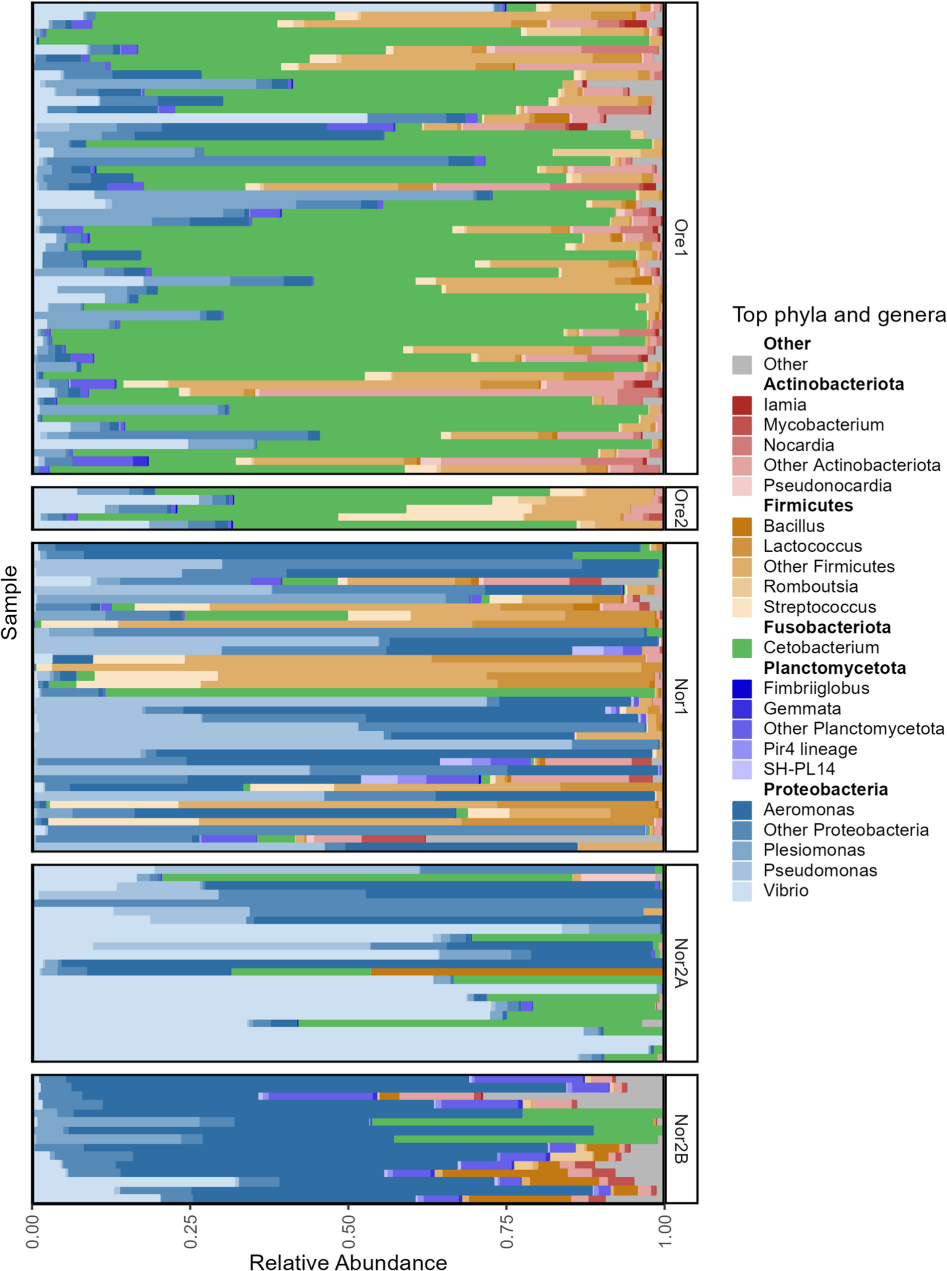
Phylum and genus-level taxonomic composition of zebrafish gut microbiome samples across aquaculture facilities. The mean relative abundance is grouped by aquaculture facility and is displayed for only the top five most abundant phyla and top four genera within each phylum as averaged across samples

In the Oregon facilities, fish gut microbiomes were dominated by *Cetobacterium* (Ore1: 55.6%; Ore2: 48.6%), with Ore2 also distinguished by notably higher relative abundances of *Streptococcus* (12.5%) and *Enterococcus* (11.4%) compared to other facilities. Among Norwegian facilities, *Aeromonas* was the dominant genus in Nor2B (60.4%) and was also abundant in Nor1(22.5%) and Nor2A (15.9%). Nor2A was dominated by *Vibrio* (44.5%), while Nor1 was characterized by relatively higher abundances of *Pseudomonas* (18.6%) and *Aeromonas* (22.5%) compared to other Norwegian facilities (Fig. 6).

#### Aquaculture facility strongly influences zebrafish gut microbiome variation

**Fig. 7:**
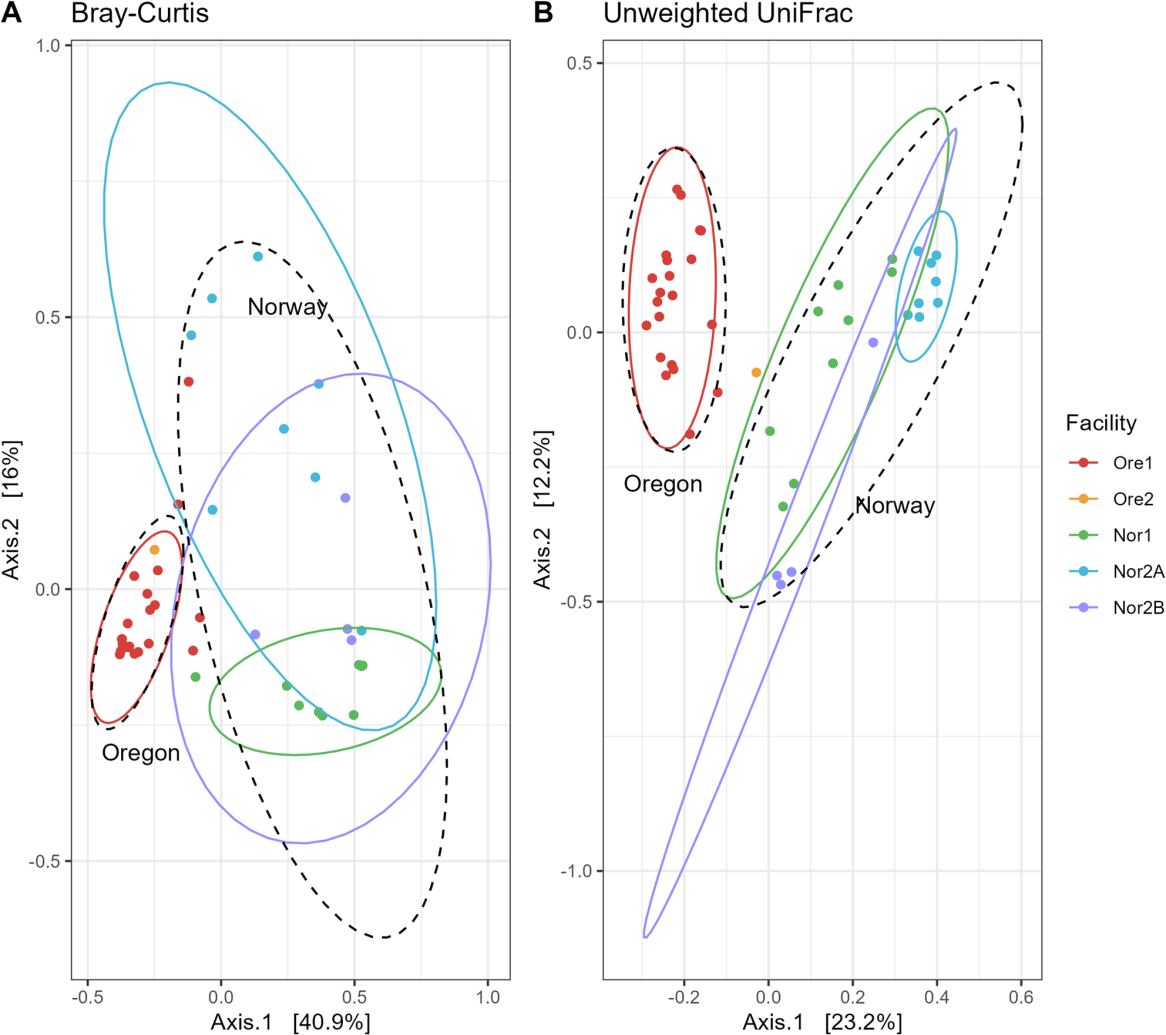
Beta diversity of zebrafish gut microbiota across aquaculture facilities. Principal coordinate analysis (PCoA) of (**A**) Bray-Curtis and (**B**) unweighted UniFrac dissimilarity. Colors and solid ellipses represent individual facilities (95% confidence ellipses). Dashed ellipses indicate geographic location groupings (Oregon and Norway). Individual points represent tank-averaged community profiles

Compositional differences in zebrafish microbiomes were visualized using a PCoA based on Bray-Curtis and unweighted UniFrac dissimilarity (Fig. 7). Beta-diversity differed significantly by both geographic location and facility (PERMANOVA; Bray–Curtis: Location: R^2^ = 0.323, *p* < 0.001; Location: Facility: R^2^ = 0.165, *p* < 0.001 and unweighted UniFrac: Location: R^2^ = 0.201, *p* < 0.001; Location: Facility: R^2^ = 0.147, *p* < 0.001), based on tank-level aggregated community profiles. Location explained a greater proportion of variance in fish gut microbiome composition than in tank water samples (Bray-Curtis: R^2^ = 0.323 vs. 0.204: unweighted UniFrac: R^2^ = 0.201 vs. 0.133), suggesting that geographic effects on the gut microbiome may exceed those on the surrounding tank water environment.

As observed for tank water samples, genotype status appeared significant in the unconstrained hierarchical model (Bray-Curtis: *p* = 0.006; unweighted UniFrac: *p* < 0.001) but was non-significant when permutations were constrained within facilities (Bray-Curtis: *p* = 0.376; unweighted UniFrac: *p* = 0.434), indicating that we cannot distinguish a genotype effect from facility-level confounding in the current dataset. While these results suggest that facility rather than genotype is the primary driver of gut microbiome composition, a genuine effect of genotype cannot be excluded and warrants investigation in future studies with dedicated genotype replication across facilities (Table S4).

Pairwise PERMANOVAs with BH correction confirmed significant compositional differences between all non-Ore2 facility pairs for both Bray-Curtis (all *p-adj.* ≤0.030) and unweighted UniFrac distances (all p*-adj.* ≤ 0.005). All comparisons involving Ore2 were non-significant across both metrics, consistent with limited statistical power due to the small sample size of that facility (*n* = 5 fish, one tank). Sensitivity analyses excluding Ore2 yielded qualitatively identical results for all remaining facility pairs (Supplementary Material 5).

Multivariate dispersion differed significantly across facilities for both Bray-Curtis (*p* = 0.024) and unweighted UniFrac distances (*p* < 0.001), indicating that PERMANOVA differences reflect both shifts in community composition and differences in within-facility variability. Pairwise Bray-Curtis dispersion comparisons revealed that Ore1 exhibited significantly greater dispersion than Nor1 (*p* = 0.045), while Nor1 exhibited significantly greater dispersion than Nor2B (*p* = 0.029). Although overall unweighted UniFrac dispersion differed significantly across facilities, no individual pairwise comparisons reached significance following correction, suggesting that dispersion effects are distributed across multiple facility pairs rather than driven by a single outlier facility.

The SIMPER analysis of fish microbiota revealed that facility differences were primarily characterized by shifts in members of the Fusobacteria and Proteobacteria phyla. Following BH p-value correction for multiple comparisons within each facility pair, the number of significantly contributing taxa varied substantially across comparisons. No taxa significantly contributed to dissimilarity in Ore1 vs. Ore2, Ore2 vs. Nor1, or Ore2 vs. Nor2A comparisons, following BH correction, consistent with the absence of significant PERMANOVA results for Ore2 facility pairs and the limited statistical power associated with this facility’s small fish sample size.

In Oregon vs. Norway comparisons, *Cetobacterium* (Fusobacteria) was a primary contributor to dissimilarity between Ore1 and Norwegian facilities, accounting for 25.1% of Ore1 vs. Nor1 dissimilarity (*p-adj.* = 0.005) and 25.7% of Ore1 vs. Nor2B dissimilarity (*p-adj.* = 0.005). *Vibrio* (Gammaproteobacteria) was the sole significant contributor to Ore1 vs. Nor2A dissimilarity after correction (20.9%, *p-adj.* = 0.033), while *Aeromonas* (Gammaproteobacteria) was the top contributor to Ore1 vs. Nor2B dissimilarity (27.5%, *p-adj.* = 0.002).

Among Norwegian facility comparisons, *Vibrio* was the dominant contributor to Nor2A vs. Nor1 dissimilarity (22.1%, *p-adj.* = 0.011). *Aeromonas* was the primary significant contributor to Nor2A vs. Nor2B dissimilarity (20.1%, *p-adj.* = 0.040), while Nor2B vs. Nor1 comparisons were characterized by lower-abundance taxa after correction, with no single dominant contributor emerging. These findings suggest that *Cetobacterium*, *Vibrio*, and *Aeromonas* are key drivers of facility-level compositional differences in zebrafish gut microbiota across facilities with sufficient statistical power to detect them, consistent with the dominance of these taxa observed in facility-specific relative abundance profiles (Table S5).

### Covariation between zebrafish gut and water microbiomes

Once we confirmed significant variation in both fish and water microbiomes across facilities, we examined covariation between fish gut and water microbiomes using matched samples collected from the same tanks, source tracking analyses, and combined community-level comparisons. For combined analyses of fish gut and tank water microbiomes, all samples were re-rarefied to a common threshold of 1,171 reads— the minimum read count among retained fish gut samples— to ensure equivalent sequencing depth across sample types for direct comparisons.

#### Fish and water microbiomes facility-specific patterns of compositional overlap

Across both sample types, 471 total genera were detected, of which 178 were shared between fish gut and water microbiomes. Despite this overlap, fish gut and water microbiomes harbored comparable numbers of exclusive genera— 147 found only in fish gut and 146 found only in water samples reflecting compositionally distinct communities despite shared genus-level diversity. Shared genera were predominantly Proteobacteria (78 genera), followed by Actinobacteriota (29), Firmicutes (19), Bacteroidota (14), and Planctomycetota (14). Eight genera exceeded 1% mean relative abundance in both sample types: *Cetobacterium* (fish: 26.3%, water: 13.6%), *Aeromonas* (fish: 20.7%, water: 4.2%), *Vibrio* (fish: 14.0%, water: 4.0%), *Pseudomonas* (fish: 5.6%, water: 9.0%), *Plesiomonas* (3.8%, 1.8%), *Delftia* (1.3%, 2.5%), *Stenotrophomonas* (1.1%, 1.7%), and *Shewanella* (1.0%, 2.1%).

**Fig. 8:**
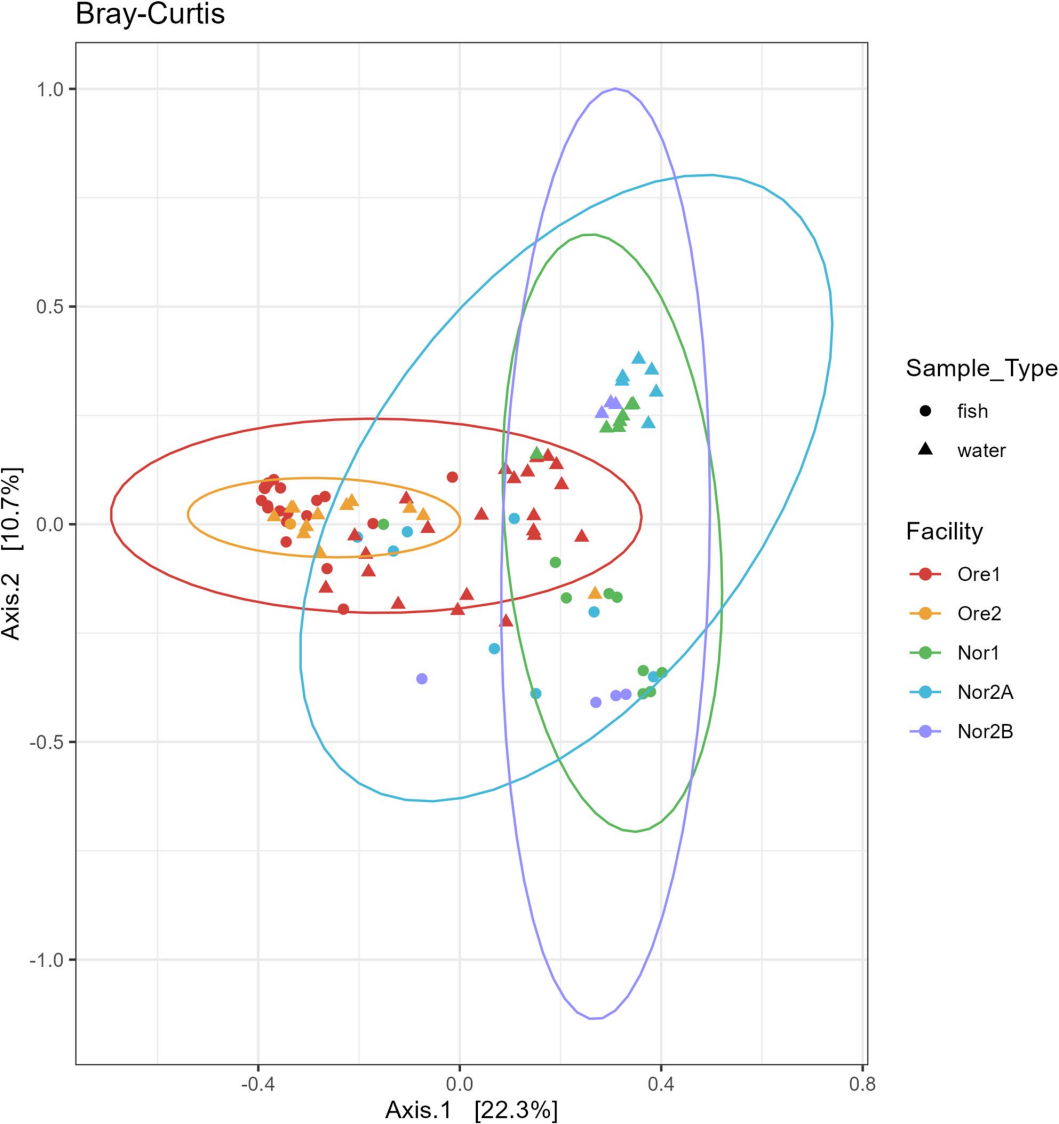
Beta diversity of zebrafish gut and tank water microbiota across aquaculture facilities. Principal coordinate analysis (PCoA) of Bray-Curtis dissimilarity for combined fish gut (circles) and tank water (triangles) samples. Colors represent individual facilities. Solid ellipses represent 95% confidence intervals for each facility. Individual fish gut points represent tank-averaged community profiles; individual water points represent single water samples with one sample collected per tank. Bray-Curtis dissimilarity was used exclusively for combined analyses as calculation of UniFrac distances requires a shared phylogenetic tree, which could not be constructed across both sample types without substantial reduction in taxa representation

Zebrafish gut and tank water microbiomes differed significantly in community composition (PERMANOVA; Sample Type: R² = 0.073, *p* < 0.001; Facility: R² = 0.291, *p* < 0.001). A significant Sample Type × Facility interaction was also detected (R² = 0.162, *p* < 0.001), indicating that the degree of divergence between fish gut and water microbiomes varied across facilities. Pairwise comparisons revealed significant fish-water divergence within Ore1 (*p-adj*. = 0.002), Nor1 (*p-adj.* = 0.003), Nor2A (*p-adj*. = 0.002), and Nor2B (*p-adj.* = 0.030), while Ore2 did not reach significance (*p-adj.* = 0.316), consistent with limited statistical power for that facility. Facility identity explained a substantially greater proportion of variance than sample type, suggesting that geographic and facility-level factors are the primary drivers of microbiome composition across both sample types. Multivariate dispersion differed significantly between sample types (*p* = 0.012) and across facilities (*p* < 0.001), indicating that observed compositional differences reflect both shifts in community composition and differences in within-group variability. Sensitivity analyses excluding Ore2 yielded qualitatively identical results, with significant fish-water divergence detected within all four remaining facilities (all *p-adj.* ≤ 0.034; Supplementary Material 5) (Fig. 8).

To further characterize the relationship between fish gut and tank water microbiomes, we examined pairwise Bray-Curtis dissimilarity across three comparison categories: same tank water, water from the same facility but a different tank, and water from other facilities, using tank-averaged fish community profiles. Ore2 was excluded from this analysis due to single-tank representation precluding meaningful within-facility comparisons. Results are presented descriptively as inherent imbalance in the number of pairwise comparisons across categories precluded formal significance testing. The relationship between fish gut microbiome composition and geographic proximity to tank water varied across facilities, with no consistent pattern of greater similarity to same-facility water observed across all facilities (Figure S1).

#### The influence of the water microbiome on zebrafish gut microbiomes varies by facility

To assess the extent to which the tank water microbiome contributes to the zebrafish gut microbiome, we used Fast Expectation-Maximization Microbial Source Tracking (FEAST), a probabilistic modeling approach that estimates the proportion of microbial taxa in a given sample (the “sink”) that can be attributed to potential defined source environments [20]. The unknown fraction in each analysis represents microbial sources not captured by facility tank water, including potential contributions from diet, host physiology, or other facility-specific environmental factors.

**Fig. 9:**
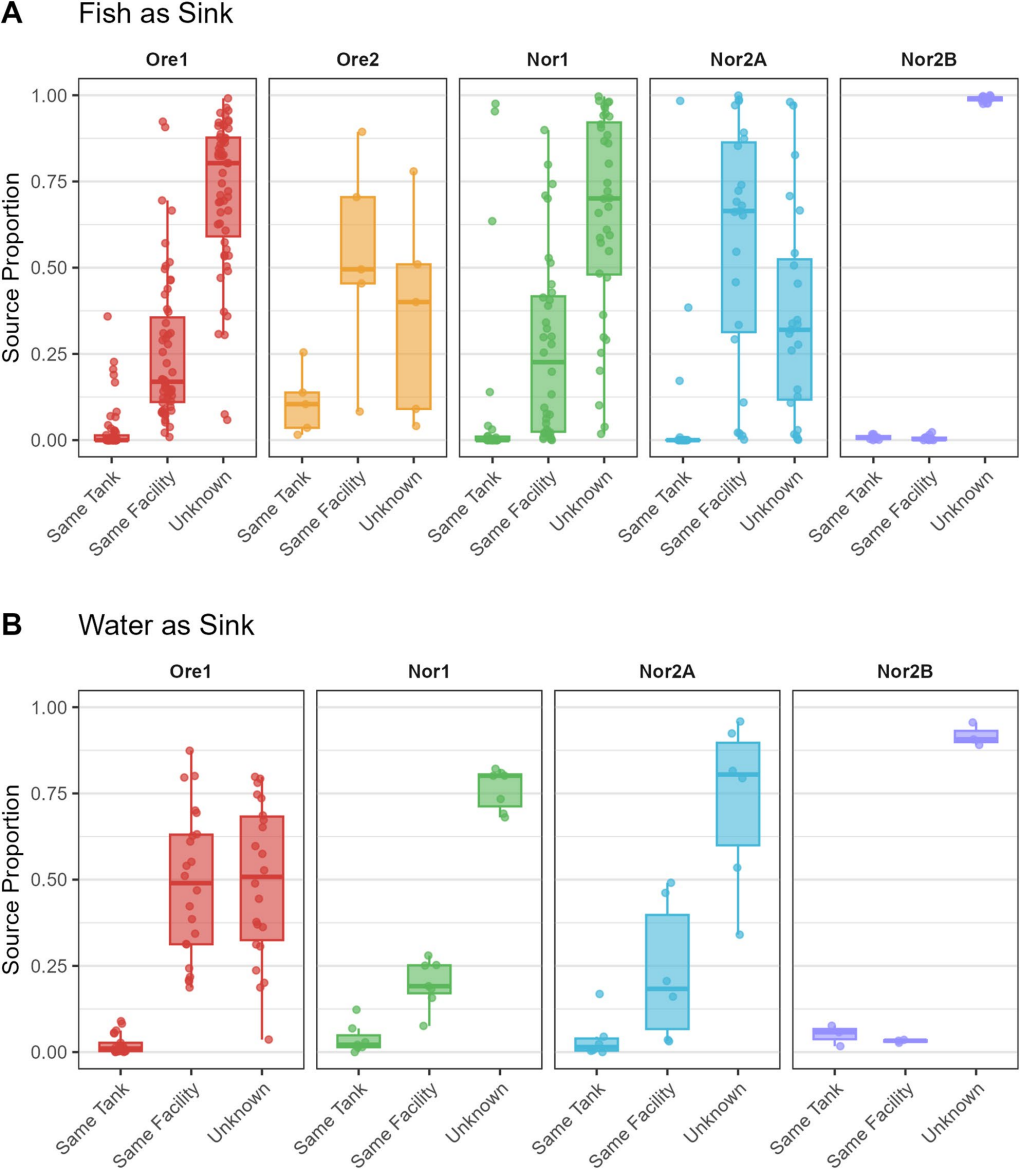
Microbial source tracking of zebrafish gut and tank water microbiomes across aquaculture facilities using Fast Expectation-Maximization for Microbial Source Tracking (FEAST). (**A**) Fish gut microbiomes classified as sinks with facility tank water as sources. (**B**) Tank water microbiomes classified as sinks with facility fish gut microbiomes as sources; Ore2 is excluded from panel B as only one of twelve water samples had a corresponding fish gut sample from the same tank, precluding meaningful facility-level interpretation. Source contributions are categorized as same tank (water or fish from the same tank as the sink sample), same facility (water or fish from other tanks within the same facility), or unknown (sources not captured by facility tank water or fish gut samples, potentially including diet, host physiology, or other facility-specific environmental factors). Individual points represent single sink samples; box and whisker plots display the median (center horizontal line) and interquartile range, with whiskers extending ± 1.5 times the interquartile range. Results are presented descriptively as the compositional nature of source proportions and non-independence of samples within facilities precluded formal significance testing

When fish gut microbiomes were classified as sinks, the unknown fraction dominated across all facilities, ranging from 71.5% in Ore1 to 98.9% in Nor2B, indicating that tank water explains only a minority of fish gut microbiome composition at all facilities. Facility tank water nonetheless contributed meaningfully in some facilities— same-facility water contributed a mean of 25.6% in Ore1, 26.7% in Nor1, and 57.2% in Nor2A— while contributions from the specific same-tank water sample were consistently low across all facilities (range: 0.8–9.7%). Nor2B was a striking exception, with same-facility water contributing essentially nothing (0.5%) and unknown sources accounting for 98.9% of gut microbiome composition, suggesting that Nor2B fish gut microbiomes are largely decoupled from the local water environment. This is consistent with the pronounced compositional divergence between Nor2B fish gut microbiomes (dominated by *Aeromonas*, 60.4%) and Nor2B tank water (dominated by *Rheinheimera*, 33.1%) observed in relative abundance analyses (Fig. 9A).

In the reverse analysis with water classified as the sink, same-facility fish gut microbiomes contributed moderately to water microbiome composition in Ore1 (48.4%) but less so in Norwegian facilities (Nor1: 19.9%, Nor2A: 23.1%), while Nor2B again showed minimal fish-to-water contribution (3.2%) with unknown sources dominating (91.8%). Ore2 is excluded from the water-as-sink analysis as only one of twelve water samples had corresponding fish gut samples from the same tank, precluding meaningful facility-level interpretation. Same-tank contributions were consistently low in both directions across all facilities (Fig. 9B).

## Discussion

In this study, we aimed to characterize microbial variation across aquaculture facilities and assess the extent of microbial association between zebrafish gut and water microbiomes. By analyzing paired tank water and zebrafish gut microbiomes from five research-focused aquaculture facilities, we found that facility-specific factors strongly shape microbial community composition in both fish and water. While water and fish microbiomes remain compositionally distinct, many of the most abundant microbial taxa are shared between the two, and fish gut microbiome composition differed significantly from water microbiomes within the same facility. Source tracking analyses further suggest that facility-level water microbiomes are associated with zebrafish gut microbiome composition, though the strength of this association varies considerably across facilities and unknown sources dominate gut microbiome composition at all sites. Reciprocal analyses indicate that microbial exchange may be bidirectional in some facilities, with fish also contributing to the surrounding water microbiome.

### Variation in facility water microbiota

Beta-diversity analyses of the facility water microbiomes revealed clear differences in microbiome composition between locations (Oregon vs. Norway) as well as among facilities within each geographic region. A parsimonious explanation for the broad Oregon-Norway divide is biogeographic— water sourced from geographically distinct regions inherently harbors distinct environmental bacterial communities, shaped by local climate, geology, and hydrology [21, 22]. Facility-specific operational factors likely further structure these communities within each region, as discussed below. Examining the community composition provides additional clues to the driving forces behind these differences. Across all facilities, two phyla— Proteobacteria and Fusobacteriota accounted for more than 75% of total reads in most samples. While Proteobacteria were represented by multiple genera, Fusobacteriota was almost exclusively represented by a single genus, *Cetobacterium*, assigned to a single ASV (ASV1). The Oregon facilities, in particular, were dominated and differentiated by the relative abundance of *Cetobacterium*.

*Cetobacterium* has been identified as a “core” member of the zebrafish gut microbiome [14, 15] as well as in a wide range of other freshwater fish species [23–25]. Consistent with this host-association, our data showed *Cetobacterium* at substantially higher relative abundances in fish samples than in water samples across all facilities. In zebrafish, *Cetobacterium* appears to be enriched in the intestines of adults [26] and has been associated with potential host benefits, including glucose homeostasis, reduced parasite burden, and probiotic effects on liver health [27–29]. This pattern suggests that the *Cetobacteria* detected in water samples are likely host-associated, originating from or strongly amplified by zebrafish hosts in this study system, consistent with source tracking results indicating fish gut microbiota contribute meaningfully to water microbiome composition in Ore1.

Other highly prevalent taxa in the Oregon facility water samples, including *Psychrobacter* and *Vibrio*, are also widely associated with fish microbiomes [14, 28]. While some *Vibrio* species are known pathogens [30], this diverse genus includes many species that are common associates of healthy fish and aquatic environments, with some even showing potential probiotic properties in fish [31]. This pattern is similar to how *E. coli* can be both commensal and pathogenic in humans— the genus encompasses both beneficial and harmful members. Source tracking analyses indicated that fish microbiota accounted for a mean of 48.4% of the water microbiome composition in Ore1, suggesting that microbial inputs from zebrafish may play a strong role in shaping the water microbiome in this facility, though the direction and mechanism of this relationship cannot be established from observational data alone.

In contrast, the Norwegian facilities were dominated by Proteobacteria, though the most abundant taxa varied by facility. Many of these taxa are commonly found in open water and sediments, and their associations with fish are context-dependent appearing primarily under conditions of stress, disease, or environmental perturbation rather than as consistent members of fish microbiomes [32]. For instance, Nor2B water was dominated by *Rheinheimera* and *Acinetobacter*. *Rheinheimera *is commonly detected in aquatic systems and soils [33, 34] and has been reported to increase in zebrafish following antibiotic exposure, particularly during germ-free derivation processes [35, 36]. While *Acinetobacter* has been detected in various species of both wild and farmed fish and can transiently appear following facility water changes [13, 15, 37], it is not considered a stable “core” member of the zebrafish gut microbiome. Nor1 had abundant *Delftia*, typically found in water and soil but occasionally observed in pathogen-challenged zebrafish guts [37–40], while Nor2A was characterized by *Acidovorax* and *Limnobacter.* *Acidovorax *is commonly associated with wastewater and open water environments, and, although sometimes flagged as a potential contaminant [41], has also been documented as fish-associated and was not identified as a contaminant in our analysis [42]. *Limnobacter* has been detected in re-conventionalized zebrafish [39] and fish skin microbiomes [43] but is broadly considered environmental [44]. The context-dependent nature of these associations underscores that the boundary between fish-associated and environmentally derived taxa is not fixed, though the overall pattern suggests Norwegian facility water microbiomes are more reflective of environmental colonization than those in Oregon facilities, where taxa with stronger and more consistent fish associations dominated the water community.

One possible explanation for the striking difference between the Oregon and Norwegian facility water microbiomes is the relative age of the facilities and the total number of housed fish. The Oregon facilities have been in operation longer than those in Norway— Ore1 and Ore2 were established in 2012 and 2001, respectively, whereas Nor1 opened in 2014 and Nor2A/Nor2B in 2015. Since these facilities use recirculating aquaculture systems (RAS), the water in the Oregon facilities has been recirculating for significantly longer. Additionally, the Oregon facilities house far more fish (approximately 50,000 in Ore1 and 30,000 in Ore2) compared to those in Norway (approximately 10,000 in Nor1 and 100–200 in Nor2A and Nor2B).

While biofilters would be considered ‘mature’ after nearly a decade of operation, the longer operational period combined with higher fish populations in the Oregon facilities may have allowed for greater cumulative microbial inputs from operational factors such as feeding regimes, cleaning routines, human activity, and fish waste. Over time, these accumulated inputs may shift the microbial communities within the biofilter, water column, and biofilms throughout the facility plumbing to become increasingly reflective of the fish microbiome rather than the source water or initial environmental colonizers. Similar patterns have been observed in built environments, such as offices and homes, where the prolonged presence and activity of hosts shape the environmental microbiome through cumulative biological inputs [45, 46].

Additionally, the Norwegian facilities house more disease-model zebrafish genotypes compared to the Oregon facilities. Disease-model and immunocompromised zebrafish may have reduced host selection capacity, making them more susceptible to colonization by environmental microbes that would typically be excluded by host immune filtering. For example, genetically modified lines with mutations affecting ciliary function, such as the *foxj1b* mutant lines housed at Nor1 (Table S6), can compromise host defenses. In zebrafish, the *foxj1b* gene is essential for motile cilia formation [47], and its mammalian homolog *Foxj1* also regulates adaptive immune responses by repressing inflammatory pathways [48]. Compromised ciliary and potentially immune function may reduce the host’s ability to selectively maintain host-associated microbiota, allowing environmental microbes to colonize more readily. Consequently, the greater prevalence of pathogen- and antibiotic-associated microbiota in Norwegian facility water could be influenced not only by the higher proportion of disease-model zebrafish housed in these facilities, but also by the compromised ability of these fish to selectively maintain host-adapted microbial communities.

One hypothesis about what drives variation in microbiome composition among facilities is variation in bacterial growth rates due to differences in water exchange rates of the tanks. It has been proposed that shorter hydraulic retention times in RAS systems, which are directly tied to higher water exchange rates in tanks, promote r-selected bacterial growth whereas longer retention times promote K-selected bacterial growth [49]. Although we did not directly measure water exchange rates, most facilities used similar zebrafish husbandry equipment— Ore1, Ore2, Nor1, and Nor2A all utilized Techniplast (Buguggiate, Italy) zebrafish rearing systems. Given that variation in water exchange rates in these systems is constrained by tank design, variation among tanks within a facility is likely greater than the variation in average tank water exchange rates between facilities. Therefore, water exchange rate alone is unlikely to drive the observed differences in microbial composition among facilities.

Another more plausible driver of the observed facility-level differences is variation in tank maintenance and cleaning protocols. The Oregon facilities maintained the strictest cleaning protocols, changing tanks every two weeks or earlier if visible algal or biofilm growth was observed (T. Mason, Ore1 facility manager, personal communication, January 30, 2021; D. Lains, Ore2 facility manager, personal communication, September 21, 2022). The Norwegian Nor2A and Nor2B facilities followed a similar two-week cleaning schedule (F. Jutfelt, Nor2A/Nor2B facility manager, personal communication, January 12, 2024), whereas Nor1 changed tanks much less frequently—approximately once every two to three months (E. Yaksi, Nor1 facility manager, personal communication, June 14, 2022). All facilities used personal protective equipment to limit potential cross-contamination. We note that while cleaning frequency information was available through personal communications with facility managers, standardized cleaning records were not systematically maintained across all facilities, which precluded a more quantitative assessment of cleaning regime effects on water microbiome composition.

Despite the differences in cleaning protocols between Nor1 and Nor2A/B, nearly all sampled tanks in the Norwegian facilities exhibited at least some visible biofilm or algal growth, while the Oregon facility tanks appeared largely free of biofilms. This observation suggests that cleaning intensity, rather than frequency alone, may be a key factor driving differences in microbial composition between facilities.

The more intensive cleaning regimen in the Oregon facilities likely created distinct ecological conditions that favored fish-associated microbiota in the water column. Frequent disturbance through aggressive cleaning would reduce overall microbial biomass and remove established, competitive bacterial communities (i.e., K-selected taxa) in the tank water. This disturbance could promote the detection of fish-associated microbes through two potential mechanisms: first, fish-associated microbes dispersing from hosts could rapidly colonize the newly available niche space in the water column; alternatively, these microbes could be detected simply due to the reduced background water microbial biomass, making fish-derived taxa more prominent in the relative abundance profiles without necessarily requiring growth in the water. Either mechanism could explain the strong association between fish microbiota and Oregon water samples observed in this study.

In contrast, the greater biofilm and algal growth observed in Norwegian facility tanks may provide competitive taxa that limit the establishment of fish-associated microbes in the water column. If these biofilms help stabilize the water microbiome, this could explain why Norwegian facility water samples were more compositionally similar to one another than those from Oregon.

Ultimately, the observed differences in water microbiomes across facilities are likely shaped by multiple interacting factors, including, but not limited to, facility age, fish density, microbiota dispersal from zebrafish, tank maintenance routines, and microbial competition within the water column. The biogeographic context established by source water chemistry and regional microbial pools likely sets the baseline community composition, upon which facility-specific operational factors act as secondary structuring forces. Given these differences, it follows that zebrafish reared in different facilities experience markedly different microbial environments, which could, in turn, influence the composition of their gut microbiomes. The next section explores how these facility-level differences translate to variation in the zebrafish gut microbiome.

### Variation in the zebrafish gut microbiome

The zebrafish gut samples shared many similarities to patterns seen in the tank water samples both showed significant structuring by facility and geographic location, and both were dominated by Proteobacteria and Fusobacteriota across facilities. However, the nature of the forces shaping gut microbial communities appears to differ in their relative influence from those structuring the water microbiome. Facility-level environmental factors clearly play a role in shaping zebrafish gut microbiome composition, as evidenced by the significant effect of facility on gut community structure. Yet this variation was primarily driven by shifts among taxa already considered part of the normal zebrafish core microbiome rather than wholesale replacement by environmental colonizers, suggesting that host-level processes modulate rather than override environmental influence. Importantly, the relative strength of this host-level filtering appears to vary across facilities, a pattern we explore in more detail below.

In Roeselers et al. (2011), the authors compared the microbiomes of wild and domesticated zebrafish, finding that Proteobacteria represented over 99% of all reads in wild-caught zebrafish raised in laboratory aquaculture facilities but made up considerably smaller proportions of the microbiome of zebrafish genotypes that had been maintained for many generations in such facilities. A similar pattern was observed in our data, where Proteobacteria comprised the greatest proportion of reads in Nor2A and Nor2B upwards of 95% and 75%, respectively while making up a far lesser proportion in the other facilities. Unlike the other facilities, which maintain highly domesticated zebrafish lines, the Nor2B zebrafish are approximately sixth generation descendants of wild zebrafish collected in India in 2015, while those in Nor2A are a cross between these wild descendants and a domesticated Casper line.

This proximity to wildness may contribute to the compositional patterns observed in these facilities. Highly domesticated zebrafish lines have been maintained in laboratory aquaculture facilities for many generations, during which prolonged co-exposure between host and facility-specific microbial communities may have facilitated the adaptation of certain bacterial lineages to colonize these hosts more effectively [50]. In this context, the dominance of Fusobacteriota— particularly *Cetobacterium* in Oregon facilities housing long-domesticated lines may reflect the cumulative outcome of generations of host-microbe co-adaptation rather than just passive environmental colonization. In contrast, the more recently wild-derived zebrafish in Nor2B and Nor2A may not yet share this co-adaptive history with laboratory-associated microbial lineages, potentially explaining the greater prominence of environmental Proteobacteria in these facilities.

The domestication status of fish lines across the facilities suggests a potential relationship between host breeding history and the degree of association between gut and water microbiome composition. Facilities housing highly domesticated fish lines— Oregon facilities and Nor1, whose fish stocks were sourced from long-established laboratory lines showed moderate associations between water and fish gut microbiome composition in source tracking analyses. In contrast, Nor2B, which houses the most wild-derived fish of any facility, showed essentially complete decoupling of the fish gut microbiome from the local water environment, with unknown sources accounting for 98.9% of gut microbiome composition and same-facility water contributing only 0.5%— the most striking fish-water compositional mismatch observed across all facilities. This was further reflected in the pronounced divergence between Nor2B fish gut microbiomes dominated by *Aeromonas* (60.4%) and Nor2B tank water dominated by *Rheinheimera* (33.1%). Nor2A, housing a wild-domesticated cross, showed the strongest same-facility water contribution of any facility (57.2%), which does not fit neatly into this pattern and may reflect facility-specific microbial conditions or aspects of the hybrid genotype. Nor2B is also the only flow-through system in this study, and the reduced cumulative microbial exposure inherent to flow-through systems relative to RAS may further contribute to the decoupling observed there. If prolonged co-exposure between host lines and facility water microbiomes facilitates the establishment of certain microbial taxa over generations— as demonstrated experimentally for a *Shewanella* isolate adapted to zebrafish from Ore1 [50]— fish lines with limited co-exposure history might be expected to show weaker associations with their immediate water environment. We lack the strain-level resolution and cross-facility experimental replication necessary to directly test this hypothesis, but the consistency of the Nor2B pattern across multiple lines of evidence makes it a compelling avenue for future investigation.

Finally, effects of genotype on the composition and structure of the host microbiome have been well documented in zebrafish research [51], and the diversity of genotypes across our study facilities— from highly domesticated laboratory lines to wild-derived descendants represents both a strength and a limitation of our study design. We attempted to account for genotype by creating a binary classification of wild-type versus genetically modified lines (Table S6), but this approach necessarily obscures the substantial genetic diversity within each category. When permutations were constrained within facilities, genotype status was not significant in either fish gut or water microbiome models, indicating that we cannot distinguish a genotype effect from facility-level confounding in the current dataset. The complexity introduced by decades of cross-breeding and genetic modifications [52, 53], combined with the inherent confounding of genotype and facility in our design, means that genotype effects on microbiome composition remains an open question that future studies with dedicated cross-facility genotype replication would be better positioned to address.

### Zebrafish gut and water covariation

Lastly, to explore the covariation between fish gut and water microbiomes, we employed two complementary approaches: examining the identity of shared bacterial taxa and their known ecological associations and using source tracking to estimate the directionality of microbial exchange between the two environments. A key caveat to the first approach is that taxonomic associations can be highly context-dependent, varying by environment, host species, or experimental conditions, and our compositional sequencing approach cannot directly assess microbial function or the nature of host-microbe interactions. Inferences about directionality based on reported associations should therefore be interpreted cautiously [54].

In the Oregon facilities, water communities were dominated by taxa with strong fish associations— *Cetobacterium*, *Psychrobacter*, and *Vibrio* as discussed above, suggesting substantial microbial overlap between fish and water in these facilities. The Norwegian facilities presented a contrasting pattern, with fewer abundant water taxa also found at high abundances in the fish gut microbiome. Facility-level variation in the fish microbiomes was primarily driven by compositional shifts among microbial taxa already considered part of the normal zebrafish gut microbiome, rather than by the adoption of dominant water taxa. However, zebrafish and water did share rare ASVs unique to each facility, indicating that beyond shifts in numerically dominant taxa, facility-specific rare taxa may also contribute to the observed differences. This pattern mirrors observations in fish microbiomes across environmental gradients, where rare taxa distinguished microbial communities between conditions despite low numerical abundance, suggesting these taxa may play disproportionately important ecological roles [55].

Source tracking results further highlighted the variable nature of microbial associations between fish and water across facilities. A key finding of these analyses is that unknown sources dominated gut microbiome composition across all facilities, ranging from 71.5% in Ore1 to 98.9% in Nor2B. The majority of zebrafish gut microbiome composition cannot be attributed to tank water regardless of facility, suggesting that host-level processes, including diet, immune selection, and physiological filtering, are primary determinants of gut microbiome composition. Tank water appears to play a secondary modulating role, the strength of which varies considerably across facilities. Same-facility water contributed meaningfully in Ore1 (25.6%), Nor1 (26.7%), and Nor2A (57.2%), while contributions from the specific same-tank water sample were consistently low across all facilities (range: 0.8–9.7%), suggesting that fish gut microbiome composition reflects exposure to the broader facility water environment rather than the microbiome of any single tank at a specific point in time. This is consistent with observations in other aquatic organisms, such as common bottlenose dolphins, California sea lions, Atlantic cod, and Mediterranean gilthead sea bream, where host and environmental microbiomes share some similarities but the abundance and diversity of environmental microbiota do not necessarily correlate with variation in the host microbiome [56–58].

In the reverse analysis with water classified as the sink, same-facility fish gut microbiomes contributed moderately to water microbiome composition in Ore1 (48.4%) but less so in Norwegian facilities (Nor1: 19.9%, Nor2A: 23.1%), while Nor2B again showed minimal fish-to-water contribution (3.2%). The absence of an inverse relationship between water-to-fish and fish-to-water contributions across facilities is notable facilities showing smaller contributions of water microbiota to fish microbiomes did not correspondingly exhibit larger contributions of fish microbiota to water microbiomes. Nor2B showed low contributions in both directions, while Ore1 showed the largest fish-to-water contribution of any facility. This asymmetry suggests that the factors governing microbial exchange are not simply reciprocal, and that facility-specific conditions shape the directionality and magnitude of fish-water microbial associations in ways that are not yet fully understood.

A significant limitation of this study is that fish and water microbiomes were sampled at a single time point. Previous work [59] has shown that water microbiome composition within these facilities varies significantly over time and that this variation is correlated with changes in the fish gut microbiome. Temporal variation may therefore influence the relative association between water and gut microbiomes, and future studies incorporating repeated sampling over time would provide more accurate insight into the dynamics of these interactions. Additionally, tank maintenance schedules were not systematically recorded across all facilities, precluding a more quantitative assessment of how cleaning regime affects water microbiome composition and its downstream influence on fish gut communities.

Although we cannot fully define the mechanisms or directionality of microbial exchange between zebrafish gut and surrounding water microbiomes, our results demonstrate that both are heavily influenced by aquaculture facility and geographic location, and that they share a substantial proportion of microbial taxa. Zebrafish are widely used as model organisms in basic research, and variation in their microbiome can significantly impact the reproducibility of experimental outcomes. As noted earlier, the host-microbiome relationship mediates many aspects of host health and function, and previous studies in mice and primates have linked microbial variation to disparate experimental results associated with facility origin [7, 10, 60]. The microbiome variation we observed across facilities and regions could potentially reduce the reproducibility of zebrafish research focused on microbiome-mediated processes. Improving consistency in husbandry practices— including diet, cleaning protocols, water quality, and stocking density alongside standardized reporting of environmental microbiome conditions, could enhance reproducibility across zebrafish research facilities. Future work should aim to identify and standardize such factors, ultimately reducing microbiome variation and improving experimental consistency.

## Conclusions

Our results demonstrate that even in relatively controlled aquaculture facilities, facility-specific factors significantly shape both water and zebrafish gut microbial communities, with geographic location imposing an additional layer of structuring at broader spatial scales. While tank water microbiomes and zebrafish gut microbiomes share a substantial proportion of microbial taxa and show evidence of association, the dominant contribution of unknown sources to gut microbiome composition across all facilities suggests that host-level processes, including immune filtering, diet, and physiological selection, are primary determinants of gut microbiome composition, with the facility water environment playing a secondary modulating role. The strength of this association varied considerably across facilities and appeared related to the domestication history of the fish lines housed there, a pattern that warrants direct experimental investigation. Microbial association between zebrafish and their environment likely represents just one aspect of a broader array of facility-specific factors shaping the zebrafish gut microbiome, including husbandry practices, water quality, stocking density, and fish genotype. This study expands our understanding of the factors influencing the microbiome of zebrafish, a widely used animal model, and underscores the importance of environmental microbiome assessment as a component of experimental design in zebrafish research. We caution that researchers using zebrafish sourced from single facilities should be mindful of facility-specific microbiome variation, as unaccounted environmental microbiome differences may influence experimental outcomes and reduce cross-facility reproducibility.

## Methods

### Study sites

This study was conducted at five zebrafish aquaculture facilities spanning two geographic regions: three located on the University of Oregon campus in Eugene, Oregon, USA, and two located at the Norwegian University of Science and Technology (NTNU) in Trondheim, Norway. These facilities were chosen because they provide a unique system to investigate host-environment microbial interactions across spatial scales and facility designs. Specifically, their close clustering within each geographic region allows investigation of variation in microbiome composition both within and between facilities in close proximity, while the inclusion of facilities across continents introduces larger-scale geographic comparison. Additionally, the sites encompass a range of rearing approaches, genotypic diversity, and life support systems representative of common practices in modern aquaculture, making the results broadly applicable to other research settings.

Both U.S. facilities included in this study are housed on the main University of Oregon campus. The first, the Aquatic Animal Care Services Zebrafish Facility (Ore1), was established in 2012 and originally stocked with fish from commercial suppliers. It spans approximately 1,000 square meters with an average capacity of 50,000 fish (maximum capacity ~ 88,000) representing around 2,900 genotypic lines. Fish are housed in 3.5-liter acrylic tanks connected to two closed-loop recirculating aquaculture systems (RAS) that utilize automated mechanical and biological filtration and ultraviolet (UV) disinfection. There is also a separate quarantine area with a dedicated flow-through system.

The second U.S. facility, the Zebrafish International Resource Center (Ore2), was built in 1999 and began operation in 2001. Ore2 covers 900 square meters and maintains an average of 30,000 fish (maximum capacity ~ 150,000), representing approximately 45 live genotypic lines and over 44,000 genotypes stored in cryogenic preservation. The layout and life support systems in Ore2 are comparable to those in Ore1, with fish housed in both 3.5-liter acrylic and 75-liter glass tanks. This facility is located approximately 300 m from Ore1, enabling comparisons between adjacent facilities that share a similar local environment but operate independently.

The three Norwegian zebrafish facilities are located on the NTNU Øya and Gløshaugen campuses in Trondheim, Norway. The Yaksi Lab facility (Nor1) is situated on the Øya campus and was built in 2014 for research on brain function and neurological disease. The facility is 70 square meters in size and houses approximately 10,000 zebrafish representing over 100 genotypic lines. Fish are kept in 3.5-liter acrylic tanks attached to a RAS with UV-treatment. There is also a separate quarantine area using a flow-through system with roughly 50 tanks.

The Jutfelt Lab facility (Nor2) is located on the Gløshaugen campus and was originally established as a general animal facility in the 1990s before being repurposed to house fish between 2015 and 2020. The lab maintains both zebrafish and guppies (*Poecilia reticulata*) in five separate rooms (each approximately 10–30 square meters) with independent life support systems. While administratively considered one facility, we reference two zebrafish-only rooms within the Jutfelt Lab as Nor2A and Nor2B due to their distinct system configurations.

Nor2A contains approximately 50, 3.5-liter acrylic tanks on a standalone RAS with UV-treatment. Nor2B houses 14, 75-liter glass tanks connected to a flow-through filtration system with no UV-treatment. Together, the Nor2 rooms house roughly 2,000 fish. Unlike the other facilities, which maintain common laboratory zebrafish lines, the Jutfelt Lab exclusively houses zebrafish descended from wild-caught individuals collected in India in 2016 (~ 6–10 generations removed; Nor2B) and wild-Casper hybrid lines (Nor2A).

### Sample collection and processing

#### Tank water

We collected one tank water sample per tank from a representative number of tanks within each facility, targeting tanks connected to a single RAS system and housing adult zebrafish. We adjusted the number of tanks sampled based on the size and housing configuration of each facility (n = Ore1 (22 tanks); Ore2 (12 tanks); Nor1 (7 tanks); Nor2A (6 tanks); Nor2B (3 tanks)). For each RAS system, we chose one to two adjacent shelf sets, then randomly selected three shelves from each set. On each selected shelf, we randomly sampled three tanks. When fewer than three shelves or tanks were available (e.g., Nor2B, which had two shelves with two tanks each), we sampled all available tanks.

To collect microbial biomass, we passed 150 mL of tank water through a 0.2 μm Sterivex filter cartridge (MilliporeSigma, Massachusetts, USA) using a 60-mL syringe. We capped the Sterivex filters and kept them on ice until processing. If we could not perform DNA extraction immediately, we stored the dry filter cartridges at − 80 °C.

#### Zebrafish

We collected zebrafish from the same tanks used for water sampling (n = Ore1 (55); Ore2 (5); Nor1 (36); Nor2A (23); Nor2B (15)). We euthanized fish via rapid chilling and sampled four individuals per tank. If fewer than four fish were available or sampling was limited, we collected as many individuals as possible.

On the same day, we aseptically dissected the whole intestine from each fish and transferred it into a 2-mL RHINO screw-cap tube containing zirconium oxide beads and 400 µL enzymatic lysis buffer (20 mM Tris-Cl, pH 8.0; 2 mM sodium EDTA; 1.2% Triton X-100). If immediate DNA extraction was not possible, we stored the tubes at − 80 °C.

### Microbial DNA extraction

#### Tank water

We performed DNA extraction using the DNeasy PowerWater Sterivex kit (QIAGEN, Carlsbad, California, USA). DNA was extracted following the standard protocol provided in the kit handbook (2009, pg. 9).

#### Zebrafish

We performed DNA extraction from zebrafish gut samples using an adapted protocol based on the DNeasy Blood & Tissue Kit Quick-Start protocol, as described in Stephens et al. (2015).

### 16S rRNA gene amplification, library preparation, and sequencing

DNA extraction products were quantified using a Qubit dsDNA HS Assay Kit (Thermo Fisher Scientific, Waltham, MA, USA). To amplify the V4 region of the 16S rRNA gene, dual-indexed primers (515 F–806R) were used, with unique 8-bp index sequences incorporated as described in Caporaso et al. (2011): 515 F (AATGATACGGCGACCACCGAGATCTACAC[index]TATGGTAATTGTGTGCCAGCMGCCGCGGTAA) and 806R (CAAGCAGAAGACGGCATACGAGAT[index]AGTCAGTCAGCCGGACTACHVGGGTWTCTAAT).

PCR reactions included 12.5 µL NEBNext Q5 Hot Start HiFi PCR Master Mix (New England Biolabs, Ipswich, MA, USA), 10.5 µL DNA template, 1 µL Bovine Serum Albumin (Thermo Fisher Scientific), and 1 µL of mixed primers (12.5 mM). Cycling conditions were: initial denaturation at 98 °C for 30 s; 30 cycles of 98 °C for 10 s, 61 °C for 20 s, and 72 °C for 20 s; and a final extension at 72 °C for 2 min. Samples were held at 4 °C until removal from the thermocycler.

To confirm amplification success, PCR products were visualized on an agarose gel. Amplicon libraries were cleaned twice using Mag-Bind RxnPure Plus isolation beads with a modified 0.8× reaction volume protocol (Omega Bio-Tek, Norcross, GA, USA). Concentrations were then quantified using the Quant-iT 1× dsDNA HS Assay Kit (Thermo Fisher Scientific) and a SpectraMax M5E Microplate Reader (Molecular Devices, San Jose, CA, USA). Libraries were pooled at equimolar concentrations and sequenced at the University of Oregon Genomics & Cell Characterization Core Facility on an Illumina MiSeq (Illumina, San Diego, CA, USA) with 150-bp paired-end reads.

### Bioinformatics

We performed all bioinformatics processing in ‘R’ [61]. We demultiplexed and then denoised the sequences to construct an amplicon sequence variant (ASV) table using DADA2 1.16 [62]. We assigned taxonomy to sequences using the RDP Classifier and Silva NR99 v.138.1 16S rRNA gene reference database [63, 64]. We evaluated sample contaminants using the prevalence method with ‘decontam’, which identifies contaminants based on their frequency of occurrence in negative control samples relative to true samples [65]. This analysis identified three ASVs as contaminants: ASV 10 (*Pseudomonas stutzeri*), ASV 29 (*Hyphomonas*), and ASV 466 (*Janthinobacterium*) which were subsequently removed from all downstream analyses.

A phylogenetic tree was constructed from ASV sequences for use in unweighted UniFrac distance calculations. ASV sequences were aligned using the MUSCLE algorithm [66] implemented in the ‘msa’ package [67]. An initial neighbor-joining tree was constructed from maximum likelihood distances using the ‘phangorn’ package [68], then optimized using maximum likelihood estimation with a GTR + Γ + I substitution model with stochastic rearrangements. The resulting maximum likelihood tree was rooted prior to incorporation into downstream phylogenetic analyses.

### Statistics

We conducted all statistical analyses in ‘R’. Alpha diversity metrics (Shannon and Inverse Simpson) were calculated using the ‘phyloseq’ package [69]. Beta diversity was assessed using Bray-Curtis dissimilarity and unweighted UniFrac distances, calculated using the ‘vegan’ package [70, 71]. All plots were created with the ‘ggplot2’ package [72].

Prior to analysis, samples with fewer than 1,000 reads were removed as low-quality samples unlikely to be representative of true community composition resulting in a final dataset of 50 water samples (Ore1: *n* = 22; Ore2: *n* = 12; Nor1: *n* = 7; Nor2A: *n* = 6; Nor2B: *n* = 3) and 134 fish samples (Ore1: *n* = 55; Ore2: *n* = 5; Nor1: *n* = 36; Nor2A: *n* = 23; Nor2B: *n* = 15). Fish gut and water samples were rarefied separately to their respective minimum read counts after quality filtering— 1,171 reads for fish gut samples and 1,291 reads for water samples for all single sample type analyses. For combined fish gut and water microbiome analyses, all samples were rarefied to a common threshold of 1,171 reads to ensure equivalent sequencing depth across sample types. Results of combined analyses were robust to the choice of rarefaction threshold, with negligible differences observed between thresholds. Mean sequence reads per sample prior to rarefaction were 16,624 ± 10,171 (SD) for water samples and 22,047 ± 27,137 (SD) for fish samples.

To test for differences in alpha diversity in water samples across facilities, we applied non-parametric Kruskal-Wallis tests [73], followed by pairwise Wilcoxon rank-sum tests with Benjamini-Hochberg p-value correction [74, 75]. For fish gut samples, alpha diversity was assessed using linear mixed models (LMM) with facility as a fixed effect and tank as a random effect to account for non-independence of fish within tanks, implemented using the ‘lme4’ and ‘lmerTest’ packages [76, 77]. Overall significance was assessed using Type III ANOVA with Satterthwaite’s method, and post-hoc pairwise comparisons of estimated marginal means were performed using the ‘emmeans’ package with Benjamini-Hochberg correction [78].

To assess the effects of geographic location, facility, and genotype status on microbial community dissimilarity, we performed permutational multivariate analysis of variance (PERMANOVA) using the ‘adonis2’ function in the ‘vegan’ package with 9,999 permutations [71]. We used a hierarchical model formula to account for the nested structure of the data, where facilities are nested within geographic locations (Norway vs. Oregon). To determine whether genotype status independently influenced microbiome composition after accounting for facility-level differences, we performed a constrained PERMANOVA with permutations restricted within each facility using the ‘how()’ function from the ‘permute’ package [79]. Multivariate homogeneity of group dispersions was assessed using the ‘betadisper’ function in ‘vegan’, with significance tested using ‘permutest’ with 9,999 permutations. For fish samples, betadisper permutations were stratified by tank using the ‘how()’ function from the ‘permute’ package to account for non-independence of fish within tanks. Pairwise dispersion comparisons were conducted using the permutation-based pairwise test within ‘permutest’.

For fish beta diversity analyses, individual fish samples were aggregated to tank-level mean community profiles prior to distance matrix calculation, as multiple fish were collected from each tank. This aggregation was performed after rarefaction to preserve the integrity of count-based normalization. Tank-level aggregation resolves the non-independence of fish within tanks and ensures that each tank contributes equally to facility-level analyses regardless of the number of fish sampled. Pairwise PERMANOVA tests were performed using the ‘pairwiseAdonis’ package with Benjamini-Hochberg correction and 9,999 permutations [80]. Principal coordinate analysis (PCoA) was used to visualize microbiome composition clustering. For combined fish gut and water microbiome analyses, only Bray-Curtis dissimilarity was used, as calculation of UniFrac distances requires a shared phylogenetic tree. Merging fish and water phyloseq objects resulted in differing tip counts between sample types, precluding the use of a shared tree without substantial reduction in taxa representation.

Bray-Curtis dissimilarity and unweighted UniFrac distances were chosen as complementary metrics; Bray-Curtis captures abundance-weighted compositional differences, while unweighted UniFrac incorporates phylogenetic relatedness and captures presence/absence variation, which was of particular interest given observed taxonomic overlap between geographically distinct lineages. Weighted UniFrac was not included to limit the number of distance metrics and maintain analytical focus.

To generate relative abundance summaries, ASVs were agglomerated at the genus level. For fish gut samples, relative abundances were calculated after aggregation to tank-level mean profiles, then averaged to the facility level to ensure equal weighting of tanks regardless of fish sample size. For water samples, relative abundances were averaged directly to the facility level. Genera exceeding 1% mean relative abundance across all facilities were identified for each sample type separately and in combination. Relative abundance visualizations were created using the ‘fantaxtic’ package and based on individual samples [81].

To identify genera contributing most to compositional differences between facilities, we performed Similarity Percentage (SIMPER) analysis using Bray-Curtis dissimilarity within the ‘vegan’ package. For fish gut samples, SIMPER was performed on tank-level aggregated community profiles for consistency with other beta diversity analyses. Benjamini-Hochberg correction was applied within each pairwise facility comparison to account for multiple taxa being tested simultaneously. Results are reported for the top significantly contributing genera per comparison.

For pairwise beta diversity comparisons between fish gut and tank water microbiomes, Bray-Curtis dissimilarity values were categorized hierarchically into three groups: (1) fish gut and water samples from the same tank, (2) fish gut and water samples from the same facility but different tanks, and (3) fish gut and water samples from different facilities. This hierarchical classification ensured that same-tank pairs were not included in the same-facility category. Tank-averaged fish community profiles were used for all comparisons. Due to inherent imbalance in the number of pairwise comparisons available across categories and non-independence of samples within facilities, results are presented descriptively without formal significance testing. Ore2 was excluded from this analysis due to single-tank fish representation (*n* = 1 tank) precluding meaningful within-facility comparisons.

FEAST analyses were conducted at the ASV-level within each facility separately, as fish are not exposed to water from other facilities [20]. Sources were defined as tank water samples from within the same facility as the sink fish gut samples, and the unknown fraction represents microbial sources not captured by facility tank water, including potential contributions from diet, host physiology, or other facility-specific environmental factors. Individual fish gut samples were used as sinks, as source tracking estimates the contribution of environmental sources to individual community compositions rather than testing group-level differences. Source contributions were categorized as same-tank (water from the fish’s own tank), same-facility (water from other tanks within the same facility), or unknown. Because microbial exchange can be bidirectional, we also performed the reverse analysis treating water samples as sinks and fish gut samples as sources. Results are presented descriptively as the compositional nature of source proportions and non-independence of samples within facilities precluded formal significance testing. Ore2 was excluded from the water-as-sink analysis as only one of twelve water samples had a corresponding fish gut sample from the same tank, precluding meaningful facility-level interpretation.

All fish-based analyses were repeated excluding the Ore2 facility (*n* = 5 fish, single tank) as sensitivity analyses, given the absence of within-facility tank replication and the small sample size. Sensitivity analysis results are provided in Supplementary Material 5 and conclusions are noted in the Results if they differed from the primary analyses.

## Electronic Supplementary Material

Below is the link to the electronic supplementary material.

Supplementary Material 1: Supplementary Table S1, Table S2, Table S3, Table S4, Table S5, Figure S1, and Table S6. Table S1 contains pairwise estimated marginal means comparisons for zebrafish gut alpha diversity. Table S2 contains constrained PERMANOVA results assessing the effect of fish genotype status on tank water microbiome composition after accounting for facility-level differences. Table S3 contains the top ten significantly contributing taxa identified by SIMPER analysis for tank water microbiome facility comparisons. Table S4 contains constrained PERMANOVA results assessing the effect of fish genotype status on zebrafish gut microbiome composition after accounting for facility-level differences. Table S5 contains the top ten significantly contributing taxa identified by SIMPER analysis for zebrafish gut microbiome facility comparisons. Figure S1 contains Bray-Curtis dissimilarity boxplots of paired fish gut and tank water samples separated by facility. Table S6 includes zebrafish genotypic line information for all facilities

Supplementary Material 2: Statistical analyses for tank water and zebrafish gut microbiomes across aquaculture facilities (R Script). R Markdown script including all code used to run the statistical analyses and produce the figures used in the Evens et al. manuscript

Supplementary Material 3: Statistical analyses for tank water and zebrafish gut microbiomes across aquaculture facilities (R Markdown Document). html ouput file of complete R Markdown script

Supplementary Material 4: Evens et al. phyloseq object. Phyloseq object (containing ASV, taxonomy, phylogentic tree, and metadata files) used for R Markdown script containing all statistical analyses.

Supplementary Material 5: Exclusion-based sensitivity analyses for Ore2 fish gut microbiome samples

## Data Availability

The datasets generated and analysed during the current study are available in the NCBI Sequence Read Archive, Accession Number PRJNA1345588. https://www.ncbi.nlm.nih.gov/bioproject/PRJNA1345588.

## Abbreviations

ASV: Amplicon Sequence Variant; high-resolution DNA sequences used to identify microbial taxa
BSA: Bovine Serum Albumin; a protein added to PCR reactions to enhance performance
FEAST: Fast Expectation-Maximization for Microbial Source Tracking; a tool for estimating microbial source contributions
PCoA: Principal Coordinate Analysis; a method for visualizing differences in microbial communities
PCR: Polymerase Chain Reaction; a technique used to amplify DNA
PERMANOVA: Permutational Multivariate Analysis of Variance; a statistical test for group differences based on distance metrics
RAS: Recirculating Aquaculture System; a closed-loop water filtration system used in aquaculture
SIMPER: Similarity Percentage Analysis; a method to determine which taxa contribute most to differences between groups
