# Supplementary material for: Aquaculture facility-specific microbiota shape the zebrafish gut microbiome
Source: Anim Microbiome. 2026 May 7;8:84. doi: 10.1186/s42523-026-00573-6 (PMC13321582; doi:10.1186/s42523-026-00573-6)
Supplement: Supplementary file 1 — Supplementary Material 1: Supplementary Table S1, Table S2, Table S3, Table S4, Table S5, Figure S1, and Table S6. Table S1 contains pairwise estimated marginal means comparisons for zebrafish gut alpha diversity. Table S2 contains constrained PERMANOVA results assessing the effect of fish genotype status on tank water microbiome composition after accounting for facility-level differences. Table S3 contains the top ten significantly contributing taxa identified by SIMPER analysis for tank water microbiome facility comparisons. Table S4 contains constrained PERMANOVA results assessing the effect of fish genotype status on zebrafish gut microbiome composition after accounting for facility-level differences. Table S5 contains the top ten significantly contributing taxa identified by SIMPER analysis for zebrafish gut microbiome facility comparisons. Figure S1 contains Bray-Curtis dissimilarity boxplots of paired fish gut and tank water samples separated by facility. Table S6 includes zebrafish genotypic line information for all facilities [file 42523_2026_573_MOESM1_ESM.docx]

Supplementary Materials 1

**Table S1:** Pairwise comparisons of estimated marginal means for zebrafish gut microbiota alpha diversity across facilities. Estimated marginal means (emmeans) and pairwise contrasts are presented for (A) Shannon diversity and (B) Inverse Simpson diversity, derived from linear mixed models with tank included as a random effect. Emmeans represent model-estimated group means marginal to tank-level clustering, with 95% confidence intervals calculated using the Kenward-Roger degrees of freedom method. Pairwise contrasts present the difference in estimated marginal means between facility pairs, with p-values adjusted for multiple comparisons using the Benjamini-Hochberg method. No pairwise contrasts reached significance for Inverse Simpson diversity following p-value correction.

| **A. Shannon diversity index (H)** | | | | | | | | |
| --- | --- | --- | --- | --- | --- | --- | --- | --- |
| Estimated Marginal Means | | | | | | | | |
| Facility | emmean | SE | df | lower.CL | upper.CL | estimate | t.ratio | p.value (adj.) |
| Nor1 | 1.709 | 0.176 | 27.82 | 1.348 | 2.069 |  |  |  |
| Nor2A | 0.844 | 0.203 | 39.44 | 0.434 | 1.255 |  |  |  |
| Nor2B | 1.964 | 0.266 | 29.60 | 1.420 | 2.507 |  |  |  |
| Ore1 | 1.863 | 0.129 | 43.03 | 1.602 | 2.124 |  |  |  |
| Ore2 | 1.865 | 0.495 | 22.16 | 0.838 | 2.891 |  |  |  |
| Pairwise Contrasts | | | | | | | | |
| Nor1 - Nor2A |  | 0.269 | 33.80 |  |  | 0.864 | 3.218 | **0.009** |
| Nor1 - Nor2B |  | 0.319 | 29.04 |  |  | -0.255 | -0.801 | 0.808 |
| Nor1 - Ore1 |  | 0.218 | 32.20 |  |  | -0.154 | -0.707 | 0.808 |
| Nor1 - Ore2 |  | 0.525 | 22.71 |  |  | -0.156 | -0.297 | 0.957 |
| Nor2A - Nor2B |  | 0.335 | 32.80 |  |  | -1.120 | -3.347 | **0.009** |
| Nor2A - Ore1 |  | 0.241 | 40.44 |  |  | -1.019 | -4.231 | **0.001** |
| Nor2A - Ore2 |  | 0.535 | 23.90 |  |  | -1.020 | -1.907 | 0.172 |
| Nor2B - Ore1 |  | 0.296 | 31.69 |  |  | 0.101 | 0.341 | 0.957 |
| Nor2B - Ore2 |  | 0.562 | 23.58 |  |  | 0.099 | 0.176 | 0.957 |
| Ore1 - Ore2 |  | 0.512 | 23.01 |  |  | -0.002 | -0.004 | 0.997 |
|  |  |  |  |  |  |  |  |  |
| **B. Inverse Simpson index (1-λ)** | | | | | | | | |
| Estimated Marginal Means | | | | | | | | |
| Nor1 | 4.889 | 0.827 | 24.54 | 3.185 | 6.593 |  |  |  |
| Nor2A | 1.978 | 0.984 | 40.36 | -0.011 | 3.967 |  |  |  |
| Nor2B | 3.429 | 1.256 | 27.79 | 0.854 | 6.003 |  |  |  |
| Ore1 | 4.506 | 0.631 | 45.01 | 3.235 | 5.777 |  |  |  |
| Ore2 | 3.649 | 2.271 | 18.42 | -1.114 | 8.412 |  |  |  |
| Nor1 | 4.889 | 0.827 | 24.54 | 3.185 | 6.593 |  |  |  |
| Nor2A | 1.978 | 0.984 | 40.36 | -0.011 | 3.967 |  |  |  |
| Pairwise Contrasts | | | | | | | | |
| Nor1 - Nor2A |  | 1.286 | 32.54 |  |  | 2.911 | 2.265 | 0.182 |
| Nor1 - Nor2B |  | 1.504 | 26.75 |  |  | 1.460 | 0.971 | 0.800 |
| Nor1 - Ore1 |  | 1.040 | 30.28 |  |  | 0.383 | 0.368 | 0.800 |
| Nor1 - Ore2 |  | 2.417 | 19.02 |  |  | 1.240 | 0.513 | 0.800 |
| Nor2A - Nor2B |  | 1.596 | 31.85 |  |  | -1.451 | -0.909 | 0.800 |
| Nor2A - Ore1 |  | 1.169 | 41.65 |  |  | -2.528 | -2.162 | 0.182 |
| Nor2A - Ore2 |  | 2.475 | 20.55 |  |  | -1.671 | -0.675 | 0.800 |
| Nor2B - Ore1 |  | 1.406 | 30.44 |  |  | -1.077 | -0.766 | 0.800 |
| Nor2B - Ore2 |  | 2.595 | 20.17 |  |  | -0.221 | -0.085 | 0.933 |
| Ore1 - Ore2 |  | 2.357 | 19.46 |  |  | 0.857 | 0.363 | 0.800 |

**Table S2:** PERMANOVA results for water microbiota comparisons between zebrafish facilities. Statistical significance was assessed using Bray-Curtis dissimilarity and unweighted UniFrac distance metrics under both hierarchical and constrained models. Significant differences (*p* < 0.05) are indicated.

| **Bray-Curtis: Hierarchical** | | | | | |
| --- | --- | --- | --- | --- | --- |
|  | Df | SumOfSqs | R2 | F | Pr(>F) |
| Location | 1 | 3.538 | 0.204 | 17.141 | < 0.001 |
| Geno_Status | 1 | 0.735 | 0.042 | 3.560 | 0.001 |
| Location:Facility | 3 | 3.966 | 0.229 | 6.406 | < 0.001 |
| Residual | 44 | 9.080 | 0.524 | NA | NA |
| Total | 49 | 17.319 | 1.000 | NA | NA |
|  |  |  |  |  |  |
| **Bray-Curtis: Constrained** | | | | | |
| Geno_Status | 1 | 2.724 | 0.157 | 8.958 | 0.429 |
| Residual | 48 | 14.595 | 0.843 | NA | NA |
| Total | 49 | 17.319 | 1 | NA | NA |
|  |  |  |  |  |  |
| **Unweighted UniFrac: Hierarchical** | | | | | |
| Location | 1 | 1.659 | 0.133 | 9.027 | < 0.001 |
| Geno_Status | 1 | 0.501 | 0.040 | 2.724 | 0.001 |
| Location:Facility | 3 | 2.268 | 0.181 | 4.112 | < 0.001 |
| Residual | 44 | 8.088 | 0.646 | NA | NA |
| Total | 49 | 12.516 | 1.000 | NA | NA |
|  |  |  |  |  |  |
| **Unweighted UniFrac: Constrained** | | | | | |
| Geno_Status | 1 | 1.471 | 0.118 | 6.392 | 0.288 |
| Residual | 48 | 11.045 | 0.882 | NA | NA |
| Total | 49 | 12.516 | 1.000 | NA | NA |

**Table S3:** Top 10 most significant ASVs contributing to water microbiome dissimilarity between facility comparisons (SIMPER analysis, p-adj. < 0.05).

| **Facility Comparison** | **Genus** | **Average Contribution (%)** | **p-adj.** | **Rank** |
| --- | --- | --- | --- | --- |
| **Ore1 vs. Ore2** | | | | |
| Ore1 vs Ore2 | Cetobacterium | 14.2 | 0.040 | 1 |
| Ore1 vs Ore2 | Vibrio | 4.58 | 0.002 | 4 |
| Ore1 vs Ore2 | Plesiomonas | 2.38 | 0.002 | 8 |
| Ore1 vs Ore2 | Chitinibacter | 1.37 | 0.002 | 10 |
| Ore1 vs Ore2 | Chitinimonas | 1.01 | 0.002 | 12 |
| Ore1 vs Ore2 | Crenobacter | 0.63 | 0.002 | 15 |
| Ore1 vs Ore2 | Haliangium | 0.49 | 0.002 | 19 |
| Ore1 vs Ore2 | Epulopiscium | 0.45 | 0.002 | 21 |
| Ore1 vs Ore2 | Rhizorhapis | 0.36 | 0.050 | 24 |
| Ore1 vs Ore2 | Romboutsia | 0.33 | 0.002 | 28 |
| **Ore1 vs. Nor1** | | | | |
| Ore1 vs Nor1 | Pseudomonas | 17.72 | 0.004 | 1 |
| Ore1 vs Nor1 | Massilia | 1.84 | 0.004 | 12 |
| Ore1 vs Nor1 | Variovorax | 1 | 0.004 | 16 |
| Ore1 vs Nor1 | Deinococcus | 0.58 | 0.021 | 22 |
| Ore1 vs Nor1 | Ottowia | 0.58 | 0.021 | 23 |
| Ore1 vs Nor1 | Alkanibacter | 0.48 | 0.004 | 26 |
| Ore1 vs Nor1 | Sphingobacterium | 0.46 | 0.004 | 27 |
| Ore1 vs Nor1 | Comamonas | 0.28 | 0.004 | 37 |
| Ore1 vs Nor1 | Bordetella | 0.25 | 0.036 | 38 |
| Ore1 vs Nor1 | Mesorhizobium | 0.14 | 0.004 | 52 |
| **Ore1 vs. Nor2A** | | | | |
| Ore1 vs Nor2A | Acidovorax | 5.69 | 0.007 | 4 |
| Ore1 vs Nor2A | Limnobacter | 4.3 | 0.025 | 6 |
| Ore1 vs Nor2A | Luteimonas | 3.01 | 0.007 | 11 |
| Ore1 vs Nor2A | Brevundimonas | 2.17 | 0.007 | 15 |
| Ore1 vs Nor2A | Pelomonas | 2.02 | 0.030 | 17 |
| Ore1 vs Nor2A | Rhodoferax | 1.06 | 0.007 | 22 |
| Ore1 vs Nor2A | Pseudoxanthomonas | 0.4 | 0.011 | 26 |
| Ore1 vs Nor2A | Sphingopyxis | 0.3 | 0.029 | 29 |
| Ore1 vs Nor2A | Caulobacter | 0.28 | 0.007 | 33 |
| Ore1 vs Nor2A | Dyadobacter | 0.27 | 0.032 | 34 |
| **Ore1 vs. Nor2B** | | | | |
| Ore1 vs Nor2B | Rheinheimera | 16.98 | 0.005 | 1 |
| Ore1 vs Nor2B | Fluviicola | 5.54 | 0.005 | 5 |
| Ore1 vs Nor2B | Polynucleobacter | 1.48 | 0.032 | 12 |
| Ore1 vs Nor2B | Sediminibacterium | 1.31 | 0.005 | 13 |
| Ore1 vs Nor2B | hgcI clade | 1.24 | 0.024 | 14 |
| Ore1 vs Nor2B | Candidatus Nitrosotenuis | 1.1 | 0.005 | 15 |
| Ore1 vs Nor2B | Candidatus Omnitrophus | 0.34 | 0.032 | 28 |
| Ore1 vs Nor2B | Cellvibrio | 0.29 | 0.014 | 33 |
| Ore1 vs Nor2B | Xanthobacter | 0.14 | 0.005 | 43 |
| Ore1 vs Nor2B | Sulfurifustis | 0.09 | 0.032 | 55 |
| **Ore2 vs. Nor1** | | | | |
| Ore2 vs Nor1 | Cetobacterium | 18.45 | 0.009 | 1 |
| Ore2 vs Nor1 | Pseudomonas | 18.07 | 0.004 | 2 |
| Ore2 vs Nor1 | Vibrio | 4.23 | 0.048 | 3 |
| Ore2 vs Nor1 | Plesiomonas | 2.62 | 0.013 | 6 |
| Ore2 vs Nor1 | Massilia | 1.87 | 0.004 | 9 |
| Ore2 vs Nor1 | Variovorax | 1.03 | 0.004 | 15 |
| Ore2 vs Nor1 | Nubsella | 0.95 | 0.040 | 17 |
| Ore2 vs Nor1 | Crenobacter | 0.64 | 0.017 | 20 |
| Ore2 vs Nor1 | Deinococcus | 0.6 | 0.014 | 21 |
| Ore2 vs Nor1 | Ottowia | 0.6 | 0.017 | 22 |
| **Ore2 vs. Nor2A** | | | | |
| Ore2 vs Nor2A | Cetobacterium | 20.87 | 0.007 | 1 |
| Ore2 vs Nor2A | Acidovorax | 5.7 | 0.005 | 2 |
| Ore2 vs Nor2A | Nevskia | 5.28 | 0.044 | 3 |
| Ore2 vs Nor2A | Limnobacter | 4.57 | 0.005 | 4 |
| Ore2 vs Nor2A | Plesiomonas | 3.12 | 0.005 | 9 |
| Ore2 vs Nor2A | Luteimonas | 3.06 | 0.005 | 10 |
| Ore2 vs Nor2A | Brevundimonas | 2.21 | 0.005 | 13 |
| Ore2 vs Nor2A | Methyloversatilis | 1.38 | 0.048 | 17 |
| Ore2 vs Nor2A | Rhodoferax | 1.07 | 0.005 | 21 |
| Ore2 vs Nor2A | Crenobacter | 0.67 | 0.022 | 25 |
| **Ore2 vs. Nor2B** | | | | |
| Ore2 vs Nor2B | Rheinheimera | 17.18 | 0.005 | 2 |
| Ore2 vs Nor2B | Fluviicola | 5.53 | 0.005 | 4 |
| Ore2 vs Nor2B | Polynucleobacter | 1.49 | 0.038 | 11 |
| Ore2 vs Nor2B | Sediminibacterium | 1.33 | 0.005 | 14 |
| Ore2 vs Nor2B | hgcI clade | 1.25 | 0.023 | 15 |
| Ore2 vs Nor2B | Candidatus Nitrosotenuis | 1.11 | 0.005 | 17 |
| Ore2 vs Nor2B | Candidatus Omnitrophus | 0.35 | 0.018 | 28 |
| Ore2 vs Nor2B | Cellvibrio | 0.28 | 0.018 | 30 |
| Ore2 vs Nor2B | Xanthobacter | 0.14 | 0.005 | 42 |
| Ore2 vs Nor2B | Sulfurifustis | 0.09 | 0.018 | 50 |
| **Nor2A vs. Nor1** | | | | |
| Nor2A vs Nor1 | Pseudomonas | 14.86 | 0.005 | 1 |
| Nor2A vs Nor1 | Acidovorax | 5.43 | 0.005 | 2 |
| Nor2A vs Nor1 | Delftia | 5.09 | 0.021 | 4 |
| Nor2A vs Nor1 | Limnobacter | 4.34 | 0.016 | 5 |
| Nor2A vs Nor1 | Luteimonas | 2.73 | 0.005 | 8 |
| Nor2A vs Nor1 | Brevundimonas | 2.03 | 0.005 | 11 |
| Nor2A vs Nor1 | Massilia | 1.81 | 0.005 | 13 |
| Nor2A vs Nor1 | Methyloversatilis | 1.45 | 0.032 | 16 |
| Nor2A vs Nor1 | Rhodoferax | 1.03 | 0.016 | 20 |
| Nor2A vs Nor1 | Variovorax | 1.01 | 0.005 | 21 |
| **Nor2B vs. Nor1** | | | | |
| Nor2B vs Nor1 | Pseudomonas | 17.23 | 0.046 | 1 |
| Nor2B vs Nor1 | Rheinheimera | 16.43 | 0.016 | 2 |
| Nor2B vs Nor1 | Fluviicola | 5.36 | 0.016 | 4 |
| Nor2B vs Nor1 | Sediminibacterium | 1.2 | 0.046 | 14 |
| Nor2B vs Nor1 | Candidatus Omnitrophus | 0.33 | 0.047 | 34 |
| Nor2B vs Nor1 | Cellvibrio | 0.28 | 0.045 | 38 |
| Nor2B vs Nor1 | Xanthobacter | 0.14 | 0.016 | 53 |
| Nor2B vs Nor1 | Sulfurifustis | 0.09 | 0.047 | 67 |
| Nor2B vs Nor1 | HdN1 | 0.06 | 0.016 | 83 |
| **Nor2A vs. Nor2B** | | | | |
| Nor2A vs Nor2B | Rheinheimera | 13.35 | 0.024 | 1 |
| Nor2A vs Nor2B | Fluviicola | 5.44 | 0.011 | 3 |
| Nor2A vs Nor2B | Candidatus Nitrosotenuis | 1.08 | 0.032 | 21 |
| Nor2A vs Nor2B | Candidatus Omnitrophus | 0.34 | 0.045 | 30 |
| Nor2A vs Nor2B | Cellvibrio | 0.29 | 0.026 | 33 |
| Nor2A vs Nor2B | Xanthobacter | 0.14 | 0.011 | 43 |
| Nor2A vs Nor2B | Sulfurifustis | 0.09 | 0.045 | 50 |
| Nor2A vs Nor2B | HdN1 | 0.06 | 0.011 | 53 |

**Table S4:** PERMANOVA results for fish microbiota comparisons between zebrafish facilities. Statistical significance was assessed using Bray-Curtis dissimilarity and unweighted UniFrac distance metrics under both hierarchical and constrained models. Significant differences (p < 0.05) are indicated.

| **Bray-Curtis: Hierarchical** | | | | | |
| --- | --- | --- | --- | --- | --- |
|  | Df | SumOfSqs | R2 | F | Pr(>F) |
| Location | 1 | 3.782 | 0.323 | 25.655 | < 0.001 |
| Geno_Status | 1 | 0.530 | 0.045 | 3.594 | 0.006 |
| Location:Facility | 3 | 1.934 | 0.165 | 4.373 | < 0.001 |
| Residual | 37 | 5.455 | 0.466 | NA | NA |
| Total | 42 | 11.700 | 1 | NA | NA |
|  |  |  |  |  |  |
| **Bray-Curtis: Constrained** | | | | | |
|  | Df | SumOfSqs | R2 | F | Pr(>F) |
| Geno_Status | 1 | 2.380 | 0.203 | 10.468 | 0.376 |
| Residual | 41 | 9.321 | 0.797 | NA | NA |
| Total | 42 | 11.700 | 1 | NA | NA |
|  |  |  |  |  |  |
| **Unweighted UniFrac: Hierarchical** | | | | | |
|  | Df | SumOfSqs | R2 | F | Pr(>F) |
| Location | 1 | 2.188 | 0.201 | 12.606 | < 0.001 |
| Geno_Status | 1 | 0.675 | 0.062 | 3.888 | < 0.001 |
| Location:Facility | 3 | 1.596 | 0.147 | 3.064 | < 0.001 |
| Residual | 37 | 6.423 | 0.590 | NA | NA |
| Total | 42 | 10.882 | 1 | NA | NA |
|  |  |  |  |  |  |
| **Unweighted UniFrac: Constrained** | | | | | |
|  | Df | SumOfSqs | R2 | F | Pr(>F) |
| Geno_Status | 1 | 1.874 | 0.172 | 8.529 | 0.434 |
| Residual | 41 | 9.008 | 0.828 | NA | NA |
| Total | 42 | 10.882 | 1 | NA | NA |

**Table S5:** Top 10 most significant ASVs contributing to fish gut microbiome dissimilarity between facility comparisons (SIMPER analysis, p-adj. < 0.05).

| Facility Comparison | Genus | Average Contribution (%) | p-adj. | Rank |
| --- | --- | --- | --- | --- |
| **Ore1 vs. Nor1** | | | | |
| Ore1 vs Nor1 | Cetobacterium | 25.09 | 0.0046 | 1 |
| Ore1 vs Nor1 | Pseudomonas | 9.5 | 0.0098 | 3 |
| Ore1 vs Nor1 | Lactococcus | 3.11 | 0.0175 | 5 |
| Ore1 vs Nor1 | Achromobacter | 2.45 | 0.0046 | 8 |
| Ore1 vs Nor1 | Pediococcus | 2.2 | 0.0046 | 9 |
| Ore1 vs Nor1 | Leuconostoc | 1.6 | 0.0046 | 13 |
| Ore1 vs Nor1 | Klebsiella | 0.33 | 0.0148 | 26 |
| Ore1 vs Nor1 | Limosilactobacillus | 0.26 | 0.0046 | 30 |
| Ore1 vs Nor1 | IMCC26207 | 0.21 | 0.0046 | 35 |
| Ore1 vs Nor1 | Nocardioides | 0.07 | 0.0255 | 59 |
| **Ore1 vs. Nor2A** | | | | |
| Ore1 vs Nor2A | Vibrio | 20.92 | 0.0325 | 1 |
| **Ore1 vs. Nor2B** | | | | |
| Ore1 vs Nor2B | Aeromonas | 27.54 | 0.0022 | 1 |
| Ore1 vs Nor2B | Cetobacterium | 25.7 | 0.005 | 2 |
| Ore1 vs Nor2B | Crenobacter | 0.63 | 0.0022 | 15 |
| Ore1 vs Nor2B | Chelativorans | 0.57 | 0.0022 | 17 |
| Ore1 vs Nor2B | Marmoricola | 0.34 | 0.0022 | 21 |
| Ore1 vs Nor2B | Chitinilyticum | 0.21 | 0.0022 | 29 |
| Ore1 vs Nor2B | Flavobacterium | 0.19 | 0.0022 | 30 |
| Ore1 vs Nor2B | Nannocystis | 0.18 | 0.0022 | 33 |
| Ore1 vs Nor2B | Pirellula | 0.15 | 0.0134 | 41 |
| Ore1 vs Nor2B | Fictibacillus | 0.12 | 0.0028 | 47 |
| **Ore2 vs. Nor2B** | | | | |
| Ore2 vs Nor2B | Enterococcus | 5.95 | 0.0139 | 4 |
| Ore2 vs Nor2B | Turicibacter | 0.07 | 0.0139 | 42 |
| Ore2 vs Nor2B | Gaiella | 0.02 | 0.0139 | 69 |
| Ore2 vs Nor2B | Erysipelatoclostridium | 0.01 | 0.0139 | 93 |
| Ore2 vs Nor2B | Desulfotomaculum | 0.01 | 0.0139 | 96 |
| Ore2 vs Nor2B | Eubacterium | 0.01 | 0.0139 | 97 |
| Ore2 vs Nor2B | Prevotella_9 | 0.01 | 0.0139 | 98 |
| **Nor2A vs. Nor1** | | | | |
| Nor2A vs Nor1 | Vibrio | 22.13 | 0.0108 | 1 |
| Nor2A vs Nor1 | Lactococcus | 3.48 | 0.0108 | 5 |
| Nor2A vs Nor1 | Pediococcus | 2.14 | 0.0163 | 11 |
| Nor2A vs Nor1 | Leuconostoc | 1.57 | 0.0108 | 13 |
| Nor2A vs Nor1 | Limosilactobacillus | 0.26 | 0.026 | 26 |
| Nor2A vs Nor1 | Levilactobacillus | 0.06 | 0.0487 | 43 |
| **Nor2B vs. Nor1** | | | | |
| Nor2B vs Nor1 | Crenobacter | 0.59 | 0.0163 | 18 |
| Nor2B vs Nor1 | Chelativorans | 0.56 | 0.0292 | 19 |
| Nor2B vs Nor1 | Marmoricola | 0.32 | 0.0292 | 26 |
| Nor2B vs Nor1 | Chitinilyticum | 0.2 | 0.0163 | 33 |
| Nor2B vs Nor1 | Flavobacterium | 0.19 | 0.0292 | 34 |
| Nor2B vs Nor1 | Nannocystis | 0.18 | 0.0292 | 35 |
| Nor2B vs Nor1 | Pirellula | 0.14 | 0.0352 | 37 |
| Nor2B vs Nor1 | Leptolyngbya ANT.L52.2 | 0.09 | 0.0292 | 48 |
| Nor2B vs Nor1 | OM60(NOR5) clade | 0.06 | 0.0292 | 62 |
| Nor2B vs Nor1 | Ellin6067 | 0.05 | 0.0292 | 68 |
| **Nor2A vs. Nor2B** | | | | |
| Nor2A vs Nor2B | Aeromonas | 20.06 | 0.0395 | 2 |
| Nor2A vs Nor2B | Crenobacter | 0.62 | 0.0163 | 13 |
| Nor2A vs Nor2B | Chelativorans | 0.55 | 0.0217 | 16 |
| Nor2A vs Nor2B | Marmoricola | 0.32 | 0.0217 | 18 |
| Nor2A vs Nor2B | Chitinilyticum | 0.2 | 0.0163 | 21 |
| Nor2A vs Nor2B | Flavobacterium | 0.2 | 0.0217 | 22 |
| Nor2A vs Nor2B | Nannocystis | 0.17 | 0.0275 | 23 |
| Nor2A vs Nor2B | Pirellula | 0.15 | 0.0217 | 24 |
| Nor2A vs Nor2B | Leptolyngbya ANT.L52.2 | 0.09 | 0.0266 | 34 |
| Nor2A vs Nor2B | Candidatus Alysiosphaera | 0.07 | 0.0275 | 38 |

**Figure S1: Bray-Curtis dissimilarity boxplots of paired fish and water samples separated by facility:** Comparison categories reflect the relationship between each fish tank-averaged community profile and tank water samples: same tank (water from the same tank as the fish), same facility (water from a different tank within the same facility), different facility (water from tanks not within the focus facility), and fish (tank-averaged fish gut microbiome comparisons within the same facility). Tukey-style box and whisker plots display the median (center horizontal line) and interquartile range, with whiskers extending ± 1.5 times the interquartile range; individual points represent pairwise distance values. No significance testing was performed due to inherent imbalance in the number of pairwise comparisons available across categories (same tank: Ore1 n=21, Nor1 n=7, Nor2A n=6, Nor2B n=3; same facility: Ore1 n=441, Nor1 n=56, Nor2A n=42, Nor2B n=9; different facility: Ore1 n=588, Nor1 n=387, Nor2A n=352, Nor2B n=188). Ore2 was excluded due to single-tank fish representation precluding meaningful within-facility comparisons.


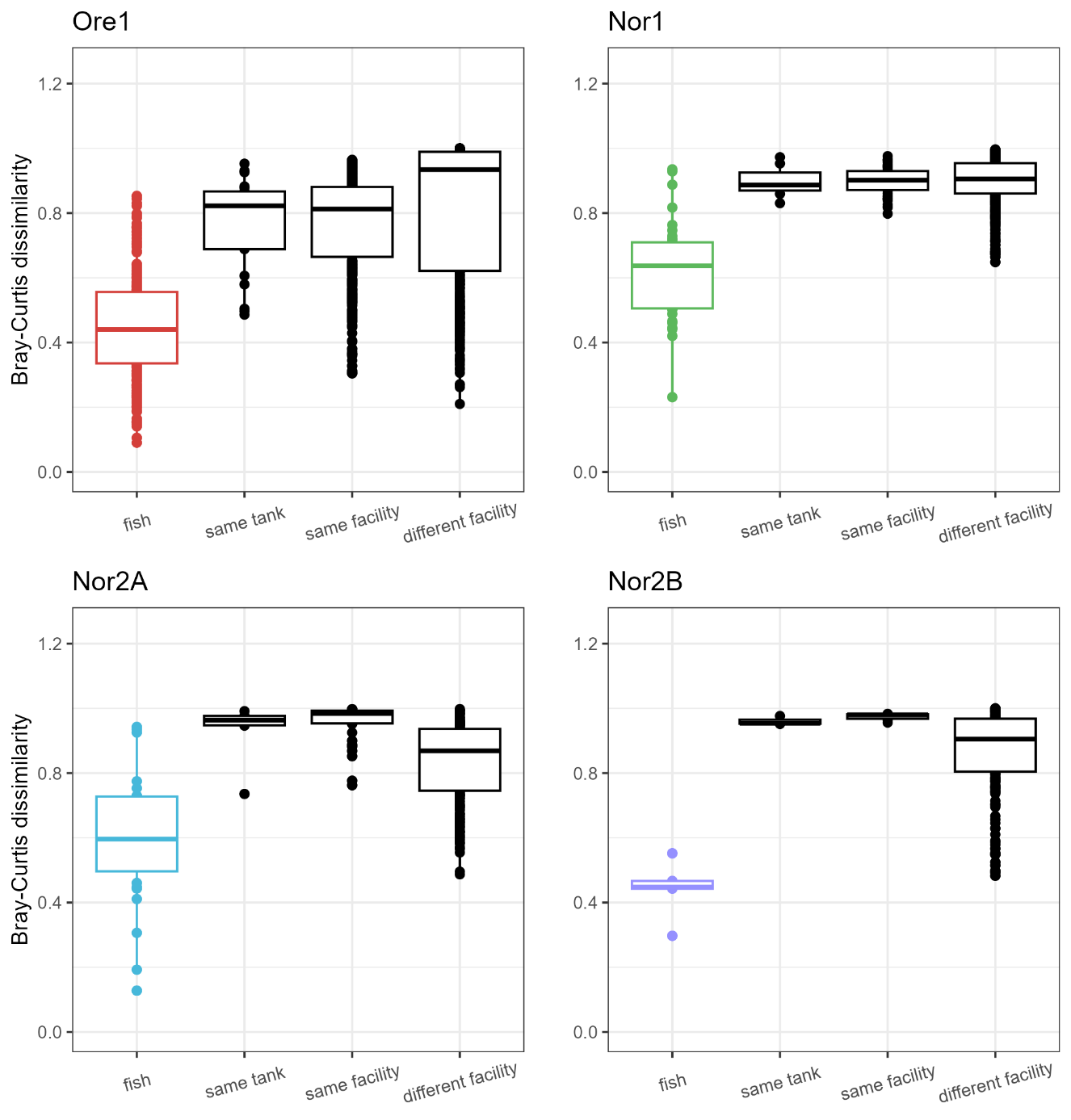


**Table S6:** Zebrafish lines and associated information

|  | Facility | | | | | | |  |  |
| --- | --- | --- | --- | --- | --- | --- | --- | --- | --- |
| **Genotype** | **Ore1** | | **Ore2** | **Nor1** | | **Nor2A** | **Nor2B** | **WT/GM Classifi-cation** | **Notes** |
| AB | X | | X |  | |  |  | WT | Standard laboratory strain, commonly used wild-type reference |
| ABC | X | |  |  | |  |  | WT | AB-related strain maintained without round robin breeding |
| WT |  | |  | X | |  | X | WT | Wild-type control strain; no additional strain information |
| NACRE |  | |  |  | | X |  | GM | MITF gene pigmentation mutant, transparent/translucent for imaging |
| HucGcamp6 |  | |  | X | | X |  | GM | Neuronal calcium indicator line (HuC promoter drives GCaMP6) |
| GMNC1 x GMNC |  | |  | X | |  |  | GM | Seizure/epilepsy model line (elevated photic response, network decay) |
| OMP x ChR2/3/4 |  | |  | X | |  |  | GM | Olfactory neuron optogenetics lines (olfactory marker protein promoter) |
| R2 |  | |  | X | |  |  | GM | Facility-specific line designation |
| Elipsa |  | |  | X | |  |  | GM | Loss-of-function mutation of traf3ip1 |
| npygRNA2 x vas x Gcamp6 |  | |  | X | |  |  | GM | Seizure/epilepsy model line (elevated photic response, network decay) |
| vas x Gcamp6 |  | |  | X | |  |  | GM | Germline calcium indicator (vasa promoter drives GCaMP6) |
| Foxjlb x Fauf x vas x GCatiP6s |  | |  | X | |  |  | GM | Multi-transgenic line for cilia/germline imaging |
| 6S |  |  | | | X |  |  | GM | Facility-specific line designation |
