# Supplementary material for: Aquaculture facility-specific microbiota shape the zebrafish gut microbiome
Source: Anim Microbiome. 2026 May 7;8:84. doi: 10.1186/s42523-026-00573-6 (PMC13321582; doi:10.1186/s42523-026-00573-6)
Supplement: Supplementary file 3 — Supplementary Material 3: Statistical analyses for tank water and zebrafish gut microbiomes across aquaculture facilities (R Markdown Document). html ouput file of complete R Markdown script [file 42523_2026_573_MOESM3_ESM.html]

Statistical analyses for tank water and zebrafish gut microbiomes across aquaculture facilities


# Statistical analyses for tank water and zebrafish gut microbiomes across aquaculture facilities

#### Evens, KC, Bakke, I, & Bohannan, BJM - Aquaculture facility-specific microbiota shape the zebrafish gut microbiome

#### 2025-10-13

This document includes all statistical analyses for the *Evens et
al.* manuscript. Due to the random iterative nature of some methods,
figure parameters may show minor variation between runs, but the main
findings are unaffected.

Load necessary libraries.

```
library("phyloseq")
library("ggplot2")
library("vegan")
library("dplyr")
library("tibble")
library("ggpubr")
library("gridExtra")
library("pairwiseAdonis")
library("microbiome")
library("FEAST")
library("kableExtra")
library("fantaxtic")
library("grid")
library("microViz")
library("tidyverse")
library("ggnested")
library("lme4")
library("lmerTest")
library("emmeans")
```

# Data upload

Load phyloseq object into working environment. The phyloseq object
(in RDS file format) is included as supplementary file information.

```
ps.22 <- readRDS("SupplementaryMaterials4.rds")

#remove negative control samples
ps.22 <- ps_filter(ps.22, Sample_Type != "CONTROL")

#Rename facility names
order = c("Huestis", "ZIRC", "Yaksi", "JutfeltA", "JutfeltB")
sample_data(ps.22)$Facility <- 
  factor(sample_data(ps.22)$FacilityB, levels = order, labels = c("Ore1", "Ore2", "Nor1", "Nor2A", "Nor2B"), ordered = TRUE)
```

# Prune taxa

```
ps.22 = prune_samples(sample_sums(ps.22) > 1000, ps.22)
```

# Defining sample groups and rarefying reads

Split phyloseq objects into two objects containing only either
‘water’ or ‘fish’ microbiome samples for separate analyses. An
additional phyloseq object containing no samples from the Ore2 facility
was created for sensitivity analyses.

```
# water
ps.22.w <- ps_filter(ps.22, Sample_Type == "water")
meta_water <- as.data.frame(sample_data(ps.22.w))

# fish
ps.22.f <- ps_filter(ps.22, Sample_Type == "fish")
meta_fish <- as.data.frame(sample_data(ps.22.f))

#fish, without facility Ore2
ps.22.noOre2 <- subset_samples(ps.22.f, Facility != "Ore2")
meta_noOre2 <- as.data.frame(sample_data(ps.22.noOre2))
```

Rarefy reads to even depth within sample groups.

```
# Rarefy water
ps_rare.w <- phyloseq::rarefy_even_depth(ps.22.w, rngseed = 123, replace = FALSE)
# Rarefy fish
ps_rare <- phyloseq::rarefy_even_depth(ps.22.f, rngseed = 123, replace = FALSE)         

# Count taxa
ntaxa(ps_rare.w)
```

```
## [1] 1607
```

```
ntaxa(ps_rare)
```

```
## [1] 1267
```

Define color palettes for figure creation .

```
locuszoom <- c("#D43f3aff", "#Eea236ff", "#5cb85cff", "#46b8daff", "#9590ff", "#357ebdff", "#b8b8b8ff")
locuszoom_grey <- c("#b8b8b8ff", "#D43f3aff", "#Eea236ff", "#5cb85cff", "#9590ff", "#357ebdff", "#46b8daff")

facility_order <- c("Ore1", "Ore2", "Nor1", "Nor2A", "Nor2B")
facility_colors <- setNames(locuszoom[1:5], facility_order)
```

#### Figure 2 - Water Microbiome Samples Alpha Diversity

Calculate alpha diversity values.

```
# Calculate diversity values
adiv.w <- data.frame(
  "Observed" = phyloseq::estimate_richness(ps.22.w, measures = "Observed"),
  "Shannon" = phyloseq::estimate_richness(ps.22.w, measures = "Shannon"),
  "InvSimpson" = phyloseq::estimate_richness(ps.22.w, measures = "InvSimpson"),
  "Facility" = phyloseq::sample_data(ps.22.w)$Facility)
```

Test for signficance with Kruskal-Wallis rank sum test followed by
pairwise comparisons using Wilcoxon rank sum exact test with BH p-value
correction.

```
kruskal.test(Shannon ~ Facility, data = adiv.w)
```

```
## 
##  Kruskal-Wallis rank sum test
## 
## data:  Shannon by Facility
## Kruskal-Wallis chi-squared = 5.581, df = 4, p-value = 0.2327
```

```
pairwise.wilcox.test(adiv.w$Shannon, adiv.w$Facility, p.adjust.method = "BH")
```

```
## 
##  Pairwise comparisons using Wilcoxon rank sum exact test 
## 
## data:  adiv.w$Shannon and adiv.w$Facility 
## 
##       Ore1  Ore2  Nor1  Nor2A
## Ore2  0.618 -     -     -    
## Nor1  0.366 0.618 -     -    
## Nor2A 1.000 0.823 0.082 -    
## Nor2B 1.000 1.000 0.083 1.000
## 
## P value adjustment method: BH
```

```
kruskal.test(InvSimpson ~ Facility, data = adiv.w)
```

```
## 
##  Kruskal-Wallis rank sum test
## 
## data:  InvSimpson by Facility
## Kruskal-Wallis chi-squared = 10.318, df = 4, p-value = 0.0354
```

```
pairwise.wilcox.test(adiv.w$InvSimpson, adiv.w$Facility, p.adjust.method = "BH")
```

```
## 
##  Pairwise comparisons using Wilcoxon rank sum exact test 
## 
## data:  adiv.w$InvSimpson and adiv.w$Facility 
## 
##       Ore1  Ore2  Nor1  Nor2A
## Ore2  0.958 -     -     -    
## Nor1  0.053 0.085 -     -    
## Nor2A 0.301 0.301 0.301 -    
## Nor2B 0.340 0.560 0.301 0.958
## 
## P value adjustment method: BH
```

Create alpha diversity plots.

```
## shannon diversity
adiv_plot_water_shan <- adiv.w %>%
  gather(key = metric, value = value, "Shannon") %>%
  mutate(metric = factor(metric, levels = "Shannon")) %>%
  ggplot(aes(x = Facility, y = value, fill = Facility, color = Facility)) +
  theme_bw() +
  theme(panel.grid.major = element_blank(), 
        panel.grid.minor = element_blank(),
        panel.background = element_rect(colour = "black", size= 0.5)) +
  geom_boxplot(width = 0.8, alpha = 0.6, outlier.shape = NA) +
  scale_fill_manual(values = locuszoom) +
  scale_color_manual(values = locuszoom) +
  geom_jitter(height = 0, width = 0.05, size = 1.2, alpha = 0.7) +
  labs(x = "", y = "Shannon index (H)") +
  theme(legend.position="none")+
  scale_x_discrete(labels = c('Ore1', 'Ore2', 'Nor1', 'Nor2A', 'Nor2B'))

## simpson diversity
adiv_plot_water_simp <- adiv.w %>%
  gather(key = metric, value = value, "InvSimpson") %>%
  mutate(metric = factor(metric, levels = "InvSimpson")) %>%
  ggplot(aes(x = Facility, y = value, fill = Facility, color = Facility)) +
  theme_bw() +
  theme(panel.grid.major = element_blank(), 
        panel.grid.minor = element_blank(),
        panel.background = element_rect(colour = "black", size= 0.5)) +
  geom_boxplot(width = 0.8, alpha = 0.6, outlier.shape = NA) +
  scale_fill_manual(values = locuszoom) +
  scale_color_manual(values = locuszoom) +
  geom_jitter(height = 0, width = 0.05, size = 1.2, alpha = 0.7) +
  labs(x = "", y = "Inverse Simpson index (1-λ)") +
  theme(legend.position="none") +
  scale_x_discrete(labels = c('Ore1', 'Ore2', 'Nor1', 'Nor2A', 'Nor2B'))
```

Create combined figure of both Shannon and Inverse Simpson alpha
diversity plots.

```
plots_water_adiv <- ggarrange(adiv_plot_water_shan, adiv_plot_water_simp, ncol = 2, nrow = 1, labels = "AUTO")
plots_water_adiv
```

```
#ggsave("Figures/25_AM/Figure2.png", plots_water_adiv, height = 105, width = 170, units = ("mm"))
```

#### Figure 5 - Fish Gut Microbiome Samples Alpha Diversity

Repeat process to calculate alpha diversity as above for fish gut
microbiome samples. This includes an accompanying exclusion-based
sensitivity analysis to compare alpha diversity results with and without
the Ore2 facility.

```
# Estimate richness
alpha_fish <- estimate_richness(ps.22.f, measures = c("Shannon", "Observed", "InvSimpson"))

# Move data into new dataframe
alpha_fish$Tank <- meta_fish$TankID
alpha_fish$Facility <- meta_fish$Facility
alpha_fish$Facility <- as.character(alpha_fish$Facility)
```

```
#Mixed model — tank as random effect
fish_shannon <- lmer(Shannon ~ Facility + (1 | Tank), data = alpha_fish)

summary(fish_shannon)
```

```
## Linear mixed model fit by REML. t-tests use Satterthwaite's method [
## lmerModLmerTest]
## Formula: Shannon ~ Facility + (1 | Tank)
##    Data: alpha_fish
## 
## REML criterion at convergence: 323.8
## 
## Scaled residuals: 
##     Min      1Q  Median      3Q     Max 
## -1.9943 -0.6780 -0.1380  0.5158  2.4773 
## 
## Random effects:
##  Groups   Name        Variance Std.Dev.
##  Tank     (Intercept) 0.1362   0.3691  
##  Residual             0.5446   0.7379  
## Number of obs: 134, groups:  Tank, 43
## 
## Fixed effects:
##               Estimate Std. Error      df t value Pr(>|t|)    
## (Intercept)     1.7085     0.1755 28.6578   9.736 1.36e-10 ***
## FacilityNor2A  -0.8644     0.2682 34.7607  -3.223  0.00276 ** 
## FacilityNor2B   0.2551     0.3184 29.9134   0.801  0.42932    
## FacilityOre1    0.1542     0.2179 33.1346   0.708  0.48406    
## FacilityOre2    0.1561     0.5253 23.4296   0.297  0.76892    
## ---
## Signif. codes:  0 '***' 0.001 '**' 0.01 '*' 0.05 '.' 0.1 ' ' 1
## 
## Correlation of Fixed Effects:
##             (Intr) FclN2A FclN2B FcltO1
## FaciltyNr2A -0.654                     
## FaciltyNr2B -0.551  0.361              
## FacilityOr1 -0.805  0.527  0.444       
## FacilityOr2 -0.334  0.219  0.184  0.269
```

```
anova(fish_shannon) # Type III F-tests via lmerTest
```

```
## Type III Analysis of Variance Table with Satterthwaite's method
##          Sum Sq Mean Sq NumDF  DenDF F value   Pr(>F)   
## Facility 10.992  2.7481     4 30.643  5.0465 0.003041 **
## ---
## Signif. codes:  0 '***' 0.001 '**' 0.01 '*' 0.05 '.' 0.1 ' ' 1
```

```
emmeans(fish_shannon, pairwise ~ Facility, adjust = "BH")
```

```
## $emmeans
##  Facility emmean    SE   df lower.CL upper.CL
##  Nor1      1.709 0.176 27.8    1.348     2.07
##  Nor2A     0.844 0.203 39.4    0.434     1.25
##  Nor2B     1.964 0.266 29.6    1.420     2.51
##  Ore1      1.863 0.129 43.0    1.602     2.12
##  Ore2      1.865 0.495 22.2    0.838     2.89
## 
## Degrees-of-freedom method: kenward-roger 
## Confidence level used: 0.95 
## 
## $contrasts
##  contrast      estimate    SE   df t.ratio p.value
##  Nor1 - Nor2A   0.86436 0.269 33.8   3.218  0.0095
##  Nor1 - Nor2B  -0.25515 0.319 29.0  -0.801  0.8081
##  Nor1 - Ore1   -0.15422 0.218 32.2  -0.707  0.8081
##  Nor1 - Ore2   -0.15612 0.525 22.7  -0.297  0.9574
##  Nor2A - Nor2B -1.11950 0.335 32.8  -3.347  0.0095
##  Nor2A - Ore1  -1.01857 0.241 40.4  -4.231  0.0013
##  Nor2A - Ore2  -1.02048 0.535 23.9  -1.907  0.1715
##  Nor2B - Ore1   0.10093 0.296 31.7   0.341  0.9574
##  Nor2B - Ore2   0.09902 0.562 23.6   0.176  0.9574
##  Ore1 - Ore2   -0.00191 0.512 23.0  -0.004  0.9971
## 
## Degrees-of-freedom method: kenward-roger 
## P value adjustment: BH method for 10 tests
```

```
# Mixed model — tank as random effect
fish_invsimp <- lmer(InvSimpson ~ Facility + (1 | Tank), data = alpha_fish)

summary(fish_invsimp)
```

```
## Linear mixed model fit by REML. t-tests use Satterthwaite's method [
## lmerModLmerTest]
## Formula: InvSimpson ~ Facility + (1 | Tank)
##    Data: alpha_fish
## 
## REML criterion at convergence: 756.8
## 
## Scaled residuals: 
##     Min      1Q  Median      3Q     Max 
## -1.0692 -0.5486 -0.2159  0.1492  6.7754 
## 
## Random effects:
##  Groups   Name        Variance Std.Dev.
##  Tank     (Intercept)  1.76    1.327   
##  Residual             16.98    4.121   
## Number of obs: 134, groups:  Tank, 43
## 
## Fixed effects:
##               Estimate Std. Error      df t value Pr(>|t|)    
## (Intercept)     4.8890     0.8232 19.1269   5.939 9.95e-06 ***
## FacilityNor2A  -2.9112     1.2820 25.8012  -2.271   0.0317 *  
## FacilityNor2B  -1.4604     1.5015 20.9496  -0.973   0.3418    
## FacilityOre1   -0.3831     1.0367 23.8903  -0.370   0.7150    
## FacilityOre2   -1.2398     2.4155 14.6500  -0.513   0.6154    
## ---
## Signif. codes:  0 '***' 0.001 '**' 0.01 '*' 0.05 '.' 0.1 ' ' 1
## 
## Correlation of Fixed Effects:
##             (Intr) FclN2A FclN2B FcltO1
## FaciltyNr2A -0.642                     
## FaciltyNr2B -0.548  0.352              
## FacilityOr1 -0.794  0.510  0.435       
## FacilityOr2 -0.341  0.219  0.187  0.271
```

```
anova(fish_invsimp) # Type III F-tests via lmerTest
```

```
## Type III Analysis of Variance Table with Satterthwaite's method
##          Sum Sq Mean Sq NumDF  DenDF F value Pr(>F)
## Facility 106.56  26.641     4 21.253  1.5687  0.219
```

```
emmeans(fish_invsimp, pairwise ~ Facility, adjust = "BH")
```

```
## $emmeans
##  Facility emmean    SE   df lower.CL upper.CL
##  Nor1       4.89 0.827 24.5   3.1846     6.59
##  Nor2A      1.98 0.984 40.4  -0.0112     3.97
##  Nor2B      3.43 1.260 27.8   0.8539     6.00
##  Ore1       4.51 0.631 45.0   3.2346     5.78
##  Ore2       3.65 2.270 18.4  -1.1139     8.41
## 
## Degrees-of-freedom method: kenward-roger 
## Confidence level used: 0.95 
## 
## $contrasts
##  contrast      estimate   SE   df t.ratio p.value
##  Nor1 - Nor2A     2.911 1.29 32.5   2.265  0.1821
##  Nor1 - Nor2B     1.460 1.50 26.8   0.971  0.8002
##  Nor1 - Ore1      0.383 1.04 30.3   0.368  0.8002
##  Nor1 - Ore2      1.240 2.42 19.0   0.513  0.8002
##  Nor2A - Nor2B   -1.451 1.60 31.9  -0.909  0.8002
##  Nor2A - Ore1    -2.528 1.17 41.6  -2.162  0.1821
##  Nor2A - Ore2    -1.671 2.48 20.6  -0.675  0.8002
##  Nor2B - Ore1    -1.077 1.41 30.4  -0.766  0.8002
##  Nor2B - Ore2    -0.221 2.60 20.2  -0.085  0.9331
##  Ore1 - Ore2      0.857 2.36 19.5   0.363  0.8002
## 
## Degrees-of-freedom method: kenward-roger 
## P value adjustment: BH method for 10 tests
```

```
# reorder facility names
alpha_fish$Facility <- factor(alpha_fish$Facility, levels = facility_order)

# Shannon
cld_shannon <- data.frame(
  Facility = factor(facility_order, levels = facility_order),
  label = c("a", "a", "a", "b", "a")  
)

# InvSimpson
cld_invsimpson <- data.frame(
  Facility = factor(facility_order, levels = facility_order),
  label = c("a", "a", "a", "a", "a")  # adjust once you have InvSimpson results
)
```

```
theme_adiv <- theme_bw() +
  theme(
    panel.grid.major = element_blank(),
    panel.grid.minor = element_blank(),
    panel.background = element_rect(colour = "black", linewidth = 0.5),
    legend.position = "none",
    axis.title = element_text(size = 11),
    axis.text = element_text(size = 10),
    axis.text.x = element_text(angle = 45, hjust = 1)
  )

# letter y-positions
letter_y_shannon <- max(alpha_fish$Shannon, na.rm = TRUE) * 1.08
letter_y_invsimpson <- max(alpha_fish$InvSimpson, na.rm = TRUE) * 1.08
```

Create panels for Shannon and InvSimpson results.

```
# Shannon panel
pfish_shannon <- ggplot(alpha_fish, aes(x = Facility, y = Shannon,
                                     fill = Facility, color = Facility)) +
  theme_adiv +
  geom_boxplot(width = 0.8, alpha = 0.6, outlier.shape = NA) +
  geom_jitter(height = 0, width = 0.05, size = 1.2, alpha = 0.7) +
  scale_fill_manual(values = facility_colors) +
  scale_color_manual(values = facility_colors) +
  geom_text(data = cld_shannon,
            aes(x = Facility, y = letter_y_shannon, label = label),
            inherit.aes = FALSE, size = 4, fontface = "bold") +
  labs(x = "", y = "Shannon index (H)")

# Inverse Simpson panel
pfish_invsimpson <- ggplot(alpha_fish, aes(x = Facility, y = InvSimpson,
                                        fill = Facility, color = Facility)) +
  theme_adiv +
  geom_boxplot(width = 0.8, alpha = 0.6, outlier.shape = NA) +
  geom_jitter(height = 0, width = 0.05, size = 1.2, alpha = 0.7) +
  scale_fill_manual(values = facility_colors) +
  scale_color_manual(values = facility_colors) +
  #geom_text(data = cld_invsimpson,
  #          aes(x = Facility, y = letter_y_invsimpson, label = label),
  #          inherit.aes = FALSE, size = 4, fontface = "bold") +
  labs(x = "", y = "Inverse Simpson index (1-λ)")
```

Combine panels into single plot and print.

```
plot_fish_adiv <- ggarrange(pfish_shannon, pfish_invsimpson, ncol = 2, nrow = 1, labels = "AUTO")
plot_fish_adiv
```

```
#ggsave("Figures/25_AM/Figure5.png", plot_fish_adiv, height = 105, width = 170, units = ("mm"))
```

##### Supplementary Table S1 - Estimated Marginal Means

```
# Shannon
emm_output <- emmeans(fish_shannon, pairwise ~ Facility, adjust = "BH")

emm_df <- as.data.frame(emm_output$emmeans)
con_df <- as.data.frame(emm_output$contrasts)

# Add a label column to identify each section
emm_df$section <- "Estimated Marginal Means"
con_df$section <- "Pairwise Contrasts"

# Combine with dplyr
combined <- dplyr::bind_rows(emm_df, con_df)

write.csv(combined, "Figures/25_AM/S1F_shannon_emmeans_full.csv", row.names = FALSE)

# InvSimpson
emm_output_invsimp <- emmeans(fish_invsimp, pairwise ~ Facility, adjust = "BH")

emm_df_invsimp <- as.data.frame(emm_output_invsimp$emmeans)
con_df_invsimp <- as.data.frame(emm_output_invsimp$contrasts)

emm_df_invsimp$section <- "Estimated Marginal Means"
con_df_invsimp$section <- "Pairwise Contrasts"

combined_invsimp <- dplyr::bind_rows(emm_df_invsimp, con_df_invsimp)

write.csv(combined_invsimp, "Figures/25_AM/S1F_invsimp_emmeans_full.csv", row.names = FALSE)
```

##### Ore2 Exclusion-based Sensitivity Test

```
# Estimate richness
alpha_fish_sens <- estimate_richness(ps.22.noOre2, measures = c("Shannon", "Observed", "InvSimpson"))

# Move data into new dataframe
alpha_fish_sens$Tank <- meta_noOre2$TankID
alpha_fish_sens$Facility <- meta_noOre2$Facility
alpha_fish_sens$Facility <- as.character(alpha_fish_sens$Facility)


# Mixed model Shannon — tank as random effect
fish_shannon_sens <- lmer(Shannon ~ Facility + (1 | Tank), data = alpha_fish_sens)

summary(fish_shannon_sens)

anova_shannon_sens <- as.data.frame(anova(fish_shannon_sens)) # Type III F-tests via lmerTest
write.csv(anova_shannon_sens, "Figures/25_AM/S2_anova_shannon.csv", row.names = FALSE)

# Save results as data frame
emm_output_shannon_sens <- emmeans(fish_shannon_sens, pairwise ~ Facility, adjust = "BH")
emm_shannon_sens <- as.data.frame(emm_output_shannon_sens$emmeans)
con_shannon_sens <- as.data.frame(emm_output_shannon_sens$contrasts)

# Combine and print
combined_shannon_sens <- dplyr::bind_rows(emm_shannon_sens, con_shannon_sens)
write.csv(combined_shannon_sens, "Figures/25_AM/S2_emmeans_shannon.csv", row.names = FALSE)

# Mixed model InvSimpson — tank as random effect
fish_invsimp_sens <- lmer(InvSimpson ~ Facility + (1 | Tank), data = alpha_fish_sens)

summary(fish_invsimp_sens)

# Save anova output
anova(fish_invsimp_sens) # Type III F-tests via lmerTest
anova_invsimp_sens <- as.data.frame(anova(fish_invsimp_sens)) # Type III F-tests via lmerTest
write.csv(anova_invsimp_sens, "Figures/25_AM/S2_anova_invsimp.csv", row.names = FALSE)

# Save emmeans output
emm_output_invsimp_sens <- emmeans(fish_invsimp_sens, pairwise ~ Facility, adjust = "BH") 
emm_invsimp_sens <- as.data.frame(emm_output_invsimp_sens$emmeans)
con_invsimp_sens <- as.data.frame(emm_output_invsimp_sens$contrasts)

# Combine and print
combined_invsimp_sens <- dplyr::bind_rows(emm_invsimp_sens, con_invsimp_sens)
write.csv(combined_invsimp_sens, "Figures/25_AM/S2_emmeans_invsimp.csv", row.names = FALSE)
```

# Beta Diversity

## Water Microbiome Samples

### PERMANOVA

Calculate Bray-Curtis and unweighted UniFrac distance values. Create
new metadata table specific for rarefied water microbiome samples.

```
dist.w.uni <- phyloseq::distance(ps_rare.w, method = "unifrac")
dist.w.bray <- phyloseq::distance(ps_rare.w, method = "bray")

# set up metadata table for rarefied data
meta_rare.w <- data.frame(sample_data(ps_rare.w))
```

##### Beta-dispersion test

```
bd_water_bc <- betadisper(dist.w.bray, meta_rare.w$Facility)
permutest(bd_water_bc, 
          permutations = how(nperm = 9999))
```

```
## 
## Permutation test for homogeneity of multivariate dispersions
## Permutation: free
## Number of permutations: 9999
## 
## Response: Distances
##           Df  Sum Sq  Mean Sq      F N.Perm Pr(>F)  
## Groups     4 0.17806 0.044514 4.1091   9999 0.0113 *
## Residuals 45 0.48748 0.010833                       
## ---
## Signif. codes:  0 '***' 0.001 '**' 0.01 '*' 0.05 '.' 0.1 ' ' 1
```

```
bd_water_uni <- betadisper(dist.w.uni, meta_rare.w$Facility)
permutest(bd_water_uni, 
          permutations = how(nperm = 9999))
```

```
## 
## Permutation test for homogeneity of multivariate dispersions
## Permutation: free
## Number of permutations: 9999
## 
## Response: Distances
##           Df   Sum Sq   Mean Sq      F N.Perm Pr(>F)  
## Groups     4 0.061726 0.0154315 3.9088   9999 0.0141 *
## Residuals 45 0.177654 0.0039479                       
## ---
## Signif. codes:  0 '***' 0.001 '**' 0.01 '*' 0.05 '.' 0.1 ' ' 1
```

```
# Pairwise comparisons of dispersion
permutest(bd_water_bc, pairwise = TRUE, permutations = how(nperm = 9999))
```

```
## 
## Permutation test for homogeneity of multivariate dispersions
## Permutation: free
## Number of permutations: 9999
## 
## Response: Distances
##           Df  Sum Sq  Mean Sq      F N.Perm Pr(>F)  
## Groups     4 0.17806 0.044514 4.1091   9999 0.0105 *
## Residuals 45 0.48748 0.010833                       
## ---
## Signif. codes:  0 '***' 0.001 '**' 0.01 '*' 0.05 '.' 0.1 ' ' 1
## 
## Pairwise comparisons:
## (Observed p-value below diagonal, permuted p-value above diagonal)
##            Ore1      Ore2      Nor1     Nor2A  Nor2B
## Ore1            0.0017000 0.0206000 0.3051000 0.0041
## Ore2  0.0016862           0.3178000 0.1950000 0.9306
## Nor1  0.0204395 0.3176979           0.4689000 0.0773
## Nor2A 0.3088975 0.2009906 0.4755146           0.1883
## Nor2B 0.0037406 0.9343127 0.0788712 0.1811981
```

```
permutest(bd_water_uni, pairwise = TRUE, permutations = how(nperm = 9999))
```

```
## 
## Permutation test for homogeneity of multivariate dispersions
## Permutation: free
## Number of permutations: 9999
## 
## Response: Distances
##           Df   Sum Sq   Mean Sq      F N.Perm Pr(>F)  
## Groups     4 0.061726 0.0154315 3.9088   9999 0.0135 *
## Residuals 45 0.177654 0.0039479                       
## ---
## Signif. codes:  0 '***' 0.001 '**' 0.01 '*' 0.05 '.' 0.1 ' ' 1
## 
## Pairwise comparisons:
## (Observed p-value below diagonal, permuted p-value above diagonal)
##             Ore1       Ore2       Nor1      Nor2A  Nor2B
## Ore1             8.0290e-01 3.0260e-01 1.0000e-04 0.0511
## Ore2  7.9054e-01            4.6530e-01 1.0600e-02 0.2322
## Nor1  3.0745e-01 4.5673e-01            3.6100e-02 0.4899
## Nor2A 3.0174e-05 1.1760e-02 4.2240e-02            0.2323
## Nor2B 4.5696e-02 2.3777e-01 4.7763e-01 2.3054e-01
```

```
# For visualization
boxplot(bd_water_bc, main = "Bray-Curtis dispersion by facility")
```

```
boxplot(bd_water_uni, main = "UniFrac dispersion by facility")
```

##### Supplementary Table S2 - Bray-Curtis

Construct the hierarchical and constrained PERMANOVA. A hierarchical
PERMANOVA was first performed using the adonis function with Location as
the primary factor and Facility nested within Location (perm.w1),
followed by Genotype, using Bray-Curtis distance values. We then
performed a constrained PERMANOVA with Genotype as the constraint term
and Facility as a conditioning variable to isolate the effect of
Genotype while accounting for facility-level variation (perm.w2). All
tests used 9999 permutations.

```
perm.w1 <- adonis2(dist.w.bray ~ Location/Facility + Geno_Status, data = meta_rare.w, permutations = 9999)

# # Convert the result to a data frame
# result_perm.w1 <- as.data.frame(perm.w1)
# 
# # Save as CSV
# write.csv(result_perm.w1, "Figures/25_AM/S2WaterBray_hier.csv", row.names = TRUE)

##### 
perm.w2 <- adonis2(dist.w.bray ~ Geno_Status, data= meta_rare.w, strata = meta_rare.w$Facility, permutations = 9999)

# # Convert the result to a data frame
# result_perm.w2 <- as.data.frame(perm.w2)
# 
# # Save as CSV
# write.csv(result_perm.w2, "Figures/25_AM/S2WaterBray_cons.csv", row.names = TRUE)
```

##### Supplementary Table S2 - Unweighted UniFrac

Repeat using unweighted UniFrac distance values.

```
perm.w3 <- adonis2(dist.w.uni ~ Location/Facility + Geno_Status, data = meta_rare.w, permutations = 9999)

#  Convert the result to a data frame
# result_perm.w3 <- as.data.frame(perm.w3)

#  Save as CSV
# write.csv(result_perm.w3, "Figures/25_AM/S2WaterUni_hier.csv", row.names = TRUE)

perm.w4 <- adonis2(dist.w.uni ~ Geno_Status, data= meta_rare.w, strata = meta_rare.w$Facility, permutations = 9999)

#  Convert the result to a data frame
# result_perm.w4 <- as.data.frame(perm.w4)

#  Save as CSV
# write.csv(result_perm.w4, "Figures/25_AM/S2WaterUni_cons.csv", row.names = TRUE)
```

Create the figure by stacking each PERMANOVA output.

```
stacked_permanova <- bind_rows(
  # Bray-Curtis tables
  perm.w1 %>% mutate(Analysis = "BC_Hierarchical"),
  perm.w2 %>% mutate(Analysis = "BC_Constrained"),
  # UniFrac tables
  perm.w3 %>% mutate(Analysis = "UF_Hierarchical"),
  perm.w4 %>% mutate(Analysis = "UF_Constrained")
) %>%
  rename_with(~ gsub("\\.\\.\\.\\d+$", "", .x)) %>%
  select(-Analysis) %>%
  kbl(caption = "Tank Water PERMANOVA Results") %>%
  kable_classic(full_width = F, html_font = "Cambria") %>%
  # Bray-Curtis section
  pack_rows("Bray-Curtis Dissimilarity", 
            1, nrow(perm.w1) + nrow(perm.w2), 
            label_row_css = "background-color: #d0d0d0; font-weight: bold;") %>%
  pack_rows("A) Hierarchical PERMANOVA: Location, Facility, and Genotype Effects", 
            1, nrow(perm.w1), 
            label_row_css = "background-color: #f0f0f0;") %>%
  pack_rows("B) Constrained PERMANOVA: Genotype Effect within Facilities", 
            nrow(perm.w1) + 1, nrow(perm.w1) + nrow(perm.w2), 
            label_row_css = "background-color: #f0f0f0;") %>%
  # UniFrac section
  pack_rows("Unweighted UniFrac Distance", 
            nrow(perm.w1) + nrow(perm.w2) + 1, 
            nrow(perm.w1) + nrow(perm.w2) + nrow(perm.w3) + nrow(perm.w4), 
            label_row_css = "background-color: #d0d0d0; font-weight: bold;") %>%
  pack_rows("A) Hierarchical PERMANOVA: Location, Facility, and Genotype Effects", 
            nrow(perm.w1) + nrow(perm.w2) + 1, 
            nrow(perm.w1) + nrow(perm.w2) + nrow(perm.w3), 
            label_row_css = "background-color: #f0f0f0;") %>%
  pack_rows("B) Constrained PERMANOVA: Genotype Effect within Facilities", 
            nrow(perm.w1) + nrow(perm.w2) + nrow(perm.w3) + 1, 
            nrow(perm.w1) + nrow(perm.w2) + nrow(perm.w3) + nrow(perm.w4), 
            label_row_css = "background-color: #f0f0f0;")

# Display the table
stacked_permanova
```

Tank Water PERMANOVA Results

|  | Df | SumOfSqs | R2 | F | Pr(>F) |
| --- | --- | --- | --- | --- | --- |
| **Bray-Curtis Dissimilarity** | | | | | |
| **A) Hierarchical PERMANOVA: Location, Facility, and Genotype Effects** | | | | | |
| Location…1 | 1 | 3.5375180 | 0.2042614 | 17.141287 | 0.0001 |
| Geno\_Status…2 | 1 | 0.7346309 | 0.0424186 | 3.559705 | 0.0006 |
| Location:Facility…3 | 3 | 3.9659760 | 0.2290011 | 6.405803 | 0.0001 |
| Residual…4 | 44 | 9.0804611 | 0.5243189 | NA | NA |
| Total…5 | 49 | 17.3185859 | 1.0000000 | NA | NA |
| **B) Constrained PERMANOVA: Genotype Effect within Facilities** | | | | | |
| Geno\_Status…6 | 1 | 2.7238117 | 0.1572768 | 8.958204 | 0.4314 |
| Residual…7 | 48 | 14.5947742 | 0.8427232 | NA | NA |
| Total…8 | 49 | 17.3185859 | 1.0000000 | NA | NA |
| **Unweighted UniFrac Distance** | | | | | |
| **A) Hierarchical PERMANOVA: Location, Facility, and Genotype Effects** | | | | | |
| Location…9 | 1 | 1.6592834 | 0.1325764 | 9.026910 | 0.0001 |
| Geno\_Status…10 | 1 | 0.5007333 | 0.0400085 | 2.724113 | 0.0008 |
| Location:Facility…11 | 3 | 2.2677896 | 0.1811959 | 4.112444 | 0.0001 |
| Residual…12 | 44 | 8.0878692 | 0.6462191 | NA | NA |
| Total…13 | 49 | 12.5156756 | 1.0000000 | NA | NA |
| **B) Constrained PERMANOVA: Genotype Effect within Facilities** | | | | | |
| Geno\_Status…14 | 1 | 1.4707108 | 0.1175095 | 6.391520 | 0.2851 |
| Residual…15 | 48 | 11.0449648 | 0.8824905 | NA | NA |
| Total…16 | 49 | 12.5156756 | 1.0000000 | NA | NA |

```
# Save as PNG 
# stacked_permanova %>%
#   save_kable("Figures/25_AM/Supp1_PERM_Water.png", 
#              zoom = 3,
#              vwidth = 2000)
```

### Table 1 - Pairwise PERMANOVA

Construct pairwise PERMANOVA models for facilities using Bray-Curtis
and unweighted UniFrac distance values.

```
# Bray-Curtis
set.seed(100)
pairperm.w1 <- pairwise.adonis(dist.w.bray, factors = meta_rare.w$Facility, sim.function = "vegdist", sim.method = "bray", p.adjust.m = "BH", reduce = NULL, perm = 9999)

#  Convert the result to a data frame
# result_pairperm.w1 <- as.data.frame(pairperm.w1)

#  Save as CSV
# write.csv(result_pairperm.w1, "Figures/25_AM/Table1Bray.csv", row.names = TRUE)

# Unweighted UniFrac
set.seed(100)
pairperm.w2 <- pairwise.adonis(dist.w.uni, factors = meta_rare.w$Facility, sim.function = "vegdist", sim.method = "unifrac", p.adjust.m = "BH", reduce = NULL, perm = 9999)

#  Convert the result to a data frame
# result_pairperm.w2 <- as.data.frame(pairperm.w2)

#  Save as CSV
# write.csv(result_pairperm.w2, "Figures/25_AM/Table1UF.csv", row.names = TRUE)
```

Create tables of pairwise PERMANOVA outputs.

```
pairperm.w1 %>%
  kbl(caption = "Bray-Curtis") %>%
  kable_classic(full_width = F, html_font = "Cambria")
```

Bray-Curtis

| pairs | Df | SumsOfSqs | F.Model | R2 | p.value | p.adjusted | sig |
| --- | --- | --- | --- | --- | --- | --- | --- |
| Ore1 vs Ore2 | 1 | 1.8969672 | 9.169330 | 0.2227223 | 0.0001 | 0.0002000 | \*\* |
| Ore1 vs Nor2A | 1 | 2.5022961 | 10.476217 | 0.2872068 | 0.0001 | 0.0002000 | \*\* |
| Ore1 vs Nor2B | 1 | 1.6181380 | 6.949447 | 0.2320392 | 0.0003 | 0.0005000 | \*\* |
| Ore1 vs Nor1 | 1 | 2.4401921 | 10.692511 | 0.2836773 | 0.0001 | 0.0002000 | \*\* |
| Ore2 vs Nor2A | 2 | 2.5206440 | 6.819226 | 0.4762287 | 0.0001 | 0.0002000 | \*\* |
| Ore2 vs Nor2B | 1 | 1.6991348 | 11.519734 | 0.4698148 | 0.0029 | 0.0036250 |  |
| Ore2 vs Nor1 | 1 | 2.3310749 | 14.548562 | 0.4611482 | 0.0001 | 0.0002000 | \*\* |
| Nor2A vs Nor2B | 1 | 0.9012598 | 4.185003 | 0.3741620 | 0.0104 | 0.0104000 | . |
| Nor2A vs Nor1 | 1 | 1.5365291 | 7.304557 | 0.3990567 | 0.0005 | 0.0007143 | \*\* |
| Nor2B vs Nor1 | 1 | 1.3892597 | 7.617283 | 0.4877470 | 0.0076 | 0.0084444 |  |


```
pairperm.w2 %>%
  kbl(caption = "Unweighted UniFrac") %>%
  kable_classic(full_width = F, html_font = "Cambria")
```

Unweighted UniFrac

| pairs | Df | SumsOfSqs | F.Model | R2 | p.value | p.adjusted | sig |
| --- | --- | --- | --- | --- | --- | --- | --- |
| Ore1 vs Ore2 | 1 | 1.0875238 | 5.662939 | 0.1503584 | 0.0001 | 0.0002000 | \*\* |
| Ore1 vs Nor2A | 1 | 1.6730056 | 9.641490 | 0.2705131 | 0.0001 | 0.0002000 | \*\* |
| Ore1 vs Nor2B | 1 | 0.8548434 | 4.584490 | 0.1661981 | 0.0003 | 0.0005000 | \*\* |
| Ore1 vs Nor1 | 1 | 1.0550601 | 5.675710 | 0.1736981 | 0.0001 | 0.0002000 | \*\* |
| Ore2 vs Nor2A | 2 | 1.4109888 | 3.709261 | 0.3309104 | 0.0001 | 0.0002000 | \*\* |
| Ore2 vs Nor2B | 1 | 0.7306389 | 3.611399 | 0.2174049 | 0.0029 | 0.0036250 |  |
| Ore2 vs Nor1 | 1 | 0.8705382 | 4.403913 | 0.2057527 | 0.0001 | 0.0002000 | \*\* |
| Nor2A vs Nor2B | 1 | 0.7115400 | 4.999297 | 0.4166325 | 0.0104 | 0.0104000 | . |
| Nor2A vs Nor1 | 1 | 0.8670276 | 5.523558 | 0.3342838 | 0.0005 | 0.0007143 | \*\* |
| Nor2B vs Nor1 | 1 | 0.6487153 | 3.451121 | 0.3013785 | 0.0076 | 0.0084444 |  |

## Fish Gut Microbiome Samples

### PERMANOVA

Calculate Bray-Curtis and unweighted UniFrac distance values. Create
new metadata table specific for rarefied fish gut microbiome
samples.

```
meta_rare <- data.frame(sample_data(ps_rare))


tank_means <- aggregate(. ~ TankID, 
                        data = cbind(TankID = meta_rare$TankID, 
                        as.data.frame(otu_table(ps_rare))), 
                        FUN = mean)

rownames(tank_means) <- tank_means$TankID
tank_means$TankID <- NULL

# create metadata table to match averages
meta_tank <- meta_rare[!duplicated(meta_rare$TankID), ]
meta_tank <- meta_tank[match(rownames(tank_means), meta_tank$TankID), ]
```

```
otu_tank <- otu_table(as.matrix(tank_means), taxa_are_rows = FALSE)
sample_tank <- sample_data(meta_tank)
rownames(sample_tank) <- meta_tank$TankID
ps_tank <- phyloseq(otu_tank, sample_tank, phy_tree(ps_rare), tax_table(ps_rare))
```

```
dist.f.bray <- phyloseq::distance(ps_tank, method = "bray")
dist.f.uni <- phyloseq::distance(ps_tank, method = "unifrac")
```

##### Supplementary Table 4 - Bray-Curtis

Create hierarchical and constrained PERMANOVA models using
Bray-Curtis distance values to test the effects of Location, Facility,
and Genotype as done earlier with water microbiome samples.

```
perm.f1 <- adonis2(dist.f.bray ~ Location/Facility + Geno_Status, data = meta_tank, permutations = 9999)

#  Convert the result to a data frame
# result_perm.f1 <- as.data.frame(perm.f1)

#  Save as CSV
# write.csv(result_perm.f1, "Figures/25_AM/S4FishBray_hier.csv", row.names = TRUE)


perm.f2 <- adonis2(dist.f.bray ~ Geno_Status, data= meta_tank, strata = meta_tank$Facility, nperm = 9999)

#  Convert the result to a data frame
 # result_perm.f2 <- as.data.frame(perm.f2)

#  Save as CSV
 # write.csv(result_perm.f2, "Figures/25_AM/S4_FishBray_cons.csv", row.names = TRUE)
```

##### Supplementary Table 4 - Unweighted UniFrac

Repeat with unweighted UniFrac distance values.

```
perm.f3 <- adonis2(dist.f.uni ~ Location/Facility + Geno_Status, data = meta_tank, permutations = 9999)

#  Convert the result to a data frame
# result_perm.f3 <- as.data.frame(perm.f3)

#  Save as CSV
# write.csv(result_perm.f3, "Figures/25_AM/S4FishUni_hier.csv", row.names = TRUE)

perm.f4 <- adonis2(dist.f.uni ~ Geno_Status, data= meta_tank, strata = meta_tank$Facility, nperm = 9999)

#  Convert the result to a data frame
# result_perm.f4 <- as.data.frame(perm.f4)

#  Save as CSV
# write.csv(result_perm.f4, "Figures/25_AM/S4FishUni_cons.csv", row.names = TRUE)
```

Create stacked table using PERMANOVA outputs.

```
stacked_permanova_fish <- bind_rows(
  # Bray-Curtis tables
  perm.f1 %>% mutate(Analysis = "BC_Hierarchical"),
  perm.f2 %>% mutate(Analysis = "BC_Constrained"),
  # UniFrac tables
  perm.f3 %>% mutate(Analysis = "UF_Hierarchical"),
  perm.f4 %>% mutate(Analysis = "UF_Constrained")
) %>%
  rename_with(~ gsub("\\.\\.\\.\\d+$", "", .x)) %>%
  select(-Analysis) %>%
  kbl(caption = "Fish Gut PERMANOVA Results") %>%
  kable_classic(full_width = F, html_font = "Cambria") %>%
  # Bray-Curtis section
  pack_rows("Bray-Curtis Dissimilarity", 
            1, nrow(perm.w1) + nrow(perm.w2), 
            label_row_css = "background-color: #d0d0d0; font-weight: bold;") %>%
  pack_rows("A) Hierarchical PERMANOVA: Location, Facility, and Genotype Effects", 
            1, nrow(perm.w1), 
            label_row_css = "background-color: #f0f0f0;") %>%
  pack_rows("B) Constrained PERMANOVA: Genotype Effect within Facilities", 
            nrow(perm.w1) + 1, nrow(perm.w1) + nrow(perm.w2), 
            label_row_css = "background-color: #f0f0f0;") %>%
  # UniFrac section
  pack_rows("Unweighted UniFrac Distance", 
            nrow(perm.w1) + nrow(perm.w2) + 1, 
            nrow(perm.w1) + nrow(perm.w2) + nrow(perm.w3) + nrow(perm.w4), 
            label_row_css = "background-color: #d0d0d0; font-weight: bold;") %>%
  pack_rows("A) Hierarchical PERMANOVA: Location, Facility, and Genotype Effects", 
            nrow(perm.w1) + nrow(perm.w2) + 1, 
            nrow(perm.w1) + nrow(perm.w2) + nrow(perm.w3), 
            label_row_css = "background-color: #f0f0f0;") %>%
  pack_rows("B) Constrained PERMANOVA: Genotype Effect within Facilities", 
            nrow(perm.w1) + nrow(perm.w2) + nrow(perm.w3) + 1, 
            nrow(perm.w1) + nrow(perm.w2) + nrow(perm.w3) + nrow(perm.w4), 
            label_row_css = "background-color: #f0f0f0;")

# Display the table
stacked_permanova_fish
```

Fish Gut PERMANOVA Results

|  | Df | SumOfSqs | R2 | F | Pr(>F) |
| --- | --- | --- | --- | --- | --- |
| **Bray-Curtis Dissimilarity** | | | | | |
| **A) Hierarchical PERMANOVA: Location, Facility, and Genotype Effects** | | | | | |
| Location…1 | 1 | 3.7820828 | 0.3232443 | 25.654746 | 0.0001 |
| Geno\_Status…2 | 1 | 0.5298133 | 0.0452817 | 3.593847 | 0.0047 |
| Location:Facility…3 | 3 | 1.9338634 | 0.1652820 | 4.372615 | 0.0001 |
| Residual…4 | 37 | 5.4546268 | 0.4661920 | NA | NA |
| Total…5 | 42 | 11.7003864 | 1.0000000 | NA | NA |
| **B) Constrained PERMANOVA: Genotype Effect within Facilities** | | | | | |
| Geno\_Status…6 | 1 | 2.3796437 | 0.2033816 | 10.467556 | 0.3440 |
| Residual…7 | 41 | 9.3207426 | 0.7966184 | NA | NA |
| Total…8 | 42 | 11.7003864 | 1.0000000 | NA | NA |
| **Unweighted UniFrac Distance** | | | | | |
| **A) Hierarchical PERMANOVA: Location, Facility, and Genotype Effects** | | | | | |
| Location…9 | 1 | 2.1883681 | 0.2011022 | 12.606104 | 0.0001 |
| Geno\_Status…10 | 1 | 0.6749284 | 0.0620232 | 3.887928 | 0.0001 |
| Location:Facility…11 | 3 | 1.5955238 | 0.1466222 | 3.063674 | 0.0001 |
| Residual…12 | 37 | 6.4230485 | 0.5902523 | NA | NA |
| Total…13 | 42 | 10.8818689 | 1.0000000 | NA | NA |
| **B) Constrained PERMANOVA: Genotype Effect within Facilities** | | | | | |
| Geno\_Status…14 | 1 | 1.8739477 | 0.1722083 | 8.529366 | 0.4430 |
| Residual…15 | 41 | 9.0079212 | 0.8277917 | NA | NA |
| Total…16 | 42 | 10.8818689 | 1.0000000 | NA | NA |

```
# Save as PNG 
# stacked_permanova_fish %>%
#   save_kable("Figures/25_AM/Supp3_PERM_Fish.png",
#              zoom = 3,
#              vwidth = 2000)
```

### Beta-dispersion test

```
# Bray-Curtis
bd_fish_bc <- betadisper(dist.f.bray, meta_tank$Facility)
permutest(bd_fish_bc, permutations = 9999)
```

```
## 
## Permutation test for homogeneity of multivariate dispersions
## Permutation: free
## Number of permutations: 9999
## 
## Response: Distances
##           Df  Sum Sq  Mean Sq      F N.Perm Pr(>F)  
## Groups     4 0.23869 0.059673 3.5365   9999 0.0225 *
## Residuals 38 0.64118 0.016873                       
## ---
## Signif. codes:  0 '***' 0.001 '**' 0.01 '*' 0.05 '.' 0.1 ' ' 1
```

```
permutest(bd_fish_bc, pairwise = TRUE, permutations = 9999)
```

```
## 
## Permutation test for homogeneity of multivariate dispersions
## Permutation: free
## Number of permutations: 9999
## 
## Response: Distances
##           Df  Sum Sq  Mean Sq      F N.Perm Pr(>F)  
## Groups     4 0.23869 0.059673 3.5365   9999 0.0207 *
## Residuals 38 0.64118 0.016873                       
## ---
## Signif. codes:  0 '***' 0.001 '**' 0.01 '*' 0.05 '.' 0.1 ' ' 1
## 
## Pairwise comparisons:
## (Observed p-value below diagonal, permuted p-value above diagonal)
##           Ore1 Ore2     Nor1    Nor2A  Nor2B
## Ore1                0.044900 0.122600 0.6143
## Ore2                                        
## Nor1  0.043424               0.758300 0.0319
## Nor2A 0.124839      0.750086          0.1166
## Nor2B 0.616365      0.031652 0.118507
```

```
# unweighted UniFrac
bd_fish_uni <- betadisper(dist.f.uni, meta_tank$Facility)
permutest(bd_fish_uni, permutations = 9999)
```

```
## 
## Permutation test for homogeneity of multivariate dispersions
## Permutation: free
## Number of permutations: 9999
## 
## Response: Distances
##           Df  Sum Sq  Mean Sq      F N.Perm Pr(>F)    
## Groups     4 0.16633 0.041583 7.8067   9999  7e-04 ***
## Residuals 38 0.20241 0.005327                         
## ---
## Signif. codes:  0 '***' 0.001 '**' 0.01 '*' 0.05 '.' 0.1 ' ' 1
```

```
permutest(bd_fish_uni, pairwise = TRUE, permutations = 9999)
```

```
## 
## Permutation test for homogeneity of multivariate dispersions
## Permutation: free
## Number of permutations: 9999
## 
## Response: Distances
##           Df  Sum Sq  Mean Sq      F N.Perm Pr(>F)    
## Groups     4 0.16633 0.041583 7.8067   9999  7e-04 ***
## Residuals 38 0.20241 0.005327                         
## ---
## Signif. codes:  0 '***' 0.001 '**' 0.01 '*' 0.05 '.' 0.1 ' ' 1
## 
## Pairwise comparisons:
## (Observed p-value below diagonal, permuted p-value above diagonal)
##           Ore1 Ore2     Nor1    Nor2A  Nor2B
## Ore1                0.068900 0.801500 0.6250
## Ore2                                        
## Nor1  0.070837               0.092400 0.3266
## Nor2A 0.797331      0.103905          0.8473
## Nor2B 0.651325      0.310469 0.834058
```

### Pairwise PERMANOVA

Construct pairwise PERMANOVA models for facilities using Bray-Curtis
and unweighted UniFrac distance values. Results are reported in the
manuscript text, without an accompanying figure.

```
# Bray-Curtis
set.seed(100)
pairwise.f.bray <- pairwise.adonis(dist.f.bray, factors = meta_tank$Facility, sim.function = "vegdist", sim.method = "bray", p.adjust.m = "BH", perm = 9999)

# Unweighted UniFrac
set.seed(100)
pairwise.f.uni <- pairwise.adonis(dist.f.uni, factors = meta_tank$Facility, sim.function = "vegdist", sim.method = "unifrac", p.adjust.m = "BH", reduce = NULL, perm = 9999)
```

#### Ore2 Exclusion-based sensitivity analysis - PERMANOVA and pairwise PERMANOVA

```
# create new metadata df with no Ore2
meta_tank_noOre2 <- subset(meta_tank, Facility != "Ore2")

# match rownames
rownames(meta_tank_noOre2) <- meta_tank_noOre2$TankID
keep <- rownames(meta_tank_noOre2)

# Calculate distances 
dist.f.bray.noOre2 <- as.dist(as.matrix(dist.f.bray)[keep, keep])
dist.f.uni.noOre2 <- as.dist(as.matrix(dist.f.uni)[keep, keep])

# Rerun PERMANOVAs - Bray-Curtis
perm.f1.noOre2 <- adonis2(dist.f.bray.noOre2 ~ Location/Facility + Geno_Status, data = meta_tank_noOre2, permutations = 9999)
perm.f2.noOre2 <- adonis2(dist.f.bray.noOre2 ~ Geno_Status, data = meta_tank_noOre2, strata = meta_tank_noOre2$Facility, permutations = 9999)

# Unweighted UniFrac
perm.f3.noOre2 <- adonis2(dist.f.uni.noOre2 ~ Location/Facility + Geno_Status, data = meta_tank_noOre2, permutations = 9999)
perm.f4.noOre2 <- adonis2(dist.f.uni.noOre2 ~ Geno_Status, data = meta_tank_noOre2, strata = meta_tank_noOre2$Facility, permutations = 9999)


# Rerun pairwise PERMANOVAs
pairwise.f.bray.noOre2 <- pairwise.adonis(dist.f.bray.noOre2, factors = meta_tank_noOre2$Facility, sim.function = "vegdist", sim.method = "bray", p.adjust.m = "BH", reduce = NULL, perm = 9999)

pairwise.f.uni.noOre2 <- pairwise.adonis(dist.f.uni.noOre2, factors = meta_tank_noOre2$Facility, sim.function = "vegdist", sim.method = "unifrac", p.adjust.m = "BH", reduce = NULL, perm = 9999)
```

```
#  Convert the results to a data frame
result_p1_noOre2 <- as.data.frame(perm.f1.noOre2)
result_p2_noOre2 <- as.data.frame(perm.f2.noOre2)
result_p3_noOre2 <- as.data.frame(perm.f3.noOre2)
result_p4_noOre2 <- as.data.frame(perm.f4.noOre2)
result_pw1_noOre2 <- as.data.frame(pairwise.f.bray.noOre2)
result_pw2_noOre2 <- as.data.frame(pairwise.f.uni.noOre2)

#  Save as CSV
write.csv(result_p1_noOre2, "Figures/25_AM/Supp2_perm1.csv", row.names = TRUE)
write.csv(result_p2_noOre2, "Figures/25_AM/Supp2_perm2.csv", row.names = TRUE)
write.csv(result_p3_noOre2, "Figures/25_AM/Supp2_perm3.csv", row.names = TRUE)
write.csv(result_p4_noOre2, "Figures/25_AM/Supp2_perm4.csv", row.names = TRUE)
write.csv(result_pw1_noOre2, "Figures/25_AM/Supp2_pw1.csv", row.names = TRUE)
write.csv(result_pw2_noOre2, "Figures/25_AM/Supp2_pw2.csv", row.names = TRUE)
```

## Paired Fish & Water Samples

### PERMANOVA

```
# rarefy water microbiome data to same level as fish
ps_rare.w.matched <- phyloseq::rarefy_even_depth(ps.22.w, 
                                                  sample.size = 1171,
                                                  rngseed = 123, 
                                                  replace = FALSE)

# drop phylogenetic tree from both to merge
ps_tank_notree <- phyloseq(otu_table(ps_tank), 
                            sample_data(ps_tank),
                            tax_table(ps_tank))


ps_rare.w_notree <- phyloseq(otu_table(ps_rare.w.matched), 
                              sample_data(ps_rare.w.matched),
                              tax_table(ps_rare.w.matched))

# ensure Sample_ID column matches those in water ps
sample_data(ps_tank_notree)$Sample_ID <- sample_names(ps_tank_notree)

# merge rarefied & averaged fish ps object with new rarefied water ps
ps_rare.all <- merge_phyloseq(ps_tank_notree, ps_rare.w_notree)

# Calculate Bray-Curtis on combined object

dist_all_bray <- phyloseq::distance(ps_rare.all, method = "bray")

# create new metadata file
meta_all <- data.frame(sample_data(ps_rare.all), stringsAsFactors = FALSE)

meta_all$Sample_Type <- as.factor(meta_all$Sample_Type)
meta_all$Facility <- as.factor(meta_all$Facility)
meta_all <- meta_all[labels(dist_all_bray), ]


# Additive 
perm_all <- adonis2(dist_all_bray ~ Sample_Type + Facility, data = meta_all, permutations = 9999)

# Interaction
perm_all_interact <- adonis2(dist_all_bray ~ Sample_Type * Facility,
                              data = meta_all,
                              permutations = 9999)

# Betadisper for combined analysis
bd_all_type <- betadisper(dist_all_bray, meta_all$Sample_Type)
permutest(bd_all_type, permutations = 9999)
```

```
## 
## Permutation test for homogeneity of multivariate dispersions
## Permutation: free
## Number of permutations: 9999
## 
## Response: Distances
##           Df  Sum Sq Mean Sq      F N.Perm Pr(>F)  
## Groups     1 0.12729 0.12729 6.9708   9999 0.0105 *
## Residuals 91 1.66167 0.01826                       
## ---
## Signif. codes:  0 '***' 0.001 '**' 0.01 '*' 0.05 '.' 0.1 ' ' 1
```

```
bd_all_facility <- betadisper(dist_all_bray, meta_all$Facility)
permutest(bd_all_facility, permutations = 9999)
```

```
## 
## Permutation test for homogeneity of multivariate dispersions
## Permutation: free
## Number of permutations: 9999
## 
## Response: Distances
##           Df  Sum Sq  Mean Sq      F N.Perm Pr(>F)    
## Groups     4 0.39721 0.099303 5.6211   9999  7e-04 ***
## Residuals 88 1.55462 0.017666                         
## ---
## Signif. codes:  0 '***' 0.001 '**' 0.01 '*' 0.05 '.' 0.1 ' ' 1
```

## Pairwise PERMANOVA Combined

```
meta_all$Type_Facility <- paste(meta_all$Sample_Type, meta_all$Facility, sep = "_")

# Pairwise PERMANOVA on combined grouping
pairwise_all <- pairwise.adonis2(dist_all_bray ~ Type_Facility,
                                  data = meta_all,
                                  nperm = 9999,
                                  p.adjust.m = "BH")

# Extract and apply BH correction
pvals_all <- sapply(pairwise_all[-1], function(x) x["Type_Facility", "Pr(>F)"])
pvals_all_adj <- p.adjust(pvals_all, method = "BH")

# View results
data.frame(
  pair = names(pvals_all),
  p_raw = pvals_all,
  p_adj = round(pvals_all_adj, 4)
) %>% arrange(p_adj)
```

```
##                                                  pair p_raw  p_adj
## fish_Ore1_vs_fish_Nor2B       fish_Ore1_vs_fish_Nor2B 0.001 0.0018
## fish_Ore1_vs_fish_Nor2A       fish_Ore1_vs_fish_Nor2A 0.001 0.0018
## fish_Ore1_vs_fish_Nor1         fish_Ore1_vs_fish_Nor1 0.001 0.0018
## fish_Ore1_vs_water_Ore1       fish_Ore1_vs_water_Ore1 0.001 0.0018
## fish_Ore1_vs_water_Ore2       fish_Ore1_vs_water_Ore2 0.001 0.0018
## fish_Ore1_vs_water_Nor2A     fish_Ore1_vs_water_Nor2A 0.001 0.0018
## fish_Ore1_vs_water_Nor1       fish_Ore1_vs_water_Nor1 0.001 0.0018
## fish_Nor2B_vs_fish_Nor1       fish_Nor2B_vs_fish_Nor1 0.001 0.0018
## fish_Nor2B_vs_water_Ore1     fish_Nor2B_vs_water_Ore1 0.001 0.0018
## fish_Nor2A_vs_fish_Nor1       fish_Nor2A_vs_fish_Nor1 0.001 0.0018
## fish_Nor2A_vs_water_Ore1     fish_Nor2A_vs_water_Ore1 0.001 0.0018
## fish_Nor2A_vs_water_Ore2     fish_Nor2A_vs_water_Ore2 0.001 0.0018
## fish_Nor2A_vs_water_Nor2A   fish_Nor2A_vs_water_Nor2A 0.001 0.0018
## fish_Nor2A_vs_water_Nor1     fish_Nor2A_vs_water_Nor1 0.001 0.0018
## fish_Nor1_vs_water_Ore1       fish_Nor1_vs_water_Ore1 0.001 0.0018
## fish_Nor1_vs_water_Ore2       fish_Nor1_vs_water_Ore2 0.001 0.0018
## fish_Nor1_vs_water_Nor2A     fish_Nor1_vs_water_Nor2A 0.001 0.0018
## fish_Nor1_vs_water_Nor1       fish_Nor1_vs_water_Nor1 0.001 0.0018
## water_Ore1_vs_water_Ore2     water_Ore1_vs_water_Ore2 0.001 0.0018
## water_Ore1_vs_water_Nor2A   water_Ore1_vs_water_Nor2A 0.001 0.0018
## water_Ore1_vs_water_Nor2B   water_Ore1_vs_water_Nor2B 0.001 0.0018
## water_Ore1_vs_water_Nor1     water_Ore1_vs_water_Nor1 0.001 0.0018
## water_Ore2_vs_water_Nor2A   water_Ore2_vs_water_Nor2A 0.001 0.0018
## water_Ore2_vs_water_Nor1     water_Ore2_vs_water_Nor1 0.001 0.0018
## water_Nor2A_vs_water_Nor1   water_Nor2A_vs_water_Nor1 0.001 0.0018
## fish_Nor2B_vs_water_Ore2     fish_Nor2B_vs_water_Ore2 0.002 0.0035
## fish_Ore1_vs_water_Nor2B     fish_Ore1_vs_water_Nor2B 0.003 0.0050
## fish_Nor2B_vs_water_Nor2A   fish_Nor2B_vs_water_Nor2A 0.005 0.0075
## fish_Nor2B_vs_water_Nor1     fish_Nor2B_vs_water_Nor1 0.005 0.0075
## water_Ore2_vs_water_Nor2B   water_Ore2_vs_water_Nor2B 0.005 0.0075
## fish_Nor2A_vs_water_Nor2B   fish_Nor2A_vs_water_Nor2B 0.006 0.0087
## fish_Nor1_vs_water_Nor2B     fish_Nor1_vs_water_Nor2B 0.007 0.0098
## water_Nor2B_vs_water_Nor1   water_Nor2B_vs_water_Nor1 0.009 0.0123
## fish_Nor2B_vs_fish_Nor2A     fish_Nor2B_vs_fish_Nor2A 0.011 0.0141
## water_Nor2A_vs_water_Nor2B water_Nor2A_vs_water_Nor2B 0.011 0.0141
## fish_Nor2B_vs_water_Nor2B   fish_Nor2B_vs_water_Nor2B 0.029 0.0363
## fish_Ore2_vs_water_Ore1       fish_Ore2_vs_water_Ore1 0.090 0.1095
## fish_Nor1_vs_fish_Ore2         fish_Nor1_vs_fish_Ore2 0.097 0.1149
## fish_Ore2_vs_water_Nor2A     fish_Ore2_vs_water_Nor2A 0.132 0.1523
## fish_Ore2_vs_water_Nor1       fish_Ore2_vs_water_Nor1 0.149 0.1676
## fish_Nor2B_vs_fish_Ore2       fish_Nor2B_vs_fish_Ore2 0.200 0.2195
## fish_Nor2A_vs_fish_Ore2       fish_Nor2A_vs_fish_Ore2 0.211 0.2261
## fish_Ore1_vs_fish_Ore2         fish_Ore1_vs_fish_Ore2 0.218 0.2281
## fish_Ore2_vs_water_Nor2B     fish_Ore2_vs_water_Nor2B 0.250 0.2557
## fish_Ore2_vs_water_Ore2       fish_Ore2_vs_water_Ore2 0.310 0.3100
```

```
# Filter to only fish vs water comparisons within same facility
fish_water_full <- lapply(names(pairwise_all[-1]), function(x) {
  pair <- pairwise_all[[x]]
  data.frame(
    pair = x,
    Df = pair["Type_Facility", "Df"],
    SumOfSqs = round(pair["Type_Facility", "SumOfSqs"], 4),
    R2 = round(pair["Type_Facility", "R2"], 4),
    F = round(pair["Type_Facility", "F"], 4),
    p_raw = pair["Type_Facility", "Pr(>F)"]
  )
}) %>%
  bind_rows() %>%
  mutate(p_adj = round(p.adjust(p_raw, method = "BH"), 4)) %>%
  # Filter to within-facility fish vs water comparisons only
  filter(grepl("fish.*water|water.*fish", pair)) %>%
  filter(sapply(strsplit(pair, "_vs_"), function(x) {
    fac1 <- gsub("fish_|water_", "", x[1])
    fac2 <- gsub("fish_|water_", "", x[2])
    fac1 == fac2
  })) %>%
  # Clean up pair names
  mutate(pair = gsub("fish_|_vs_water_", " vs. Water ", pair)) %>%
  rename(Comparison = pair) %>%
  arrange(p_adj)

fish_water_full
```

```
##                         Comparison Df SumOfSqs     R2       F p_raw  p_adj
## 1    vs. Water Ore1 vs. Water Ore1  1   2.9394 0.2860 16.4210 0.001 0.0018
## 2  vs. Water Nor2A vs. Water Nor2A  1   1.8885 0.4257  8.8954 0.001 0.0018
## 3    vs. Water Nor1 vs. Water Nor1  1   1.8481 0.3988  9.2858 0.001 0.0018
## 4  vs. Water Nor2B vs. Water Nor2B  1   1.3002 0.6782 10.5354 0.029 0.0363
## 5    vs. Water Ore2 vs. Water Ore2  1   0.1410 0.0797  0.9528 0.310 0.3100
```

```
# Subset to exclude Ore2
meta_all_noOre2 <- meta_all %>% filter(Facility != "Ore2")
keep_noOre2 <- rownames(meta_all_noOre2)

# Subset distance matrix
dist_all_bray_noOre2 <- as.dist(as.matrix(dist_all_bray)[keep_noOre2, keep_noOre2])

# Rerun additive model
perm_all_noOre2 <- adonis2(dist_all_bray_noOre2 ~ Sample_Type + Facility,
                            data = meta_all_noOre2,
                            permutations = 9999)

# Rerun interaction model
perm_all_interact_noOre2 <- adonis2(dist_all_bray_noOre2 ~ Sample_Type * Facility,
                                     data = meta_all_noOre2,
                                     permutations = 9999)

# Rerun pairwise
pairwise_all_noOre2 <- pairwise.adonis2(dist_all_bray_noOre2 ~ Type_Facility,
                                         data = meta_all_noOre2,
                                         nperm = 9999,
                                         p.adjust.m = "BH")

# Extract full statistics from pairwise results excluding Ore2
fish_water_full_noOre2 <- lapply(names(pairwise_all_noOre2[-1]), function(x) {
  pair <- pairwise_all_noOre2[[x]]
  data.frame(
    pair = x,
    SumOfSqs = round(pair["Type_Facility", "SumOfSqs"], 4),
    R2 = round(pair["Type_Facility", "R2"], 4),
    F = round(pair["Type_Facility", "F"], 4),
    p_raw = pair["Type_Facility", "Pr(>F)"]
  )
}) %>%
  bind_rows() %>%
  mutate(p_adj = round(p.adjust(p_raw, method = "BH"), 4)) %>%
  # Filter to within-facility fish vs water comparisons only
  filter(grepl("fish.*water|water.*fish", pair)) %>%
  filter(sapply(strsplit(pair, "_vs_"), function(x) {
    fac1 <- gsub("fish_|water_", "", x[1])
    fac2 <- gsub("fish_|water_", "", x[2])
    fac1 == fac2
  })) %>%
  mutate(pair = gsub("fish_|_vs_water_", " vs. Water ", pair)) %>%
  rename(Comparison = pair) %>%
  arrange(p_adj)

fish_water_full_noOre2
```

```
##                         Comparison SumOfSqs     R2       F p_raw  p_adj
## 1    vs. Water Ore1 vs. Water Ore1   2.9394 0.2860 16.4210 0.001 0.0016
## 2    vs. Water Nor1 vs. Water Nor1   1.8481 0.3988  9.2858 0.001 0.0016
## 3  vs. Water Nor2A vs. Water Nor2A   1.8885 0.4257  8.8954 0.002 0.0028
## 4  vs. Water Nor2B vs. Water Nor2B   1.3002 0.6782 10.5354 0.023 0.0230
```

### Pairwise Beta-Diversity for Fish and Tank Water Across Facilities

Rarefy combined fish gut and water microbiome data. Calculate
Bray-Curtis distance values for combined, rarefied dataset.

```
# Calculate Bray-Curtis distances
bray.m = phyloseq::distance(ps_rare.all, method = "bray") %>%
  as.matrix() %>%
  as.data.frame() %>%
  rownames_to_column("Var1") %>%
  pivot_longer(cols = c(everything(), -Var1), 
               names_to = "Var2", 
               values_to = "value") %>%
  filter(as.character(Var1) != as.character(Var2))

# Get sample data from merged object
sd = data.frame(sample_data(ps_rare.all)) %>%
  select(all_of(c('Sample_ID', "Sample_Type", 'Facility', 'ShelfID', 'TankID'))) %>%
  mutate_if(is.factor, as.character)

# Combine BC distances with sample data
colnames(sd) = c("Var1", "Type1", "Fac1", "Shelf1", "Tank1")
bray.sd = left_join(bray.m, sd, by = "Var1", relationship = "many-to-many")
colnames(sd) = c("Var2", "Type2", "Fac2", "Shelf2", "Tank2")
bray.sd = left_join(bray.sd, sd, by = "Var2", relationship = "many-to-many")
```

```
make_same <- function(x) {
  if (x$Type1 == x$Type2) {
    "fish"
  }
  else if (x$Tank1 == x$Tank2) {
    "same tank"
  }
  else if (x$Fac1 == x$Fac2) {
    "same facility"
  }
  else {
    "different facility"
  
  }
}
```

##### Ore1

Identify and group fish and water samples that are either 1) fish to
fish pairwise comparisons, 2) fish to water from the same tank, 3) fish
and water from other tanks but within the same facility, or 4) fish and
water from tanks in different facilities.

```
# Recode fish and water samples from other facilities
bray.ore1 = bray.sd %>%
  mutate(Fac2 = recode(Fac2, 'Ore2' = 'Other', 'Nor1' = 'Other', 'Nor2A' = 'Other', 'Nor2B' = 'Other')) %>%
  unite("Type2", Type2:Fac2, remove = FALSE) %>%
  unite("Type1", Type1:Fac1, remove = FALSE) %>%
  filter(Type1 == "fish_Ore1") %>%
  filter(Type2 != "fish_Other") %>%
mutate(same_tank = Tank1 == Tank2)

bray.ore1$x_axis <- vector("character", nrow(bray.ore1))
for (i in 1:nrow(bray.ore1)) {
    bray.ore1[i,]$x_axis <- make_same(bray.ore1[i,])
    }

order.ore1 = c("fish", "same tank", "same facility", "different facility")
bray.ore1$x_axis <- 
  factor(bray.ore1$x_axis, levels = order.ore1, ordered = TRUE)
```

Create pair-wise beta-diversity plot for Ore1 data using the fish
vs. tank water location groupings.

```
plot.ore1 = ggplot(bray.ore1, aes(x = x_axis, y = value)) +
  theme_bw(base_size = 10) +
  geom_point(aes(color = ifelse(Type1 == Type2, "#D43f3aff", "black"))) +
  geom_boxplot(aes(color = ifelse(Type1 == Type2, "#D43f3aff", "black"))) +
  scale_color_identity() +
  ylim(0, 1.25) +
  theme(axis.text.x = element_text(angle = 15, hjust = 0.5, vjust = 0.6)) +
  theme(axis.title.x = element_blank()) +
  labs(title = "Ore1", y = "Bray-Curtis dissimilarity")
```

##### Ore2

Identitfy all fish and water in Ore2; reclassify samples in other
facilities as ‘Other’.

```
bray.ore2 = bray.sd %>%
  mutate(Fac2 = recode(Fac2, 'Ore1' = 'Other', 'Nor1' = 'Other', 'Nor2A' = 'Other', 'Nor2B' = 'Other')) %>%
  unite("Type2", Type2:Fac2, remove = FALSE) %>%
  unite("Type1", Type1:Fac1, remove = FALSE) %>%
  filter(Type1 == "fish_Ore2") %>%
  filter(Type2 != "fish_Other")%>%
mutate(same_tank = Tank1 == Tank2)

bray.ore2$x_axis <- vector("character", nrow(bray.ore2))
for (i in 1:nrow(bray.ore2)) {
    bray.ore2[i,]$x_axis <- make_same(bray.ore2[i,])
    }

bray.ore2$x_axis <- 
  factor(bray.ore2$x_axis, levels = order.ore1, ordered = TRUE)
```

```
plot.ore2 = ggplot(bray.ore2, aes(x = x_axis, y = value)) +
  theme_bw(base_size = 10) +
  geom_point(aes(color = ifelse(Type1 == Type2, "#Eea236ff", "black"))) +
  geom_boxplot(aes(color = ifelse(Type1 == Type2, "#Eea236ff", "black"))) +
  scale_color_identity() +
  ylim(0, 1.25) +
  theme(axis.text.x = element_text(angle = 15, hjust = 0.5, vjust = 0.6)) +
  theme(axis.title.x = element_blank()) +
  labs(title = "Ore2", y = "")
```

##### Nor1

Identitfy all fish and water in Nor1; reclassify samples in other
facilities as ‘Other’.

```
bray.nor1 = bray.sd %>%
  mutate(Fac2 = recode(Fac2, 'Ore1' = 'Other', 'Ore2' = 'Other', 'Nor2A' = 'Other', 'Nor2B' = 'Other')) %>%
  unite("Type2", Type2:Fac2, remove = FALSE) %>%
  unite("Type1", Type1:Fac1, remove = FALSE) %>%
  filter(Type1 == "fish_Nor1") %>%
  filter(Type2 != "fish_Other")%>%
mutate(same_tank = Tank1 == Tank2)

bray.nor1$x_axis <- vector("character", nrow(bray.nor1))
for (i in 1:nrow(bray.nor1)) {
    bray.nor1[i,]$x_axis <- make_same(bray.nor1[i,])
    }

bray.nor1$x_axis <- 
  factor(bray.nor1$x_axis, levels = order.ore1, ordered = TRUE)
```

```
plot.nor1 = ggplot(bray.nor1, aes(x = x_axis, y = value)) +
  theme_bw(base_size = 10) +
  geom_point(aes(color = ifelse(Type1 == Type2, "#5cb85cff", "black"))) +
  geom_boxplot(aes(color = ifelse(Type1 == Type2, "#5cb85cff", "black"))) +
  scale_color_identity() +
  ylim(0, 1.25) +
  theme(axis.text.x = element_text(angle = 15, hjust = 0.5, vjust = 0.6)) +
  theme(axis.title.x = element_blank()) +
  labs(title = "Nor1", y = "")
```

##### Nor2A

Identitfy all fish and water in Nor2A; reclassify samples in other
facilities as ‘Other’.

```
bray.nor2a = bray.sd %>%
  mutate(Fac2 = recode(Fac2, 'Nor2B' = 'Other', 'Nor1' = 'Other', 'Ore1' = 'Other', 'Ore2' = 'Other')) %>%
  unite("Type2", Type2:Fac2, remove = FALSE) %>%
  unite("Type1", Type1:Fac1, remove = FALSE) %>%
  filter(Type1 == "fish_Nor2A") %>%
  filter(Type2 != "fish_Other")%>%
mutate(same_tank = Tank1 == Tank2)

bray.nor2a$x_axis <- vector("character", nrow(bray.nor2a))
for (i in 1:nrow(bray.nor2a)) {
    bray.nor2a[i,]$x_axis <- make_same(bray.nor2a[i,])
    }

bray.nor2a$x_axis <- 
  factor(bray.nor2a$x_axis, levels = order.ore1, ordered = TRUE)
```

```
plot.nor2a = ggplot(bray.nor2a, aes(x = x_axis, y = value)) +
  theme_bw(base_size = 10) +
  geom_point(aes(color = ifelse(Type1 == Type2, "#46b8daff", "black"))) +
  geom_boxplot(aes(color = ifelse(Type1 == Type2, "#46b8daff", "black"))) +
  scale_color_identity() +
  ylim(0, 1.25) +
  theme(axis.text.x = element_text(angle = 15, hjust = 0.5, vjust = 0.6)) +
  theme(axis.title.x = element_blank()) +
  labs(title = "Nor2A", y = "Bray-Curtis dissimilarity")
```

##### Nor2B

Identitfy all fish and water in Nor2B; reclassify samples in other
facilities as ‘Other’.

```
bray.nor2b = bray.sd %>%
  mutate(Fac2 = recode(Fac2, 'Ore1' = 'Other', 'Nor1' = 'Other', 'Ore2' = 'Other', 'Nor2A' = 'Other')) %>%
  unite("Type2", Type2:Fac2, remove = FALSE) %>%
  unite("Type1", Type1:Fac1, remove = FALSE) %>%
  filter(Type1 == "fish_Nor2B") %>%
  filter(Type2 != "fish_Other")%>%
mutate(same_tank = Tank1 == Tank2)

bray.nor2b$x_axis <- vector("character", nrow(bray.nor2b))
for (i in 1:nrow(bray.nor2b)) {
    bray.nor2b[i,]$x_axis <- make_same(bray.nor2b[i,])
    }

bray.nor2b$x_axis <- 
  factor(bray.nor2b$x_axis, levels = order.ore1, ordered = TRUE)
```

```
plot.nor2b = ggplot(bray.nor2b, aes(x = x_axis, y = value)) +
  theme_bw(base_size = 10) +
  geom_point(aes(color = ifelse(Type1 == Type2, "#9590ff", "black"))) +
  geom_boxplot(aes(color = ifelse(Type1 == Type2, "#9590ff", "black"))) +
  scale_color_identity() +
  ylim(0, 1.25) +
  theme(axis.text.x = element_text(angle = 15, hjust = 0.5, vjust = 0.6)) +
  theme(axis.title.x = element_blank()) +
  labs(title = "Nor2B", y = "")
```

#### Supplementary Figure S1

Create combined figure of all facility pair-wise beta-diversity
plots.

```
all_bdiv.tank <- ggarrange(plot.ore1, plot.nor1, plot.nor2a, plot.nor2b, nrow = 2, ncol = 2)

all_bdiv.tank
```

```
#save 
#ggsave("Figures/25_AM/FigS1_PairBDiv.png", all_bdiv.tank, width = 170, units = ("mm"), bg = "white")
```

# Ordinations - PCoA

## Figure 4 - Water Microbiome Samples

Construct ordinations for water microbiome samples using Bray-Curtis
and unweighted UniFrac distance values.

```
ord.w.bray = ordinate(ps_rare.w, method="PCoA", distance=dist.w.bray)
ord.w.uni = ordinate(ps_rare.w, method="PCoA", distance=dist.w.uni)
```

Create PCoA plots.

```
# Bray Curtis
pcoa.w.bray <- plot_ordination(ps_rare.w, ord.w.bray, color = "Facility") + 
  geom_point() +
  ggtitle("Bray-Curtis") +
  scale_color_manual(values = locuszoom) + 
  theme_bw() + 
  labs(color = "Facility") +
  stat_ellipse(aes(group = Facility), linetype = 1) +
  stat_ellipse(aes(group = Location), linetype = 2, color = "black") +
  annotate("text", x = 0.32, y = -0.24, label = "Norway", color = "black", size = 4) +
  annotate("text", x = -0.32, y = 0.32, label = "Oregon", color = "black", size = 4)

# Unweighted UniFrac
pcoa.w.uni <- plot_ordination(ps_rare.w, ord.w.uni, color = "Facility") + 
  geom_point() +
  ggtitle("Unweighted UniFrac") +
  scale_color_manual(values = locuszoom) + 
  theme_bw() + 
  labs(color = "Facility") +
  stat_ellipse(aes(group = Facility), linetype = 1) +
  stat_ellipse(aes(group = Location), linetype = 2, color = "black") +
  annotate("text", x = 0.4, y = -0.18, label = "Norway", color = "black", size = 4) +
  annotate("text", x = -0.29, y = 0.14, label = "Oregon", color = "black", size = 4)
```

Arrange plots into combined figure.

```
pcoa_water <- ggarrange(pcoa.w.bray, pcoa.w.uni, ncol = 2, nrow= 1, common.legend = TRUE, legend = "right", labels = "AUTO")
pcoa_water
```

```
#save 
ggsave("Figures/25_AM/Fig4_PCoAWater.png", pcoa_water, width = 200, units = ("mm"))
```

## Figure 7 - Fish Gut Microbiome Samples

Repeat ordinations for fish gut microbiome samples.

```
ord.f.bray = ordinate(ps_tank, method="PCoA", distance=dist.f.bray)
ord.f.uni = ordinate(ps_tank, method="PCoA", distance=dist.f.uni)
```

Create PCoA plots.

```
# Bray Curtis
pcoa.f.bray <- plot_ordination(ps_tank, ord.f.bray, color = "Facility") + 
  geom_point() +
  ggtitle("Bray-Curtis") +
  scale_color_manual(values = locuszoom) + 
  theme_bw() + 
  labs(color = "Facility") +
  stat_ellipse(aes(group = Facility), linetype = 1) +
  stat_ellipse(aes(group = Location), linetype = 2, color = "black") +
  annotate("text", x = 0.25, y = 0.52, label = "Norway", color = "black", size = 4) +
  annotate("text", x = -0.3, y = -0.3, label = "Oregon", color = "black", size = 4)

# Unweighted UniFrac
pcoa.f.uni <- plot_ordination(ps_tank, ord.f.uni, color = "Facility") + 
  geom_point() +
  ggtitle("Unweighted UniFrac") +
  theme_bw()+
  scale_color_manual(values = locuszoom) + 
  labs(color = "Facility") +
  stat_ellipse(aes(group = Facility), linetype = 1) +
  stat_ellipse(aes(group = Location), linetype = 2, color = "black") +
  annotate("text", x = 0.3, y = -0.32, label = "Norway", color = "black", size = 4) +
  annotate("text", x = -0.25, y = -0.28, label = "Oregon", color = "black", size = 4)
```

Combine PCoA plots into single figure.

```
pcoa_fish <- ggarrange(pcoa.f.bray, pcoa.f.uni, common.legend = TRUE, legend = "right", labels = "AUTO")
pcoa_fish
```

```
#save 
# ggsave("Figures/25_AM/Fig7_PCoAFish.png", pcoa_fish, width = 200, units = ("mm"))
```

## Figure 8 - Combined Ordination

```
ord_all_bray = ordinate(ps_rare.all, method="PCoA", distance=dist_all_bray)
```

```
pcoa.combo <- plot_ordination(ps_rare.all, ord_all_bray, color = "Facility", shape = "Sample_Type") + 
  geom_point(size = 2) +
  ggtitle("Bray-Curtis") +
  theme_bw()+
  scale_color_manual(values = locuszoom) + 
  scale_shape_manual(values = c("fish" = 16, "water" = 17)) +
  stat_ellipse(aes(group = Facility), linetype = 1) 

pcoa.combo
```

```
#save 
#ggsave("Figures/25_AM/PCoACombined.png", pcoa.combo, width = 170, units = ("mm"))
```

# Relative Abundance

Modify code from the package::fantaxtic to adjust colors in resulting
figures. Find original code here: https://github.com/gmteunisse/fantaxtic.

```
# Adjust color palette & theme under ggnested wrapper in fantaxtic package

plot_nested_bar_adj <- function (ps_obj, top_level, nested_level, top_merged_label = "Other", 
    nested_merged_label = "Other <tax>", palette = NULL, base_clr = "#008CF0", 
    merged_clr = "grey90", include_rank = T, na_taxon_label = "<tax> (<rank>)", 
    asv_as_id = F, duplicate_taxon_label = "<tax> <id>", relative_abundances = T, 
    sample_order = NULL, ...) 
{
    pal <- taxon_colours(ps_obj, tax_level = top_level, merged_label = top_merged_label, 
        merged_clr = merged_clr, palette = palette, base_clr = base_clr)
    ps_tmp <- ps_obj %>% name_na_taxa(include_rank = include_rank, 
        na_label = na_taxon_label)
    ps_tmp <- ps_tmp %>% label_duplicate_taxa(tax_level = nested_level, 
        asv_as_id = asv_as_id, duplicate_label = duplicate_taxon_label)
    psdf <- psmelt(ps_tmp)
    psdf <- move_label(psdf = psdf, col_name = top_level, label = top_merged_label, 
        pos = 0)
    psdf <- move_nested_labels(psdf, top_level = top_level, nested_level = nested_level, 
        top_merged_label = top_merged_label, nested_label = gsub("<tax>", 
            "", nested_merged_label), pos = Inf)
    if (!is.null(sample_order)) {
        if (all(sample_order %in% unique(psdf$Sample))) {
            psdf <- psdf %>% mutate(Sample = factor(Sample, levels = sample_order))
        }
        else {
            stop("Error: not all(sample_order %in% sample_names(ps_obj)).")
        }
    }
  
  
    p <- ggnested(psdf, aes_string(main_group = top_level, sub_group = nested_level, 
        x = "Abundance", y = "Sample"), ..., main_palette = locuszoom_grey, gradient_type = "both", min_l = 0.3,
         max_l = 0.99) + 
        scale_x_continuous(expand = c(0, 0)) + theme_nested(theme_classic) + 
        theme(axis.text.x = element_text(hjust = 1, vjust = 0.5, 
            angle = 90), axis.text.y=element_blank())
    if (relative_abundances) {
        p <- p + geom_col(position = position_fill())
    }
    else {
        p <- p + geom_col()
    }
    return(p)
}
```

## Figure 3 - Water Microbiome Samples

Identify the top 4 most abundant genera within the top 5 most
abundant phyla in water microbiome samples and plot results. The number
of phyla and genera to include was dictated by what produced robust but
readable figures.

```
## Genus nested in phylum

top_nested <- nested_top_taxa(ps_rare.w,
                              top_tax_level = "Phylum",
                              nested_tax_level = "Genus",
                              n_top_taxa = 5, 
                              n_nested_taxa = 4)

# plot
w.ra <- plot_nested_bar_adj(top_nested$ps_obj,
                top_level = "Phylum",
                nested_level = "Genus",
                nested_merged_label = "NA and other <tax>",
                legend_title = "Top phyla and genera") +
  facet_grid(Facility ~ .,
             scales = "free", space = "free_y") +
  theme(legend.key.size = unit(10, "points"),
        legend.position = "right",
        axis.text.y=element_blank(),
        axis.ticks.y=element_blank(),
        panel.spacing.x = unit(0, "pt"),
        panel.border = element_rect(fill = NA, color = "grey2", linetype = "solid",  size = 1)) +
  guides(fill = guide_legend(ncol = 1)) +
  xlab("Relative Abundance")

w.ra
```

```
#save 
# ggsave("Figures/25_AM/Fig3_RA_Water.png", w.ra, height = 225, width = 170, units = ("mm"))
```

```
# Agglomerate to genus level from rarefied water phyloseq
ps_rare.w_genus <- tax_glom(ps_rare.w, taxrank = "Genus")

# Convert to relative abundance
ps_rare.w_genus_rel <- transform_sample_counts(ps_rare.w_genus, function(x) x/sum(x))

# Add facility metadata and average directly to facility level
facility_rel_genus_water <- cbind(
  Facility = meta_rare.w$Facility,
  as.data.frame(otu_table(ps_rare.w_genus_rel))) %>%
  group_by(Facility) %>%
  summarise(across(where(is.numeric), mean, na.rm = TRUE)) %>%
  column_to_rownames("Facility")

# Replace ASV IDs with genus names
tax_genus_water <- as.data.frame(tax_table(ps_rare.w_genus)) %>%
  rownames_to_column("ASV") %>%
  select(ASV, Genus)

colnames(facility_rel_genus_water) <- tax_genus_water$Genus[match(
  colnames(facility_rel_genus_water), tax_genus_water$ASV)]

# Find taxa exceeding 1% mean abundance across all facilities
overall_mean_genus_water <- colMeans(facility_rel_genus_water)
top_taxa_genus_water <- sort(overall_mean_genus_water[overall_mean_genus_water >= 0.01],
                              decreasing = TRUE)
length(top_taxa_genus_water)
```

```
## [1] 22
```

```
top_taxa_genus_water
```

```
##    Cetobacterium      Pseudomonas     Rheinheimera    Acinetobacter 
##       0.13600823       0.09011056       0.08010890       0.06066465 
##        Aeromonas           Vibrio    Psychrobacter       Acidovorax 
##       0.04170467       0.04047382       0.04004341       0.03271511 
##    Perlucidibaca   Flavobacterium          Delftia          Nevskia 
##       0.02859854       0.02741601       0.02482254       0.02411525 
##      Limnobacter       Fluviicola       Shewanella      Plesiomonas 
##       0.02219757       0.02188520       0.02104120       0.01834870 
##        Pelomonas Stenotrophomonas       Paracoccus       Luteimonas 
##       0.01758170       0.01730398       0.01503468       0.01316997 
##    Undibacterium Chryseobacterium 
##       0.01288682       0.01113747
```

```
# Facility specific percentages for top taxa
facility_rel_genus_water %>%
  select(names(top_taxa_genus_water)) %>%
  t() %>%
  as.data.frame() %>%
  mutate(across(everything(), ~ round(. * 100, 1))) %>%
  arrange(desc(rowMeans(.)))
```

```
##                  Ore1 Ore2 Nor1 Nor2A Nor2B
## Cetobacterium    20.2 43.1  4.5   0.0   0.2
## Pseudomonas       1.2  1.0 35.6   6.2   1.1
## Rheinheimera      0.1  0.0  0.1   6.8  33.1
## Acinetobacter     0.0  0.6  5.7   7.4  16.6
## Aeromonas        11.8  5.9  2.0   0.0   1.2
## Vibrio            1.9 11.3  2.6   3.8   0.7
## Psychrobacter    19.3  0.0  0.7   0.0   0.0
## Acidovorax        0.2  0.4  0.4  11.7   3.7
## Perlucidibaca     6.2  0.6  2.0   2.8   2.7
## Flavobacterium    3.2  0.5  2.6   0.6   6.9
## Delftia           0.0  0.0  6.0   6.3   0.0
## Nevskia           1.1  0.2  0.0  10.6   0.2
## Limnobacter       1.0  0.0  0.1   9.2   0.8
## Fluviicola        0.0  0.2  0.0   0.0  10.8
## Shewanella        5.6  3.6  0.4   0.2   0.7
## Plesiomonas       1.7  6.4  1.0   0.0   0.1
## Pelomonas         0.2  1.3  2.8   4.0   0.5
## Stenotrophomonas  0.2  0.1  4.4   3.9   0.0
## Paracoccus        7.0  0.2  0.3   0.0   0.0
## Luteimonas        0.0  0.0  0.6   5.9   0.1
## Undibacterium     0.0  2.1  2.3   0.0   2.1
## Chryseobacterium  1.7  0.5  0.6   2.7   0.0
```

## Figure 6 - Fish Microbiome Samples

Repeat previous steps for fish gut microbiome samples.

```
# Genus nested in phylum

top_nested.f <- nested_top_taxa(ps_rare,
                              top_tax_level = "Phylum",
                              nested_tax_level = "Genus",
                              n_top_taxa = 5, 
                              n_nested_taxa = 4)


# plot 
f.ra <- plot_nested_bar_adj(top_nested.f$ps_obj,
                top_level = "Phylum",
                nested_level = "Genus",
                nested_merged_label = "NA and other <tax>",
                legend_title = "Top phyla and genera") +
  facet_grid(Facility ~ .,
             scales = "free", space = "free_y") +
  theme(legend.key.size = unit(10, "points"),
        legend.position = "right",
        axis.text.y=element_blank(),
        axis.ticks.y=element_blank()) +
  guides(fill = guide_legend(ncol = 1)) +
  theme(panel.spacing.x = unit(0, "pt"),
        panel.border = element_rect(fill = NA, color = "grey2", linetype = "solid",  size = 1)) +
  xlab("Relative Abundance")

f.ra
```

```
#save 
#ggsave("Figures/25_AM/Fig6_RA_Fish.png", f.ra, height = 225, width = 170, units = ("mm"))
```

## Fish and Water Covariation

##### Identify most abundant taxa for fish and water

```
ps_rare_genus <- tax_glom(ps_rare, taxrank = "Genus")

# Convert to relative abundance
ps_rare_genus_rel <- transform_sample_counts(ps_rare_genus, function(x) x/sum(x))

# Aggregate to tank level
tank_rel_genus <- aggregate(. ~ TankID,
                             data = cbind(TankID = meta_rare$TankID,
                             as.data.frame(otu_table(ps_rare_genus_rel))),
                             FUN = mean)

# Set TankID as rownames
rownames(tank_rel_genus) <- tank_rel_genus$TankID
tank_rel_genus$TankID <- NULL

# Add facility metadata
tank_rel_genus_meta <- tank_rel_genus
tank_rel_genus_meta$TankID <- rownames(tank_rel_genus)
tank_rel_genus_meta <- tank_rel_genus_meta %>%
  left_join(meta_tank %>% select(TankID, Facility), by = "TankID")

# Average to facility level
facility_rel_genus <- tank_rel_genus_meta %>%
  group_by(Facility) %>%
  summarise(across(where(is.numeric), mean, na.rm = TRUE)) %>%
  column_to_rownames("Facility")

# Replace ASV IDs with genus names
tax_genus <- as.data.frame(tax_table(ps_rare_genus)) %>%
  rownames_to_column("ASV") %>%
  select(ASV, Genus)

# Rename columns from ASV IDs to genus names
colnames(facility_rel_genus) <- tax_genus$Genus[match(colnames(facility_rel_genus), tax_genus$ASV)]

# Find taxa exceeding 1% mean abundance across all facilities
overall_mean_genus <- colMeans(facility_rel_genus)
top_taxa_genus <- sort(overall_mean_genus[overall_mean_genus >= 0.01], 
                       decreasing = TRUE)
length(top_taxa_genus)
```

```
## [1] 13
```

```
top_taxa_genus
```

```
##    Cetobacterium        Aeromonas           Vibrio      Pseudomonas 
##       0.26265745       0.20748005       0.14030452       0.05619705 
##      Plesiomonas    Streptococcus     Enterococcus      Lactococcus 
##       0.03803552       0.03710009       0.02580493       0.01831354 
##         Bacillus          Delftia Stenotrophomonas   Staphylococcus 
##       0.01526396       0.01303458       0.01116665       0.01064942 
##       Shewanella 
##       0.01014941
```

```
# Facility specific percentages for top taxa
facility_rel_genus %>%
  select(names(top_taxa_genus)) %>%
  t() %>%
  as.data.frame() %>%
  mutate(across(everything(), ~ round(. * 100, 1))) %>%
  arrange(desc(rowMeans(.)))
```

```
##                  Ore1 Ore2 Nor1 Nor2A Nor2B
## Cetobacterium    55.6 48.6  5.6  14.0   7.6
## Aeromonas         3.7  1.2 22.5  15.9  60.4
## Vibrio            5.6 13.2  0.8  44.5   6.0
## Pseudomonas       0.3  0.0 18.6   9.0   0.2
## Plesiomonas       6.6  4.1  2.7   1.9   3.7
## Streptococcus     1.4 12.5  4.7   0.0   0.0
## Enterococcus      0.6 11.4  0.9   0.0   0.0
## Lactococcus       2.1  0.2  6.9   0.0   0.0
## Bacillus          0.3  0.0  0.3   1.9   5.0
## Delftia           0.0  0.0  3.2   3.3   0.0
## Stenotrophomonas  0.0  0.0  3.5   2.0   0.0
## Staphylococcus    0.6  0.0  4.6   0.0   0.0
## Shewanella        1.5  1.0  1.2   0.9   0.5
```

```
# Get all genera present in any fish sample (at any abundance)
fish_genera_names <- colnames(facility_rel_genus)

# Get all genera present in any water sample (at any abundance)
water_genera_names <- colnames(facility_rel_genus_water)

# Top genera from each sample type (>1% mean abundance)
top_fish <- names(top_taxa_genus)
top_water <- names(top_taxa_genus_water)

# Check 1: Are any top water genera absent from fish entirely?
water_unique <- top_water[!top_water %in% fish_genera_names]
cat("Top water genera not found in any fish sample:\n")
```

```
## Top water genera not found in any fish sample:
```

```
print(water_unique)
```

```
## character(0)
```

```
# Check 2: Are any top fish genera absent from water entirely?
fish_unique <- top_fish[!top_fish %in% water_genera_names]
cat("\nTop fish genera not found in any water sample:\n")
```

```
## 
## Top fish genera not found in any water sample:
```

```
print(fish_unique)
```

```
## character(0)
```

```
fish_low_in_water <- top_fish[!top_fish %in% top_water]
cat("Top fish genera not reaching 1% in water:\n")
```

```
## Top fish genera not reaching 1% in water:
```

```
print(fish_low_in_water)
```

```
## [1] "Streptococcus"  "Enterococcus"   "Lactococcus"    "Bacillus"      
## [5] "Staphylococcus"
```

```
# Check which top water genera fall below 1% in fish
water_low_in_fish <- top_water[!top_water %in% top_fish]
cat("\nTop water genera not reaching 1% in fish:\n")
```

```
## 
## Top water genera not reaching 1% in fish:
```

```
print(water_low_in_fish)
```

```
##  [1] "Rheinheimera"     "Acinetobacter"    "Psychrobacter"    "Acidovorax"      
##  [5] "Perlucidibaca"    "Flavobacterium"   "Nevskia"          "Limnobacter"     
##  [9] "Fluviicola"       "Pelomonas"        "Paracoccus"       "Luteimonas"      
## [13] "Undibacterium"    "Chryseobacterium"
```

#### Identify shared genera between fish and water

```
# Get all genera present in fish at any abundance
# using facility_rel_genus which is already tank-averaged
fish_genera_all <- colnames(facility_rel_genus)[colSums(facility_rel_genus) > 0]

# Get all genera present in water at any abundance
water_genera_all <- colnames(facility_rel_genus_water)[colSums(facility_rel_genus_water) > 0]

# Shared genera
shared_genera <- intersect(fish_genera_all, water_genera_all)
cat("Total shared genera:", length(shared_genera), "\n")
```

```
## Total shared genera: 178
```

```
cat("Total unique fish genera:", length(fish_genera_all), "\n")
```

```
## Total unique fish genera: 325
```

```
cat("Total unique water genera:", length(water_genera_all), "\n")
```

```
## Total unique water genera: 324
```

```
cat("Total unique genera overall:", length(union(fish_genera_all, water_genera_all)), "\n")
```

```
## Total unique genera overall: 471
```

```
# Phylum breakdown of shared genera
# Need taxonomy table
tax_shared <- as.data.frame(tax_table(ps_rare_genus)) %>%
  filter(Genus %in% shared_genera) %>%
  group_by(Phylum) %>%
  summarise(n = n()) %>%
  arrange(desc(n))

print(tax_shared)
```

```
## # A tibble: 16 × 2
##    Phylum                n
##    <chr>             <int>
##  1 Proteobacteria       78
##  2 Actinobacteriota     29
##  3 Firmicutes           19
##  4 Bacteroidota         14
##  5 Planctomycetota      14
##  6 Myxococcota           6
##  7 Verrucomicrobiota     6
##  8 Bdellovibrionota      2
##  9 Chloroflexi           2
## 10 Cyanobacteria         2
## 11 Acidobacteriota       1
## 12 Crenarchaeota         1
## 13 Deinococcota          1
## 14 Fusobacteriota        1
## 15 Nitrospirota          1
## 16 Spirochaetota         1
```

```
# Genera abundant in both (>1% in both fish and water)
abundant_both <- shared_genera[
  shared_genera %in% names(top_taxa_genus) & 
  shared_genera %in% names(top_taxa_genus_water)]

cat("\nGenera exceeding 1% in both fish and water:\n")
```

```
## 
## Genera exceeding 1% in both fish and water:
```

```
print(abundant_both)
```

```
## [1] "Shewanella"       "Vibrio"           "Plesiomonas"      "Aeromonas"       
## [5] "Pseudomonas"      "Stenotrophomonas" "Cetobacterium"    "Delftia"
```

```
# Get their abundances in both sample types
data.frame(
  Genus = abundant_both,
  Fish_mean = round(colMeans(facility_rel_genus)[abundant_both] * 100, 1),
  Water_mean = round(colMeans(facility_rel_genus_water)[abundant_both] * 100, 1)
) %>% arrange(desc(Fish_mean))
```

```
##                             Genus Fish_mean Water_mean
## Cetobacterium       Cetobacterium      26.3       13.6
## Aeromonas               Aeromonas      20.7        4.2
## Vibrio                     Vibrio      14.0        4.0
## Pseudomonas           Pseudomonas       5.6        9.0
## Plesiomonas           Plesiomonas       3.8        1.8
## Delftia                   Delftia       1.3        2.5
## Stenotrophomonas Stenotrophomonas       1.1        1.7
## Shewanella             Shewanella       1.0        2.1
```

# SIMPER

## Supplementary Table S3 - Water Microbiome Samples

For water samples, agglomerate taxa by Genus. Create data frames of
taxonomy, OTU, and metadata tables.

```
 ps_rare_genus.w <-
  ps_rare.w%>%
  tax_glom("Genus")
taxonomy = as.data.frame(tax_table(ps_rare_genus.w))
taxonomy$Species = row.names(taxonomy)
rownames(taxonomy) = NULL
abund = as.data.frame(otu_table(ps_rare_genus.w))
meta.abund = as.data.frame(sample_data(ps_rare_genus.w))
fac = meta.abund$Facility
```

Conduct the SIMPER analysis. Select the top 10 significant SIMPER
results per facility-to-facility comparison, adjust p-value for multiple
comparisons, filter by significance, and move to a new dataframe. Use
this dataframe to create a tidy table for publication.

```
set.seed(123)
simp <- simper(abund, fac, permutations = 9999)

comparisons <- c("Ore1_Ore2", "Ore1_Nor2A", "Ore1_Nor2B", "Ore1_Nor1",
                 "Ore2_Nor2A", "Ore2_Nor2B", "Ore2_Nor1",
                 "Nor2A_Nor2B", "Nor2A_Nor1", "Nor2B_Nor1")

simper.results <- c()


for(i in 1:length(comparisons)) {
require(tidyverse)
temp <- summary(simp)[as.character(comparisons[i])] %>%
as.data.frame()
colnames(temp) <- gsub(
paste(comparisons[i],".", sep = ""), "", colnames(temp))
temp <- temp %>%
mutate(Comparison = comparisons[i],
Position = row_number()) %>%
  rownames_to_column(var = "Species")
simper.results <- rbind(simper.results, temp)
}

simper.results <- simper.results %>%
  left_join(taxonomy, by = "Species") %>%
  mutate(Genus = ifelse(is.na(Genus), Species, Genus)) # fallback if no annotation

# Perform BH p-value adjustment
simper.results <- simper.results %>%
  group_by(Comparison) %>%
  mutate(p_adj = p.adjust(p, method = "BH")) %>%
  ungroup()

# Create supplementary table with top 10 significant results per comparison
# Filter for significant results and get top 10 per comparison
suppl_table <- simper.results %>%
  filter(p_adj <= 0.05) %>%
  group_by(Comparison) %>%
  arrange(Position) %>%  # Sort by rank (Position) instead of p-value
  slice_head(n = 10) %>%  # Take top 10 per comparison
  ungroup() %>%
  mutate(
    # Clean up the comparison names for better readability
    Comparison_Clean = case_when(
      Comparison == "Ore1_Ore2"   ~ "Ore1 vs Ore2",
  Comparison == "Ore1_Nor1"   ~ "Ore1 vs Nor1",
  Comparison == "Ore1_Nor2A"  ~ "Ore1 vs Nor2A",
  Comparison == "Ore1_Nor2B"  ~ "Ore1 vs Nor2B",
  Comparison == "Ore2_Nor1"   ~ "Ore2 vs Nor1",
  Comparison == "Ore2_Nor2A"  ~ "Ore2 vs Nor2A",
  Comparison == "Ore2_Nor2B"  ~ "Ore2 vs Nor2B",
  Comparison == "Nor2A_Nor1"  ~ "Nor2A vs Nor1",
  Comparison == "Nor2B_Nor1"  ~ "Nor2B vs Nor1",
  Comparison == "Nor2A_Nor2B" ~ "Nor2A vs Nor2B",
  TRUE ~ Comparison
),
    # Round numerical values for readability
    Average_Contribution = round(average * 100, 2),  # Convert to percentage
    P_adj_value = round(p_adj, 4),
    # Create factor to control order of facility comparisons
    Comparison_Order = factor(Comparison_Clean, levels = c(
      "Ore1 vs Ore2", "Ore1 vs Nor1", "Ore1 vs Nor2A", "Ore1 vs Nor2B", 
      "Ore2 vs Nor1", "Ore2 vs Nor2A", "Ore2 vs Nor2B", 
      "Nor2A vs Nor1", "Nor2B vs Nor1", "Nor2A vs Nor2B"
    ))
  ) %>%
  arrange(Comparison_Order, Position) %>%  # Order by comparison order, then by rank
  select(Comparison_Clean, Genus, Average_Contribution, P_adj_value, Position) %>%
  rename(
    "Facility Comparison" = Comparison_Clean,
    "Genus" = Genus,
    "Average Contribution (%)" = Average_Contribution,
    "p-adj-value" = P_adj_value,
    "Rank" = Position
  )

# Create a formatted table with row groupings per comparison
formatted_table_water <- suppl_table %>%
  kable(format = "html", 
        caption = "Supplementary Table S3: Top 10 most significant ASVs contributing tank water microbiome dissimilarity between facility comparisons (SIMPER analysis, p-adj. < 0.05)",
        align = c("l", "l", "c", "c", "c")) %>%
  kable_styling(bootstrap_options = c("striped", "hover", "condensed"),
                full_width = FALSE,
                font_size = 12) %>%
  column_spec(1, bold = TRUE, width = "3cm") %>%
  column_spec(2, width = "2cm") %>%
  column_spec(3:5, width = "1.5cm")

# Add row groups for each facility comparison
for (comp in unique(suppl_table$`Facility Comparison`)) {
  comp_rows <- which(suppl_table$`Facility Comparison` == comp)
  formatted_table_water <- formatted_table_water %>%
    group_rows(comp, min(comp_rows), max(comp_rows))
}
```

Display formatted table.

```
formatted_table_water
```

Supplementary Table S3: Top 10 most significant ASVs contributing tank
water microbiome dissimilarity between facility comparisons (SIMPER
analysis, p-adj. < 0.05)

| Facility Comparison | Genus | Average Contribution (%) | p-adj-value | Rank |
| --- | --- | --- | --- | --- |
| **Ore1 vs Ore2** | | | | |
| Ore1 vs Ore2 | Cetobacterium | 14.20 | 0.0402 | 1 |
| Ore1 vs Ore2 | Vibrio | 4.58 | 0.0027 | 4 |
| Ore1 vs Ore2 | Plesiomonas | 2.38 | 0.0027 | 8 |
| Ore1 vs Ore2 | Chitinibacter | 1.37 | 0.0027 | 10 |
| Ore1 vs Ore2 | Chitinimonas | 1.01 | 0.0027 | 12 |
| Ore1 vs Ore2 | Crenobacter | 0.63 | 0.0027 | 15 |
| Ore1 vs Ore2 | Haliangium | 0.49 | 0.0027 | 19 |
| Ore1 vs Ore2 | Epulopiscium | 0.45 | 0.0027 | 21 |
| Ore1 vs Ore2 | Rhizorhapis | 0.36 | 0.0491 | 24 |
| Ore1 vs Ore2 | Romboutsia | 0.33 | 0.0027 | 28 |
| **Ore1 vs Nor1** | | | | |
| Ore1 vs Nor1 | Pseudomonas | 17.72 | 0.0040 | 1 |
| Ore1 vs Nor1 | Massilia | 1.84 | 0.0040 | 12 |
| Ore1 vs Nor1 | Variovorax | 1.00 | 0.0040 | 16 |
| Ore1 vs Nor1 | Deinococcus | 0.58 | 0.0208 | 22 |
| Ore1 vs Nor1 | Ottowia | 0.58 | 0.0208 | 23 |
| Ore1 vs Nor1 | Alkanibacter | 0.48 | 0.0040 | 26 |
| Ore1 vs Nor1 | Sphingobacterium | 0.46 | 0.0040 | 27 |
| Ore1 vs Nor1 | Comamonas | 0.28 | 0.0040 | 37 |
| Ore1 vs Nor1 | Bordetella | 0.25 | 0.0360 | 38 |
| Ore1 vs Nor1 | Mesorhizobium | 0.14 | 0.0040 | 52 |
| **Ore1 vs Nor2A** | | | | |
| Ore1 vs Nor2A | Acidovorax | 5.69 | 0.0065 | 4 |
| Ore1 vs Nor2A | Limnobacter | 4.30 | 0.0252 | 6 |
| Ore1 vs Nor2A | Luteimonas | 3.01 | 0.0065 | 11 |
| Ore1 vs Nor2A | Brevundimonas | 2.17 | 0.0065 | 15 |
| Ore1 vs Nor2A | Pelomonas | 2.02 | 0.0295 | 17 |
| Ore1 vs Nor2A | Rhodoferax | 1.06 | 0.0065 | 22 |
| Ore1 vs Nor2A | Pseudoxanthomonas | 0.40 | 0.0108 | 26 |
| Ore1 vs Nor2A | Sphingopyxis | 0.30 | 0.0292 | 29 |
| Ore1 vs Nor2A | Caulobacter | 0.28 | 0.0065 | 33 |
| Ore1 vs Nor2A | Dyadobacter | 0.27 | 0.0324 | 34 |
| **Ore1 vs Nor2B** | | | | |
| Ore1 vs Nor2B | Rheinheimera | 16.98 | 0.0054 | 1 |
| Ore1 vs Nor2B | Fluviicola | 5.54 | 0.0054 | 5 |
| Ore1 vs Nor2B | Polynucleobacter | 1.48 | 0.0324 | 12 |
| Ore1 vs Nor2B | Sediminibacterium | 1.31 | 0.0054 | 13 |
| Ore1 vs Nor2B | hgcI clade | 1.24 | 0.0243 | 14 |
| Ore1 vs Nor2B | Candidatus Nitrosotenuis | 1.10 | 0.0054 | 15 |
| Ore1 vs Nor2B | Candidatus Omnitrophus | 0.34 | 0.0324 | 28 |
| Ore1 vs Nor2B | Cellvibrio | 0.29 | 0.0139 | 33 |
| Ore1 vs Nor2B | Xanthobacter | 0.14 | 0.0054 | 43 |
| Ore1 vs Nor2B | Sulfurifustis | 0.09 | 0.0324 | 55 |
| **Ore2 vs Nor1** | | | | |
| Ore2 vs Nor1 | Cetobacterium | 18.45 | 0.0093 | 1 |
| Ore2 vs Nor1 | Pseudomonas | 18.07 | 0.0036 | 2 |
| Ore2 vs Nor1 | Vibrio | 4.23 | 0.0481 | 3 |
| Ore2 vs Nor1 | Plesiomonas | 2.62 | 0.0133 | 6 |
| Ore2 vs Nor1 | Massilia | 1.87 | 0.0036 | 9 |
| Ore2 vs Nor1 | Variovorax | 1.03 | 0.0036 | 15 |
| Ore2 vs Nor1 | Nubsella | 0.95 | 0.0397 | 17 |
| Ore2 vs Nor1 | Crenobacter | 0.64 | 0.0169 | 20 |
| Ore2 vs Nor1 | Deinococcus | 0.60 | 0.0136 | 21 |
| Ore2 vs Nor1 | Ottowia | 0.60 | 0.0169 | 22 |
| **Ore2 vs Nor2A** | | | | |
| Ore2 vs Nor2A | Cetobacterium | 20.87 | 0.0065 | 1 |
| Ore2 vs Nor2A | Acidovorax | 5.70 | 0.0046 | 2 |
| Ore2 vs Nor2A | Nevskia | 5.28 | 0.0438 | 3 |
| Ore2 vs Nor2A | Limnobacter | 4.57 | 0.0046 | 4 |
| Ore2 vs Nor2A | Plesiomonas | 3.12 | 0.0046 | 9 |
| Ore2 vs Nor2A | Luteimonas | 3.06 | 0.0046 | 10 |
| Ore2 vs Nor2A | Brevundimonas | 2.21 | 0.0046 | 13 |
| Ore2 vs Nor2A | Methyloversatilis | 1.38 | 0.0477 | 17 |
| Ore2 vs Nor2A | Rhodoferax | 1.07 | 0.0046 | 21 |
| Ore2 vs Nor2A | Crenobacter | 0.67 | 0.0216 | 25 |
| **Ore2 vs Nor2B** | | | | |
| Ore2 vs Nor2B | Rheinheimera | 17.18 | 0.0054 | 2 |
| Ore2 vs Nor2B | Fluviicola | 5.53 | 0.0054 | 4 |
| Ore2 vs Nor2B | Polynucleobacter | 1.49 | 0.0383 | 11 |
| Ore2 vs Nor2B | Sediminibacterium | 1.33 | 0.0054 | 14 |
| Ore2 vs Nor2B | hgcI clade | 1.25 | 0.0227 | 15 |
| Ore2 vs Nor2B | Candidatus Nitrosotenuis | 1.11 | 0.0054 | 17 |
| Ore2 vs Nor2B | Candidatus Omnitrophus | 0.35 | 0.0180 | 28 |
| Ore2 vs Nor2B | Cellvibrio | 0.28 | 0.0180 | 30 |
| Ore2 vs Nor2B | Xanthobacter | 0.14 | 0.0054 | 42 |
| Ore2 vs Nor2B | Sulfurifustis | 0.09 | 0.0180 | 50 |
| **Nor2A vs Nor1** | | | | |
| Nor2A vs Nor1 | Pseudomonas | 14.86 | 0.0054 | 1 |
| Nor2A vs Nor1 | Acidovorax | 5.43 | 0.0054 | 2 |
| Nor2A vs Nor1 | Delftia | 5.09 | 0.0208 | 4 |
| Nor2A vs Nor1 | Limnobacter | 4.34 | 0.0162 | 5 |
| Nor2A vs Nor1 | Luteimonas | 2.73 | 0.0054 | 8 |
| Nor2A vs Nor1 | Brevundimonas | 2.03 | 0.0054 | 11 |
| Nor2A vs Nor1 | Massilia | 1.81 | 0.0054 | 13 |
| Nor2A vs Nor1 | Methyloversatilis | 1.45 | 0.0324 | 16 |
| Nor2A vs Nor1 | Rhodoferax | 1.03 | 0.0162 | 20 |
| Nor2A vs Nor1 | Variovorax | 1.01 | 0.0054 | 21 |
| **Nor2B vs Nor1** | | | | |
| Nor2B vs Nor1 | Pseudomonas | 17.23 | 0.0463 | 1 |
| Nor2B vs Nor1 | Rheinheimera | 16.43 | 0.0162 | 2 |
| Nor2B vs Nor1 | Fluviicola | 5.36 | 0.0162 | 4 |
| Nor2B vs Nor1 | Sediminibacterium | 1.20 | 0.0463 | 14 |
| Nor2B vs Nor1 | Candidatus Omnitrophus | 0.33 | 0.0468 | 34 |
| Nor2B vs Nor1 | Cellvibrio | 0.28 | 0.0454 | 38 |
| Nor2B vs Nor1 | Xanthobacter | 0.14 | 0.0162 | 53 |
| Nor2B vs Nor1 | Sulfurifustis | 0.09 | 0.0468 | 67 |
| Nor2B vs Nor1 | HdN1 | 0.06 | 0.0162 | 83 |
| **Nor2A vs Nor2B** | | | | |
| Nor2A vs Nor2B | Rheinheimera | 13.35 | 0.0243 | 1 |
| Nor2A vs Nor2B | Fluviicola | 5.44 | 0.0108 | 3 |
| Nor2A vs Nor2B | Candidatus Nitrosotenuis | 1.08 | 0.0324 | 21 |
| Nor2A vs Nor2B | Candidatus Omnitrophus | 0.34 | 0.0446 | 30 |
| Nor2A vs Nor2B | Cellvibrio | 0.29 | 0.0259 | 33 |
| Nor2A vs Nor2B | Xanthobacter | 0.14 | 0.0108 | 43 |
| Nor2A vs Nor2B | Sulfurifustis | 0.09 | 0.0446 | 50 |
| Nor2A vs Nor2B | HdN1 | 0.06 | 0.0108 | 53 |

```
#write.csv(suppl_table, "Figures/25_AM/S5WaterSIMPER.csv", row.names = TRUE)
```

## Supplementary Table S5 - Fish Microbiome Samples

Repeat previous steps for fish microbiome samples.

```
 ps_tank_genus <-
  ps_tank%>%
  tax_glom("Genus")
taxonomy_fish = as.data.frame(tax_table(ps_tank_genus))
taxonomy_fish$Species = row.names(taxonomy_fish)
rownames(taxonomy_fish) = NULL
abund_fish = as.data.frame(otu_table(ps_tank_genus))
meta.abund.fish = as.data.frame(sample_data(ps_tank_genus))
fac_fish = meta.abund.fish$Facility
```

```
# calculate SIMPER
set.seed(123)
simp.fish <- simper(abund_fish, fac_fish, permutations = 9999)

# define comparison groups
simper.results.fish <- c()

# create new table for grouped SIMPER values
for(i in 1:length(comparisons)) {
require(tidyverse)
temp1 <- summary(simp.fish)[as.character(comparisons[i])] %>%
as.data.frame()
colnames(temp1) <- gsub(
paste(comparisons[i],".", sep = ""), "", colnames(temp1))
temp1 <- temp1 %>%
mutate(Comparison = comparisons[i],
Position = row_number()) %>%
  rownames_to_column(var = "Species")
simper.results.fish <- rbind(simper.results.fish, temp1)
}

simper.results.fish <- simper.results.fish %>%
  left_join(taxonomy_fish, by = "Species") %>%
  mutate(Genus = ifelse(is.na(Genus), Species, Genus)) # fallback if no annotation

# Perform BH p-value adjustment
simper.results.fish <- simper.results.fish %>%
  group_by(Comparison) %>%
  mutate(p_adj = p.adjust(p, method = "BH")) %>%
  ungroup()

# Create supplementary table with top 10 significant results per comparison
# Filter for significant results and get top 10 per comparison
suppl_table1 <- simper.results.fish %>%
  filter(p_adj <= 0.05) %>%
  group_by(Comparison) %>%
  arrange(Position) %>%  # Sort by rank (Position) instead of p-value
  slice_head(n = 10) %>%  # Take top 10 per comparison
  ungroup() %>%
  mutate(
    # Clean up the comparison names for better readability
    Comparison_Clean = case_when(
      Comparison == "Ore1_Ore2" ~ "Ore1 vs Ore2",
      Comparison == "Ore1_Nor1" ~ "Ore1 vs Nor1", 
      Comparison == "Ore1_Nor2A" ~ "Ore1 vs Nor2A",
      Comparison == "Ore1_Nor2B" ~ "Ore1 vs Nor2B",
      Comparison == "Nor1_Ore2" ~ "Ore2 vs Nor1",
      Comparison == "Nor2A_Ore2" ~ "Ore2 vs Nor2A", 
      Comparison == "Nor2B_Ore2" ~ "Ore2 vs Nor2B",
      Comparison == "Nor2A_Nor1" ~ "Nor2A vs Nor1",
      Comparison == "Nor2B_Nor1" ~ "Nor2B vs Nor1",
      Comparison == "Nor2B_Nor2A" ~ "Nor2A vs Nor2B",
      TRUE ~ Comparison
    ),
    # Round numerical values for readability
    Average_Contribution = round(average * 100, 2),  # Convert to percentage
    P_adj_value = round(p_adj, 4),
    # Create factor to control order of facility comparisons
    Comparison_Order = factor(Comparison_Clean, levels = c(
      "Ore1 vs Ore2", "Ore1 vs Nor1", "Ore1 vs Nor2A", "Ore1 vs Nor2B", 
      "Ore2 vs Nor1", "Ore2 vs Nor2A", "Ore2 vs Nor2B", 
      "Nor2A vs Nor1", "Nor2B vs Nor1", "Nor2A vs Nor2B"
    ))
  ) %>%
  arrange(Comparison_Order, Position) %>%  # Order by comparison order, then by rank
  select(Comparison_Clean, Genus, Average_Contribution, P_adj_value, Position) %>%
  rename(
    "Facility Comparison" = Comparison_Clean,
    "Genus" = Genus,
    "Average Contribution (%)" = Average_Contribution,
    "p-adj-value" = P_adj_value,
    "Rank" = Position
  )

# Create a formatted table with row groupings per comparison
formatted_table_fish <- suppl_table1 %>%
  kable(format = "html", 
        caption = "Supplementary Table S5: Top 10 most significant ASVs contributing to fish gut microbiome dissimilarity between facility comparisons (SIMPER analysis, p < 0.05)",
        align = c("l", "l", "c", "c", "c")) %>%
  kable_styling(bootstrap_options = c("striped", "hover", "condensed"),
                full_width = FALSE,
                font_size = 12) %>%
  column_spec(1, bold = TRUE, width = "3cm") %>%
  column_spec(2, width = "2cm") %>%
  column_spec(3:5, width = "1.5cm")

# Add row groups for each facility comparison
for (comp in unique(suppl_table1$`Facility Comparison`)) {
  comp_rows <- which(suppl_table1$`Facility Comparison` == comp)
  formatted_table_fish <- formatted_table_fish %>%
    group_rows(comp, min(comp_rows), max(comp_rows))
}
```

Display formatted fish microbiome table.

```
formatted_table_fish
```

Supplementary Table S5: Top 10 most significant ASVs contributing to
fish gut microbiome dissimilarity between facility comparisons (SIMPER
analysis, p < 0.05)

| Facility Comparison | Genus | Average Contribution (%) | p-adj-value | Rank |
| --- | --- | --- | --- | --- |
| **Ore1 vs Nor1** | | | | |
| Ore1 vs Nor1 | Cetobacterium | 25.09 | 0.0046 | 1 |
| Ore1 vs Nor1 | Pseudomonas | 9.50 | 0.0098 | 3 |
| Ore1 vs Nor1 | Lactococcus | 3.11 | 0.0175 | 5 |
| Ore1 vs Nor1 | Achromobacter | 2.45 | 0.0046 | 8 |
| Ore1 vs Nor1 | Pediococcus | 2.20 | 0.0046 | 9 |
| Ore1 vs Nor1 | Leuconostoc | 1.60 | 0.0046 | 13 |
| Ore1 vs Nor1 | Klebsiella | 0.33 | 0.0148 | 26 |
| Ore1 vs Nor1 | Limosilactobacillus | 0.26 | 0.0046 | 30 |
| Ore1 vs Nor1 | IMCC26207 | 0.21 | 0.0046 | 35 |
| Ore1 vs Nor1 | Nocardioides | 0.07 | 0.0255 | 59 |
| **Ore1 vs Nor2A** | | | | |
| Ore1 vs Nor2A | Vibrio | 20.92 | 0.0325 | 1 |
| **Ore1 vs Nor2B** | | | | |
| Ore1 vs Nor2B | Aeromonas | 27.54 | 0.0022 | 1 |
| Ore1 vs Nor2B | Cetobacterium | 25.70 | 0.0050 | 2 |
| Ore1 vs Nor2B | Crenobacter | 0.63 | 0.0022 | 15 |
| Ore1 vs Nor2B | Chelativorans | 0.57 | 0.0022 | 17 |
| Ore1 vs Nor2B | Marmoricola | 0.34 | 0.0022 | 21 |
| Ore1 vs Nor2B | Chitinilyticum | 0.21 | 0.0022 | 29 |
| Ore1 vs Nor2B | Flavobacterium | 0.19 | 0.0022 | 30 |
| Ore1 vs Nor2B | Nannocystis | 0.18 | 0.0022 | 33 |
| Ore1 vs Nor2B | Pirellula | 0.15 | 0.0134 | 41 |
| Ore1 vs Nor2B | Fictibacillus | 0.12 | 0.0028 | 47 |
| **Nor2A vs Nor1** | | | | |
| Nor2A vs Nor1 | Vibrio | 22.13 | 0.0108 | 1 |
| Nor2A vs Nor1 | Lactococcus | 3.48 | 0.0108 | 5 |
| Nor2A vs Nor1 | Pediococcus | 2.14 | 0.0163 | 11 |
| Nor2A vs Nor1 | Leuconostoc | 1.57 | 0.0108 | 13 |
| Nor2A vs Nor1 | Limosilactobacillus | 0.26 | 0.0260 | 26 |
| Nor2A vs Nor1 | Levilactobacillus | 0.06 | 0.0487 | 43 |
| **Nor2B vs Nor1** | | | | |
| Nor2B vs Nor1 | Crenobacter | 0.59 | 0.0163 | 18 |
| Nor2B vs Nor1 | Chelativorans | 0.56 | 0.0292 | 19 |
| Nor2B vs Nor1 | Marmoricola | 0.32 | 0.0292 | 26 |
| Nor2B vs Nor1 | Chitinilyticum | 0.20 | 0.0163 | 33 |
| Nor2B vs Nor1 | Flavobacterium | 0.19 | 0.0292 | 34 |
| Nor2B vs Nor1 | Nannocystis | 0.18 | 0.0292 | 35 |
| Nor2B vs Nor1 | Pirellula | 0.14 | 0.0352 | 37 |
| Nor2B vs Nor1 | Leptolyngbya ANT.L52.2 | 0.09 | 0.0292 | 48 |
| Nor2B vs Nor1 | OM60(NOR5) clade | 0.06 | 0.0292 | 62 |
| Nor2B vs Nor1 | Ellin6067 | 0.05 | 0.0292 | 68 |

```
write.csv(suppl_table1, "Figures/25_AM/S5FishSIMPER.csv", row.names = TRUE)
```

# Figure 9 - FEAST Source-Tracking

In the first step of the FEAST analysis, data are analyzed using the
facility ‘true’ names (see Figure 1 in the manuscript; e.g., Huestis,
ZIRC, JutfeltA). In the second step, these are corrected to the
standardized facility identifiers (e.g., Ore1, Ore2). All FEAST output
files are saved locally.

Create FEAST output files where, for each facility, all fish samples
within that facility are identified as sinks and tank water samples as
potential sources. Repeat this with fish samples identified as potential
sources and tank water samples as sinks.

```
# create metadata table and OTU table for FEAST and pipe through

facilities = as.character(unique(data.frame(sample_data(ps_rare.all))$FacilityB))
# facilities = facilities[1]
waterorfish = c('water', 'fish')

# waterorfish = 'water'
for (fac in facilities) {
  for (sink in waterorfish) {
    if (sink == 'water') source = 'fish'
    else if (sink == 'fish') source = 'water'
    data.frame(sample_data(ps_rare.all)) %>%
      as_tibble(rownames = "SampleID") %>%
      select(SampleID, Env = FacType) %>%
      mutate(SourceSink = case_when(grepl(sink, Env) ~ "Sink", grepl(source, Env) ~ "Source")) %>%
      arrange(SourceSink) %>%
      filter(grepl(paste0(sink, '_', fac, '|', source, '_', fac), Env)) %>%
      mutate(id = row_number()) %>%
      mutate(id = ifelse(SourceSink == "Source", "", id)) ->
      meta.source

    data.frame(otu_table(ps_rare.all))[meta.source$SampleID, ] ->
      otu.source
    meta.source <- column_to_rownames(meta.source, var = "SampleID") 
    otu.source <- as.matrix(otu.source)

    outfile = paste0(fac, "_", sink)
    FEAST(C = otu.source, metadata = meta.source, different_sources_flag = 0, dir_path = "C:/Users/kayla/Documents/Dissertation/Fish Facility Project/Analyses/FEAST/am2025", outfile=outfile)
  }
}
```

```
# Get fish-to-tank mapping from individual fish phyloseq
fish_tank_map <- data.frame(sample_data(ps_rare)) %>%
  rownames_to_column("SampleID") %>%
  select(SampleID, TankID, Facility)

# Get water-to-tank mapping
water_tank_map <- data.frame(sample_data(ps_rare.w)) %>%
  rownames_to_column("SampleID") %>%
  select(SampleID, TankID, Facility)
```

#### Fish as Sink

```
# Function to process FEAST output
process_feast_output <- function(output_file, fish_meta, water_meta) {
  
  output <- read.delim(output_file)
  
  results <- lapply(rownames(output), function(fish_id) {
    
    # Strip facility suffix to get original Sample_ID
    clean_fish_id <- gsub("_fish_.*", "", fish_id)
    
    # Get this fish's tank and facility
    fish_info <- fish_meta[fish_meta$SampleID == clean_fish_id, ]
    fish_tank <- fish_info$TankID
    fish_fac <- fish_info$Facility
    
    # Get source columns (not Unknown)
    source_cols <- colnames(output)[colnames(output) != "Unknown"]
    
    # Extract water Sample_IDs from column names
    water_ids_in_output <- gsub("_water_.*", "", source_cols)
    
    # Match to water metadata
    water_info <- water_meta[match(water_ids_in_output, water_meta$SampleID), ]
    
    # Categorize columns
    same_tank_cols <- source_cols[!is.na(water_info$TankID) & 
                                   water_info$TankID == fish_tank]
    
    same_fac_cols <- source_cols[!is.na(water_info$Facility) & 
                                  water_info$Facility == fish_fac &
                                  !source_cols %in% same_tank_cols]
    
    # Sum contributions by category
    same_tank_sum <- if(length(same_tank_cols) > 0) {
      sum(output[fish_id, same_tank_cols], na.rm = TRUE)
    } else { NA }
    
    same_fac_sum <- if(length(same_fac_cols) > 0) {
      sum(output[fish_id, same_fac_cols], na.rm = TRUE)
    } else { 0 }
    
    data.frame(
      SampleID = fish_id,
      TankID = fish_tank,
      Facility = fish_fac,
      same_tank = same_tank_sum,
      same_facility = same_fac_sum,
      unknown = output[fish_id, "Unknown"]
    )
  }) %>% bind_rows()
  
  return(results)
}
```

```
# Using existing file names with old facility names
feast_ore1 <- process_feast_output(
  "FEAST/am2025/Huestis_fish_source_contributions_matrix.txt",
  fish_tank_map, water_tank_map)

feast_ore2 <- process_feast_output(
  "FEAST/am2025/ZIRC_fish_source_contributions_matrix.txt",
  fish_tank_map, water_tank_map)

feast_nor1 <- process_feast_output(
  "FEAST/am2025/Yaksi_fish_source_contributions_matrix.txt",
  fish_tank_map, water_tank_map)

feast_nor2a <- process_feast_output(
  "FEAST/am2025/JutfeltA_fish_source_contributions_matrix.txt",
  fish_tank_map, water_tank_map)

feast_nor2b <- process_feast_output(
  "FEAST/am2025/JutfeltB_fish_source_contributions_matrix.txt",
  fish_tank_map, water_tank_map)
```

```
# Combine all facilities
feast_all <- bind_rows(feast_ore1, feast_ore2, feast_nor1, 
                        feast_nor2a, feast_nor2b) %>%
  mutate(Facility = factor(Facility, 
                            levels = c("Ore1", "Ore2", "Nor1", "Nor2A", "Nor2B")))

# run the summary
feast_summary <- feast_all %>% 
  group_by(Facility) %>% 
  summarise(
    n = n(),
    mean_same_tank = mean(same_tank, na.rm = TRUE),
    mean_same_facility = mean(same_facility, na.rm = TRUE),
    mean_unknown = mean(unknown, na.rm = TRUE),
    n_no_tank_water = sum(is.na(same_tank))
  )

write.csv(feast_summary, "FEAST/am2025/feast_summary.csv", row.names = FALSE)
```

```
feast_long <- feast_all %>%
  mutate(
    same_tank = pmin(same_tank, 1),
    same_facility = pmin(same_facility, 1),
    unknown = pmin(unknown, 1)
  ) %>%
  pivot_longer(cols = c(same_tank, same_facility, unknown),
               names_to = "Source",
               values_to = "Proportion") %>%
  mutate(
    Source = factor(Source,
                    levels = c("same_tank", "same_facility", "unknown"),
                    labels = c("Same Tank", "Same Facility", "Unknown")),
    Facility = factor(Facility,
                      levels = c("Ore1", "Ore2", "Nor1", "Nor2A", "Nor2B"))
  )

# Plot
feast_plot <- ggplot(feast_long, 
                      aes(x = Source, y = Proportion,
                          fill = Facility, color = Facility)) +
  facet_wrap(~ Facility, nrow = 1) +
  geom_boxplot(alpha = 0.6, outlier.shape = NA, width = 0.6) +
  geom_jitter(width = 0.1, size = 1.2, alpha = 0.7) +
  scale_fill_manual(values = locuszoom) +
  scale_color_manual(values = locuszoom) +
  scale_y_continuous(limits = c(0, 1)) +
  theme_bw() +
  theme(
    panel.grid.major.y = element_line(color = "grey90"),
    panel.grid.major.x = element_blank(),
    panel.grid.minor.y = element_line(color = "grey90"),
    panel.background = element_rect(colour = "black", linewidth = 0.5),
    axis.text.x = element_text(angle = 45, hjust = 1),
    legend.position = "none",
    strip.background = element_blank(),
    strip.text = element_text(face = "bold")
  ) +
  labs(x = "", y = "Source Proportion")
```

#### Fish as Source

```
process_feast_output_water <- function(output_file, fish_meta, water_meta) {
  
  output <- read.delim(output_file)
  
  results <- lapply(rownames(output), function(water_id) {
    
    # Strip facility suffix to get original water Sample_ID
    clean_water_id <- gsub("_water_.*", "", water_id)
    
    # Get this water sample's tank and facility
    water_info <- water_meta[water_meta$SampleID == clean_water_id, ]
    water_tank <- water_info$TankID
    water_fac <- water_info$Facility
    
    # Get source columns (not Unknown)
    source_cols <- colnames(output)[colnames(output) != "Unknown"]
    
    # Extract fish Sample_IDs from column names
    fish_ids_in_output <- gsub("_fish_.*", "", source_cols)
    
    # Match to fish metadata
    fish_info_all <- fish_meta[match(fish_ids_in_output, fish_meta$SampleID), ]
    
    # Categorize columns
    same_tank_cols <- source_cols[!is.na(fish_info_all$TankID) & 
                                   fish_info_all$TankID == water_tank]
    
    same_fac_cols <- source_cols[!is.na(fish_info_all$Facility) & 
                                  fish_info_all$Facility == water_fac &
                                  !source_cols %in% same_tank_cols]
    
    # Sum contributions by category
    same_tank_sum <- if(length(same_tank_cols) > 0) {
      sum(output[water_id, same_tank_cols], na.rm = TRUE)
    } else { NA }
    
    same_fac_sum <- if(length(same_fac_cols) > 0) {
      sum(output[water_id, same_fac_cols], na.rm = TRUE)
    } else { 0 }
    
    data.frame(
      SampleID = water_id,
      TankID = water_tank,
      Facility = water_fac,
      same_tank = same_tank_sum,
      same_facility = same_fac_sum,
      unknown = output[water_id, "Unknown"]
    )
  }) %>% bind_rows()
  
  return(results)
}

# Run for all facilities using old file names
feast_water_ore1 <- process_feast_output_water(
  "FEAST/am2025/Huestis_water_source_contributions_matrix.txt",
  fish_tank_map, water_tank_map)

feast_water_ore2 <- process_feast_output_water(
  "FEAST/am2025/ZIRC_water_source_contributions_matrix.txt",
  fish_tank_map, water_tank_map)

feast_water_nor1 <- process_feast_output_water(
  "FEAST/am2025/Yaksi_water_source_contributions_matrix.txt",
  fish_tank_map, water_tank_map)

feast_water_nor2a <- process_feast_output_water(
  "FEAST/am2025/JutfeltA_water_source_contributions_matrix.txt",
  fish_tank_map, water_tank_map)

feast_water_nor2b <- process_feast_output_water(
  "FEAST/am2025/JutfeltB_water_source_contributions_matrix.txt",
  fish_tank_map, water_tank_map)

# Combine all facilities
feast_water_all <- bind_rows(
  feast_water_ore1, feast_water_ore2, feast_water_nor1,
  feast_water_nor2a, feast_water_nor2b) %>%
  mutate(Facility = factor(Facility,
                            levels = c("Ore1", "Ore2", "Nor1", "Nor2A", "Nor2B")))

# Summary
feast_water_summary <- feast_water_all %>%
  group_by(Facility) %>%
  summarise(
    n = n(),
    mean_same_tank = round(mean(same_tank, na.rm = TRUE), 4),
    mean_same_facility = round(mean(same_facility, na.rm = TRUE), 4),
    mean_unknown = round(mean(unknown, na.rm = TRUE), 4),
    n_no_tank_fish = sum(is.na(same_tank))
  )

print(feast_water_summary)
```

```
## # A tibble: 5 × 6
##   Facility     n mean_same_tank mean_same_facility mean_unknown n_no_tank_fish
##   <ord>    <int>          <dbl>              <dbl>        <dbl>          <int>
## 1 Ore1        22         0.0222             0.484         0.495              1
## 2 Ore2        12         0.503              0.212         0.746             11
## 3 Nor1         7         0.0386             0.199         0.763              0
## 4 Nor2A        6         0.0411             0.231         0.728              0
## 5 Nor2B        3         0.0504             0.0319        0.918              0
```

## Figure 9 Plot

Remove the source proportion sums from each dataframe from each
facility. Combine these into one dataframe and the corresponding
facility identity. Repeat for both fish and water samples.

```
# Reshape water sink data for plotting, excluding Ore2
feast_water_long <- feast_water_all %>%
  filter(Facility != "Ore2") %>%
  mutate(
    same_tank = pmin(same_tank, 1),
    same_facility = pmin(same_facility, 1),
    unknown = pmin(unknown, 1)
  ) %>%
  pivot_longer(cols = c(same_tank, same_facility, unknown),
               names_to = "Source",
               values_to = "Proportion") %>%
  mutate(
    Source = factor(Source,
                    levels = c("same_tank", "same_facility", "unknown"),
                    labels = c("Same Tank", "Same Facility", "Unknown")),
    Facility = factor(Facility,
                      levels = c("Ore1", "Nor1", "Nor2A", "Nor2B"))
  )

# Water as sink plot
feast_plot_water <- ggplot(feast_water_long,
                            aes(x = Source, y = Proportion,
                                fill = Facility, color = Facility)) +
  facet_wrap(~ Facility, nrow = 1) +
  geom_boxplot(alpha = 0.6, outlier.shape = NA, width = 0.6) +
  geom_jitter(width = 0.1, size = 1.2, alpha = 0.7) +
  scale_fill_manual(values = locuszoom[c(1, 3, 4, 5)]) +
  scale_color_manual(values = locuszoom[c(1, 3, 4, 5)]) +
  scale_y_continuous(limits = c(0, 1)) +
  theme_bw() +
  theme(
    panel.grid.major.y = element_line(color = "grey90"),
    panel.grid.major.x = element_blank(),
    panel.grid.minor.y = element_line(color = "grey90"),
    panel.background = element_rect(colour = "black", linewidth = 0.5),
    axis.text.x = element_text(angle = 45, hjust = 1),
    legend.position = "none",
    strip.background = element_blank(),
    strip.text = element_text(face = "bold")
  ) +
  labs(x = "", y = "Source Proportion",
       title = "Water as Sink")

# Also add title to fish plot for clarity
feast_plot_fish <- feast_plot + 
  labs(title = "Fish as Sink")

# Combine with ggarrange
feast_combined <- ggarrange(feast_plot_fish, feast_plot_water,
                              ncol = 1, nrow = 2,
                              labels = "AUTO")

feast_combined
```

```
ggsave("Figures/25_AM/Fig9_FEAST_combined.png", feast_combined,
        width = 170, height = 200, units = "mm", dpi = 300)
```
