# Supplementary material for: Aquaculture facility-specific microbiota shape the zebrafish gut microbiome
Source: Anim Microbiome. 2026 May 7;8:84. doi: 10.1186/s42523-026-00573-6 (PMC13321582; doi:10.1186/s42523-026-00573-6)
Supplement: Supplementary file 5 — Supplementary Material 5: Exclusion-based sensitivity analyses for Ore2 fish gut microbiome samples [file 42523_2026_573_MOESM5_ESM.pdf]

Supplementary Material 5 – Exclusion-based sensitivity analyses for Ore2 fish gut microbiome samples

A. Alpha Diversity

ANOVA

**Shannon index (H)**					
Sum Sq	Mean Sq	NumDF	DenDF	F value	Pr(>F)
11.319	3.773	3	34.353	6.655	0.001
**Inverse Simpson index (1-λ)**					
112.540	37.513	3	25.102	2.109	0.124

Post-hoc pairwise comparisons

**Shannon index (H)**								
Estimated Marginal Means								
Facility	emmean	SE	df	lower.CL	upper.CL	estimate	t.ratio	p.value (adj.)
Nor1	1.71	0.18	27.44	1.35	2.07			
Nor2A	0.84	0.20	39.54	0.43	1.25			
Nor2B	1.96	0.27	29.37	1.42	2.51			
Ore1	1.86	0.13	43.23	1.60	2.12			
Pairwise Contrasts								
Nor1 - Nor2A		0.27	33.66			0.87	3.23	**0.01**
Nor1 - Nor2B		0.32	28.76			-0.25	-0.80	0.59
Nor1 - Ore1		0.22	31.99			-0.15	-0.70	0.59
Nor2A - Nor2B		0.33	32.67			-1.12	-3.35	**0.01**
Nor2A - Ore1		0.24	40.56			-1.02	-4.22	**0.00**
Nor2B - Ore1		0.30	31.52			0.10	0.34	0.73
**Inverse Simpson index (1-λ)**								
Estimated Marginal Means								
Nor1	4.90	0.82	23.75	3.21	6.59			
Nor2A	1.98	0.98	40.60	-0.01	3.96			
Nor2B	3.43	1.24	27.43	0.88	5.98			
Ore1	4.50	0.63	45.51	3.23	5.77			
Pairwise Contrasts								
Nor1 - Nor2A		1.28	32.24			2.92	2.29	0.11
Nor1 - Nor2B		1.49	26.25			1.47	0.98	0.54
Nor1 - Ore1		1.03	29.81			0.39	0.38	0.70
Nor2A - Nor2B		1.58	31.69			-1.46	-0.92	0.54
Nor2A - Ore1		1.17	41.97			-2.53	-2.17	0.11
Nor2B - Ore1		1.40	30.22			-1.07	-0.77	0.54
Nor1 - Nor2A		1.28	32.24			2.92	2.29	0.11
Nor1 - Nor2B		1.49	26.25			1.47	0.98	0.54

B. Beta Diversity

PERMANOVA: Effects of Location, Facility, and Genotype

**Bray-Curtis: Hierarchical**					
	Df	SumOfSqs	R2	F	Pr(>F)
Location	1	3.741	0.325	25.377	< 0.001
Geno_Status	1	0.530	0.046	3.594	0.007
Location:Facility	2	1.785	0.155	6.053	< 0.001
Residual	37	5.455	0.474	NA	NA
Total	41	11.510	1.000	NA	NA
**Bray-Curtis: Constrained**					
Geno_Status	1.000	2.343	0.204	10.224	0.339
Residual	40.000	9.167	0.796	NA	NA
Total	41.000	11.510	1.000	NA	NA
**Unweighted UniFrac: Hierarchical**					
Location	1	2.230	0.209	12.845	< 0.001
Geno_Status	1	0.675	0.063	3.888	< 0.001
Location:Facility	2	1.319	0.124	3.800	< 0.001
Residual	37	6.423	0.603	NA	NA
**Unweighted UniFrac: Constrained**					
Geno_Status	1	1.887	0.177	8.617	0.446
Residual	40	8.760	0.823	NA	NA
Total	41	10.647	1.000	NA	NA

Pairwise PERMANOVA

**Bray-Curtis**						
pairs	Df	SumsOfSqs	F.Model	R2	p.value	p.adjusted
Ore1 vs Nor2B	1	1.945	17.305	0.429	< 0.001	< 0.001
Ore1 vs Nor2A	1	2.094	15.498	0.365	< 0.001	< 0.001
Ore1 vs Nor1	1	3.127	22.394	0.444	< 0.001	< 0.001
Nor2B vs Nor2A	1	0.924	5.546	0.357	0.013	0.013
Nor2B vs Nor1	1	0.922	5.258	0.323	0.001	0.001
Nor2A vs Nor1	1	1.350	6.768	0.311	< 0.001	< 0.001
**Unweighted UniFrac**						
Ore1 vs Nor2B	1	1.079	6.589	0.223	< 0.001	< 0.001
Ore1 vs Nor2A	1	2.167	13.468	0.333	< 0.001	< 0.001
Ore1 vs Nor1	1	1.400	8.092	0.224	< 0.001	< 0.001
Nor2B vs Nor2A	1	0.982	5.589	0.359	0.002	0.003
Nor2B vs Nor1	1	0.732	3.568	0.245	0.005	0.005
Nor2A vs Nor1	1	0.992	5.247	0.259	< 0.001	< 0.001

C. Fish and Water Covariation

PERMANOVA (Bray-Curtis distance)

**Sample Type + Facility**					
Sample_Type	1	2.8645	0.10278	11.8688	< 0.001
Facility	3	6.9039	0.24772	9.5352	< 0.001
Residual	75	18.1011	0.64949	NA	NA
Total	79	27.8696	1.00000	NA	NA
**Sample Type * Facility**					
Sample_Type	1	2.8645	0.10278	15.6178	< 0.001
Facility	3	6.9039	0.24772	12.5471	< 0.001
Sample_Type:Facility	3	4.8953	0.17565	8.8967	< 0.001
Residual	72	13.2058	0.47384	NA	NA
Total	79	27.8696	1.00000	NA	NA

Pairwise PERMANOVA

**Bray-Curtis**						
pairs	Df	SumsOfSqs	F.Model	R2	p.value	p.adjusted
Ore1 fish vs water	1	2.8653	16.0252	0.2810	0.001	0.001
Nor1 fish vs water	1	1.8916	8.9838	0.4281	0.001	0.001
Nor2A fish vs water	1	1.8528	9.5086	0.4045	0.001	0.001
Nor2B fish vs water	1	1.3051	10.5174	0.6778	0.034	0.034
